# Supplementary material for: Biocatalytic stereocontrolled head-to-tail cyclizations of unbiased terpenes as a tool in chemoenzymatic synthesis
Source: Nat Commun. 2024 Jun 10;15:4925. doi: 10.1038/s41467-024-48993-9 (PMC11165016; doi:10.1038/s41467-024-48993-9)
Supplement: Supplementary file 1 — Supplementary Information [file 41467_2024_48993_MOESM1_ESM.pdf]

Supplementary information for

Biocatalytic stereocontrolled head-to-tail cyclizations of unbiased terpenes as a tool in chemoenzymatic synthesis

Andreas Schneider[1], Thomas B. Lystbæk[1], Daniel Markthaler[2], Niels Hansen[2] & Bernhard Hauer[1]*

[1]Institute of Biochemistry and Technical Biochemistry, University of Stuttgart, Stuttgart-Vaihingen, Germany

[2]Institute of Thermodynamics and Thermal Process Engineering, University of Stuttgart, Stuttgart-Vaihingen, Germany

This file contains:

Supplementary Figure 1-80

Supplementary Table 1-7

Supplementary Methods

General materials

General analytics

General methods

Computational methods

Preparative scale biotransformations

Chemical synthesis

Chromatograms

Mass spectra

NMR spectra

Amino acid sequences

Supplementary References

Table of contents

A. Supplementary Figures **3**
B. Supplementary Tables **21**
C. Supplementary Methods **25**
Chemicals *25*
Molecular biological kits *25*
Nuclear Magnetic Resonance *25*
Gas chromatography (GC) *25*
Circular dichroism *26*
Plasmid isolation *26*
Site-saturation mutagenesis *26*
Plasmid transformation via heat-shock method *26*
Expression of SHC in Erlenmeyer flasks *27*
Expression of SHC libraries in 96-DW plates *27*
Expression in 24 DW-plates *27*
Lyophilization protocol *27*
Thermolysis purification[25,26] *28*
SDS-PAGE *28*
Analytical biotransformations in GC screw-cap-vials *28*
Screening of AacSHC libraries via GC-MS *29*
Verification of promising hits *29*
Determination of turnover frequency and total turnover in vivo *29*
Fluorescence microscopy *30*
Docking simulations *30*
Free energy calculations *30*
Molecular model *30*
Simulation Details *31*
Double Decoupling *31*
D. Preparative scale biotransformations using lyophilized *E. coli* cells harboring the *Aac*SHC variants. **33**
E. Chemical synthesis **38**
F. Chromatograms **45**
G. Mass spectra **49**
H. NMR spectra **53**
I. Amino acid sequences **77**
J. Supplementary References **78**

A. Supplementary Figures

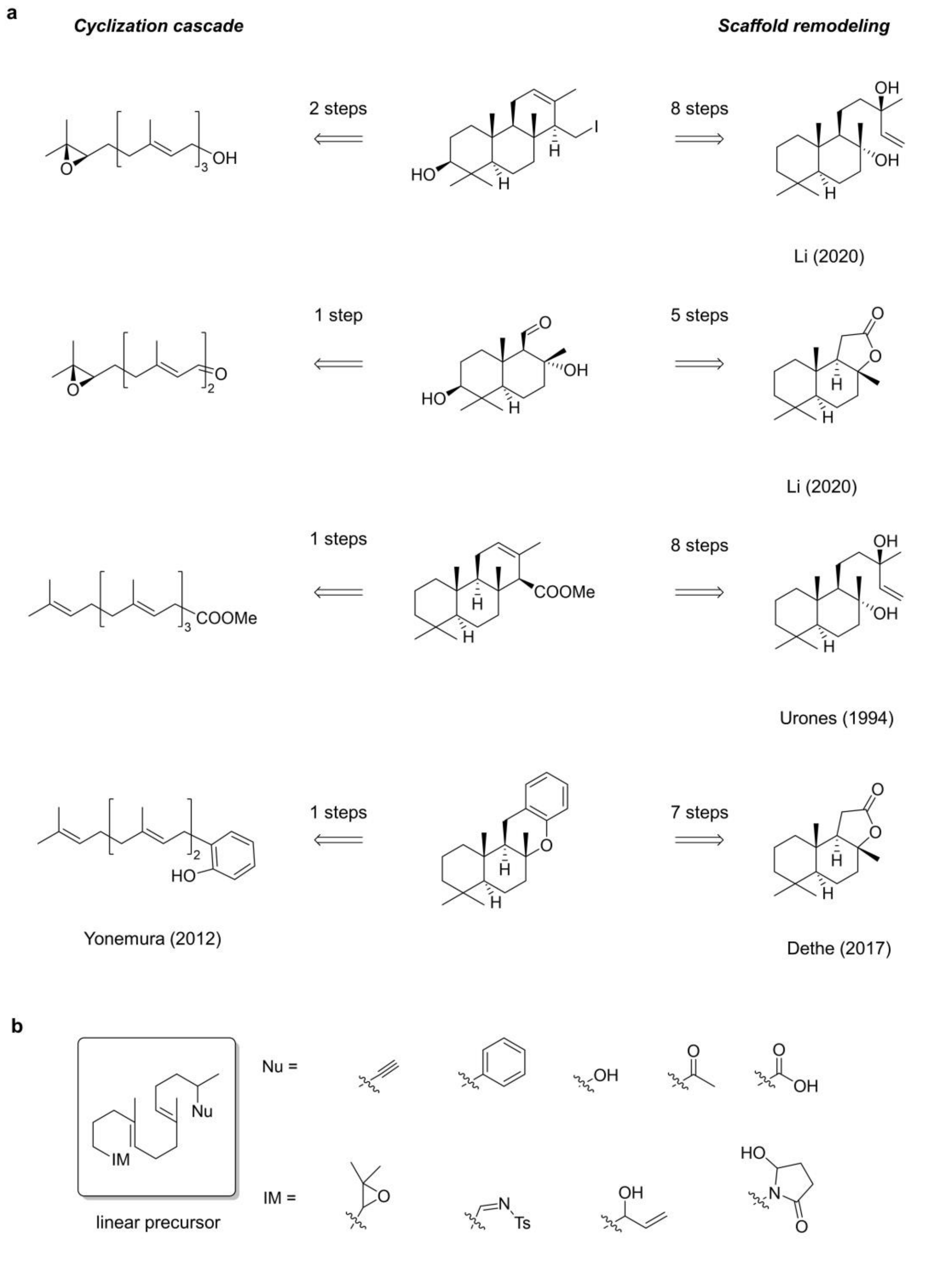

Supplementary Figure 1: (a) Selected examples from the literature in which a scalable and stereocontrolled cationic cyclization cascade could shorten the synthetic pathway to cyclic terpenes via scaffold remodelling.[1–4] (b) Selected ecamples of intiation motifs (IM) and terminating nucleophiles (Nu) required for succesful cyclization.[5]

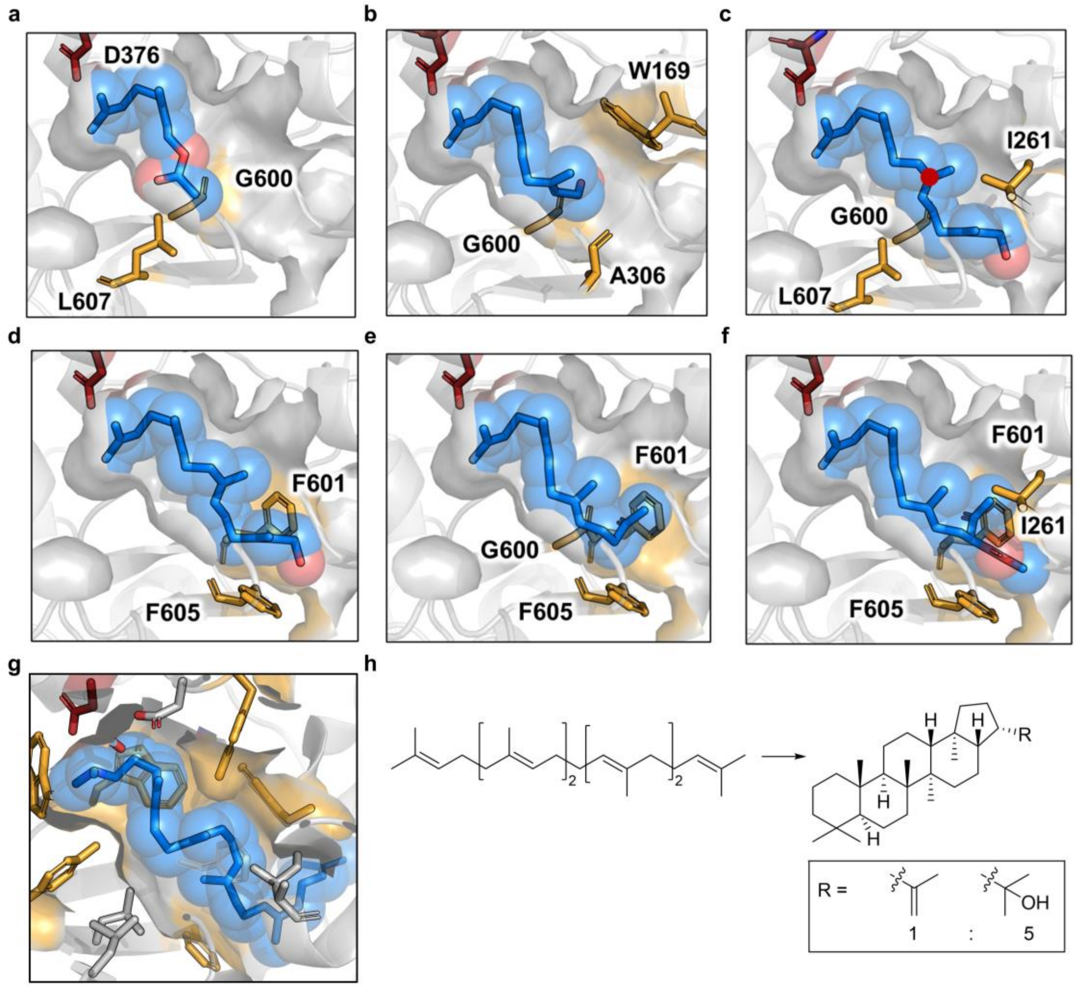

Supplementary Figure 2: Molecular docking of substrates (blue sticks and spheres) (a) 6, (b) 8, (c+d) 10, (e) 13 and (f) 15 into the active site of AacSHC (PDB: 1UMP) using YASARA[6] with AutoDock VINA. For comparison the crystal structure of the protein co-crystallized with aza-squalene is shown in (g) and aromatic residues are highlighted in orange. Protonating aspartate is shown as red sticks. For a-f) the most proficient hit positions of the screening are shown as orange sticks. The docking shows similar productive pre-foldings tightly packed in the active site of AacSHC for all substrates, which suggests the same expected stereochemistry. (h) Natural reaction is the AacSHC.

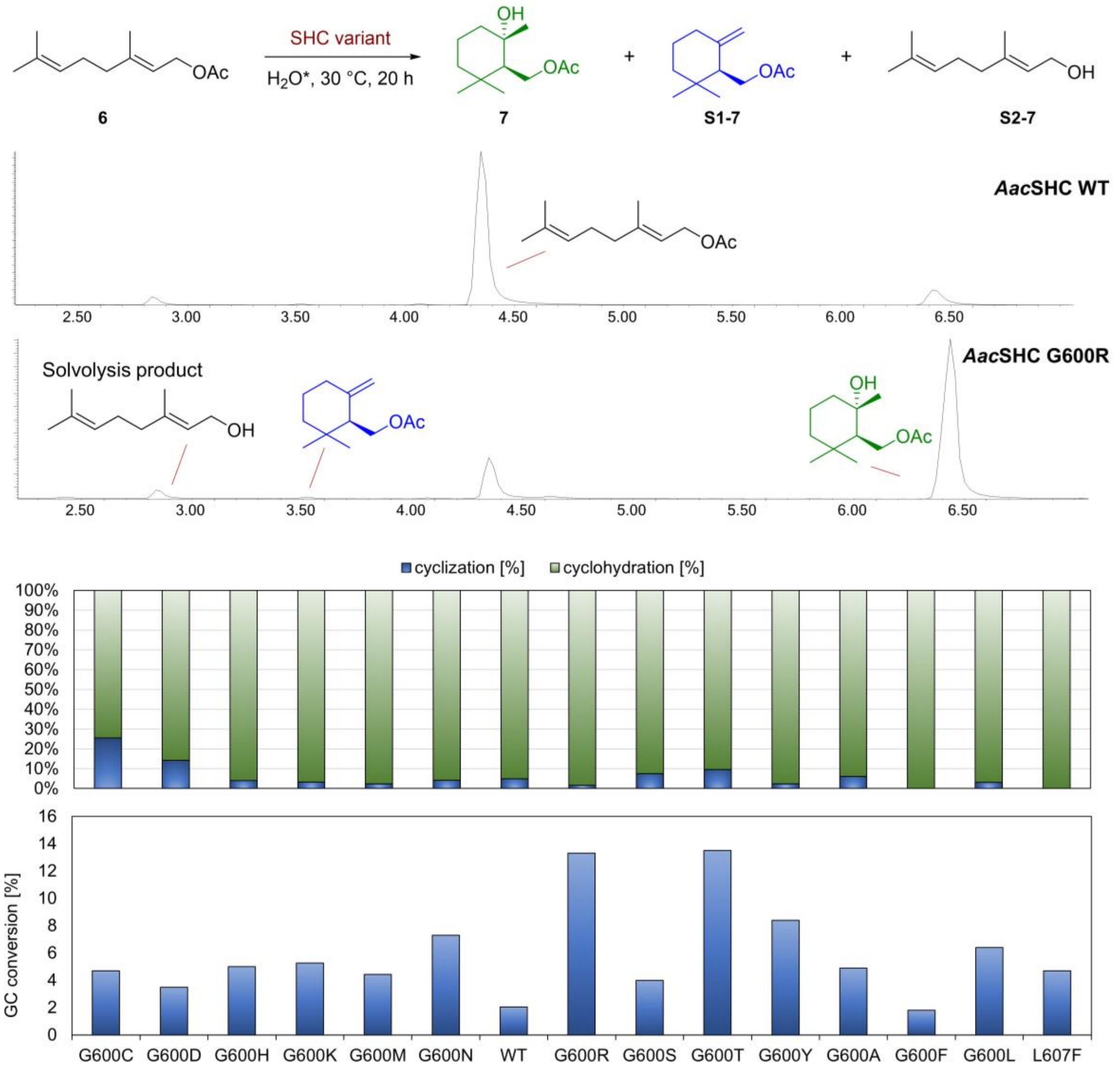

Supplementary Figure 3: Results of the internal SHC library screening for cyclization of E-geranyl acetate 6 to main product 7 and side products S1-7 and S2-7. Products were identified by NMR. Variants were plotted against their GC conversion (lower columns) and selectivity (upper columns). GC conversion and selectivity was determined as [Area_product]/[Area_substrate+Area_products]. Conditions: 1 mM substrate, E. coli cells expressing SHC variant, OD = 20, 30 °C, 20 h. Enzyme expression was controlled via SDS-PAGE. Best hits were then compared in analytical biotransformations in water.

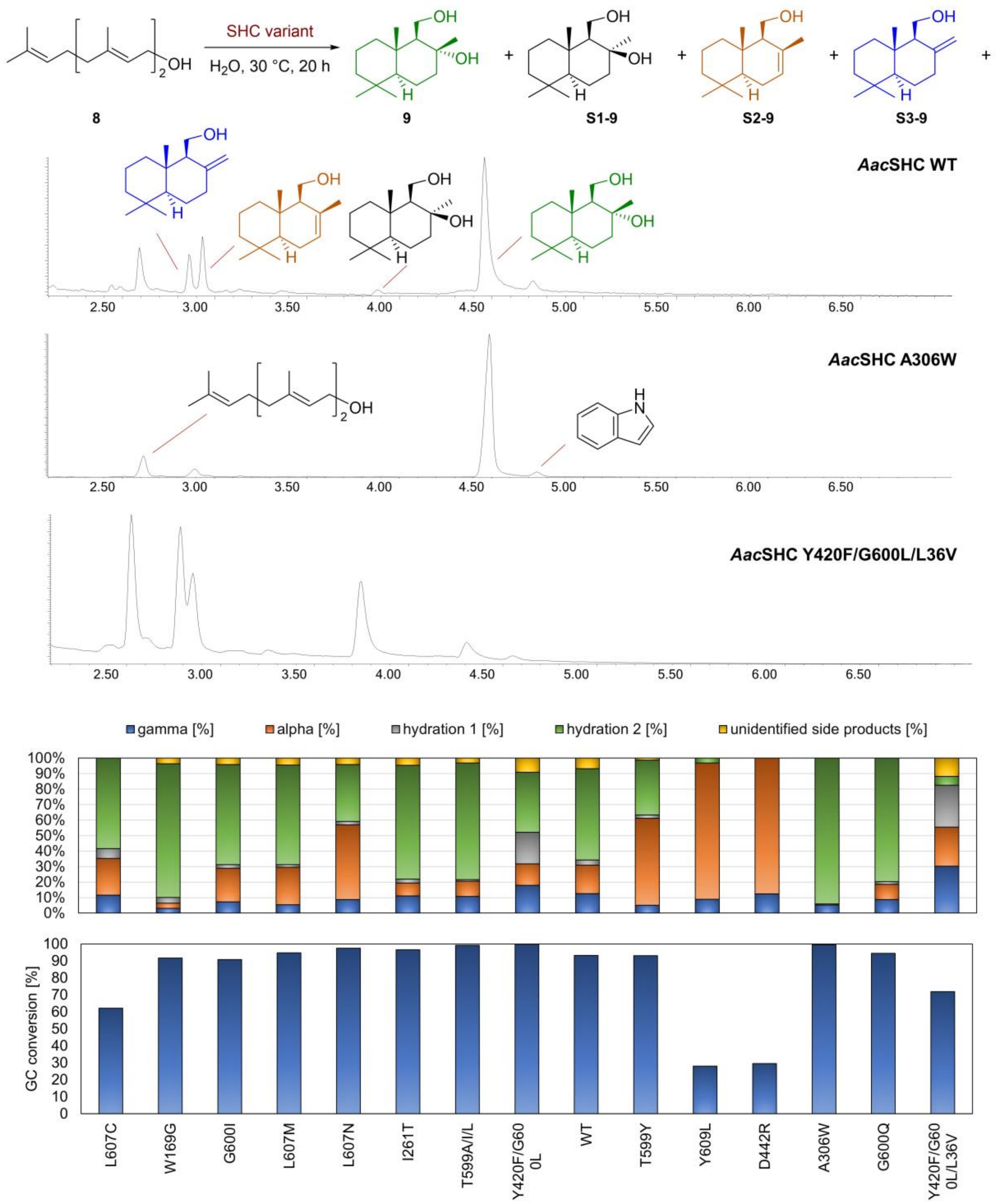

Supplementary Figure 4: Results of the internal SHC library screening for cyclization of E,E-farnesol 8 to desired cyclohydration product 9 and products S1-9, S2-9 and S3-9 among other unidentied side-products. Prodcuts were identified by NMR. Relative stereochemistry of S1-9 was determined by NOESY (see NMR section). Variants were plotted against their GC conversion (lower columns) and selectivity (upper columns). GC conversion and selectivity was determined as [Area_product]/[Area_substrate+Area_products]. Conditions: 1 mM substrate, E. coli cells expressing SHC variant, OD = 20, 50 °C, 20 h. Enzyme expression was controlled via SDS-PAGE. Best hits were then compared in analytical biotransformations in water.

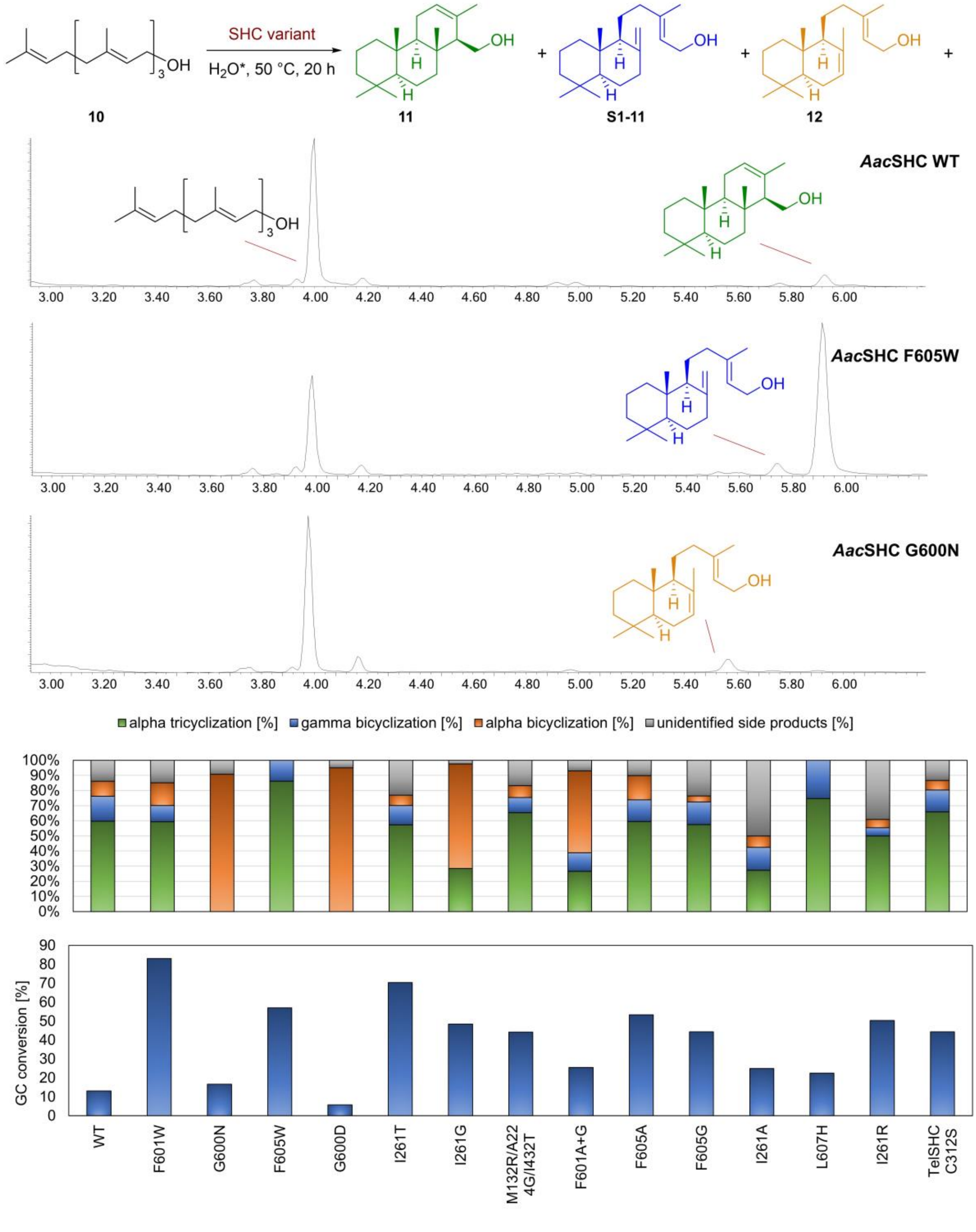

Supplementary Figure 5: Results of the internal SHC library screening for cyclization of E,E,E-geranyl geraniol 10 to desired products 11, 12 and side product S-11 among other unidentied side-products. Products were identifed by NMR. Variants were plotted against their GC conversion (lower columns) and selectivity (upper columns). GC conversion and selectivity was determined as [Area_product]/[Area_substrate+Area_products]. Conditions: 1 mM substrate, E. coli cells expressing SHC variant, OD = 20, 50 °C, 20 h. Enzyme expression was controlled via SDS-PAGE. Best hits were then compared in analytical biotransformations in water.

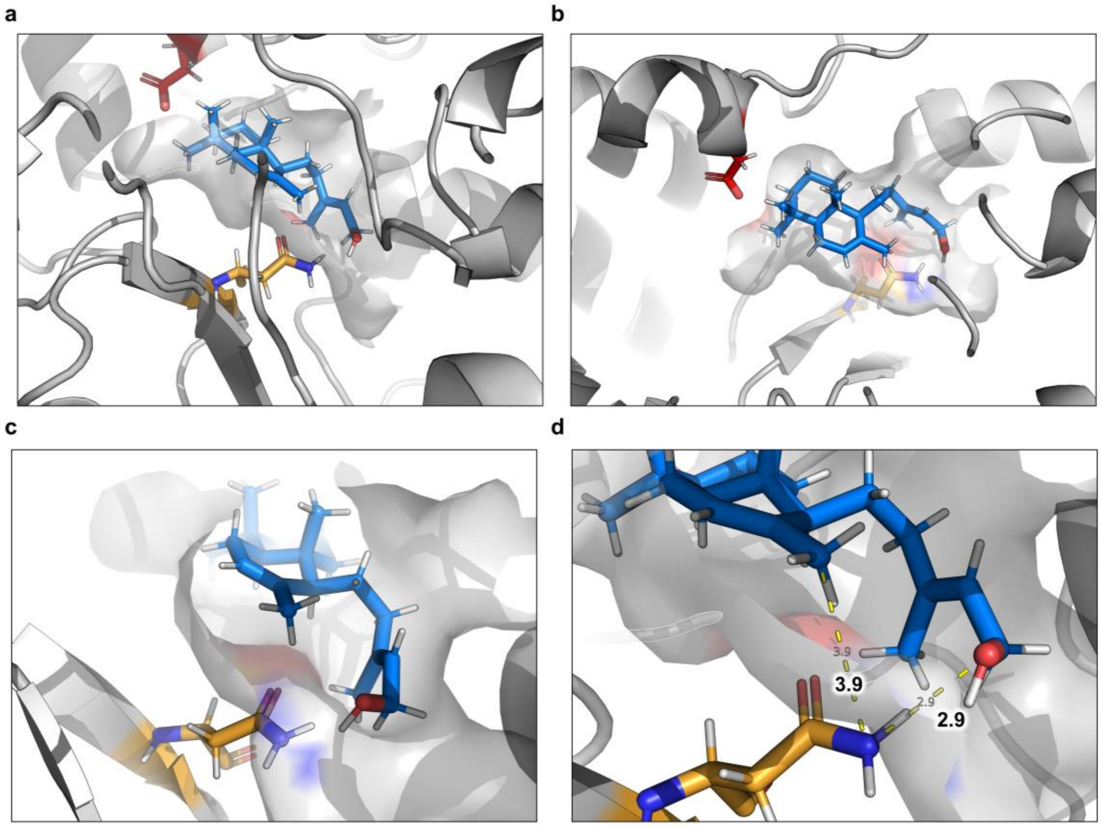

Supplementary Figure 6: Analysis of the docked labdane product (blue sticks) 12 into the active site of AacSHC G600N generated by RosettaFold.[7] Protonating aspartate shown as red and G600N shown as orange sticks (a) Side view. (b) Top view. (c) Frontal view. (d) Measurment of N-O and N-C distance shows proximity to both atoms, which suggests a dual-function of the introduced amino acid as an anchor[8] and/or Bronsted-base.[9]

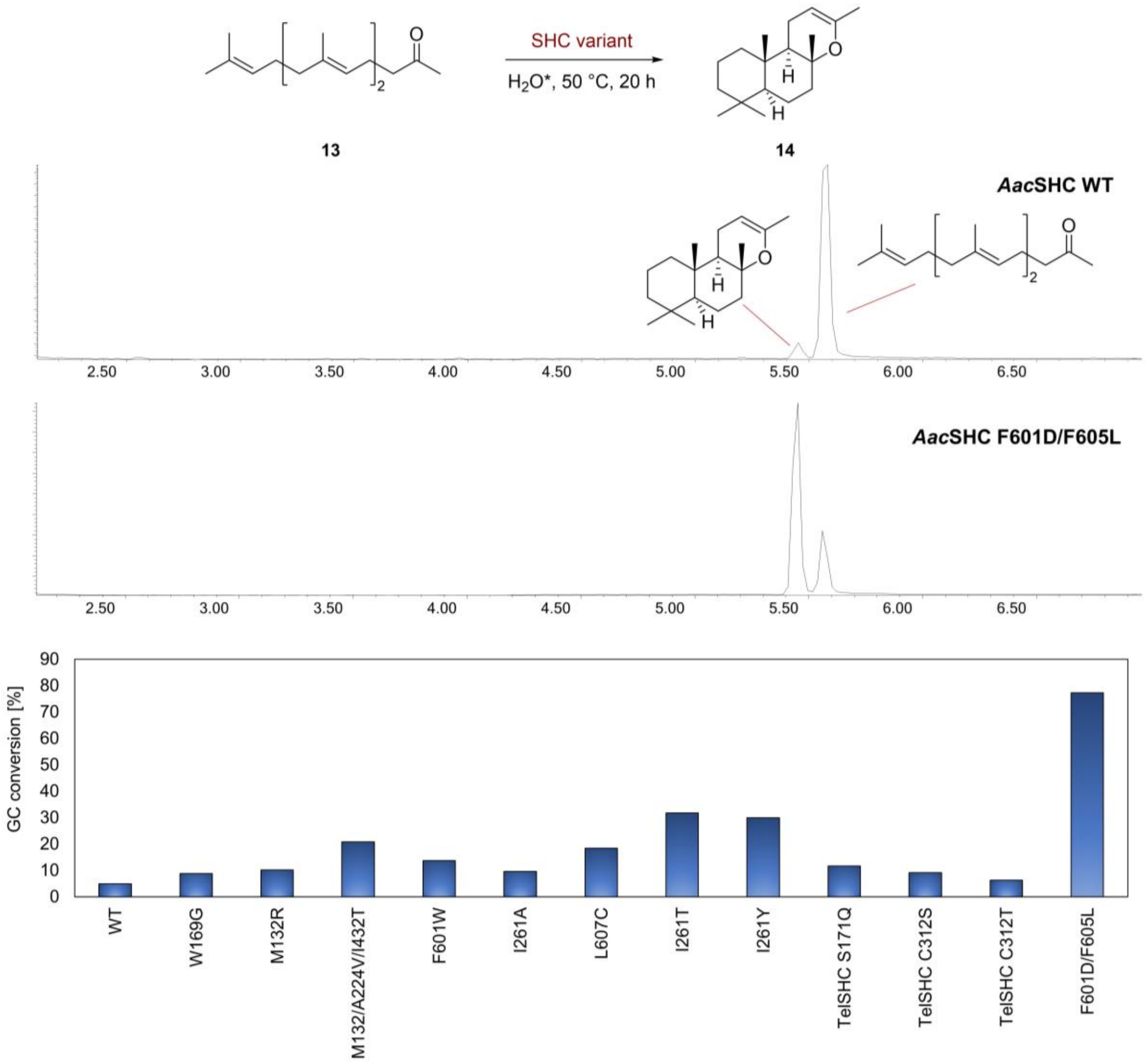

Supplementary Figure 7: Results of the internal SHC library screening for cyclization of E,E-farnesyl acetone 13 to sclareoloxide **14**. Products were identifed by NMR. Variants were plotted against their GC conversion (lower columns) and selectivity (upper columns). GC conversion and selectivity was determined as [Area_product]/[Area_substrate+Area_products]. Conditions: 1 mM substrate, E. coli cells expressing SHC variant, OD = 20, 50 °C, 20 h. Enzyme expression was controlled via SDS-PAGE. Best hits were then compared in analytical biotransformations in water.

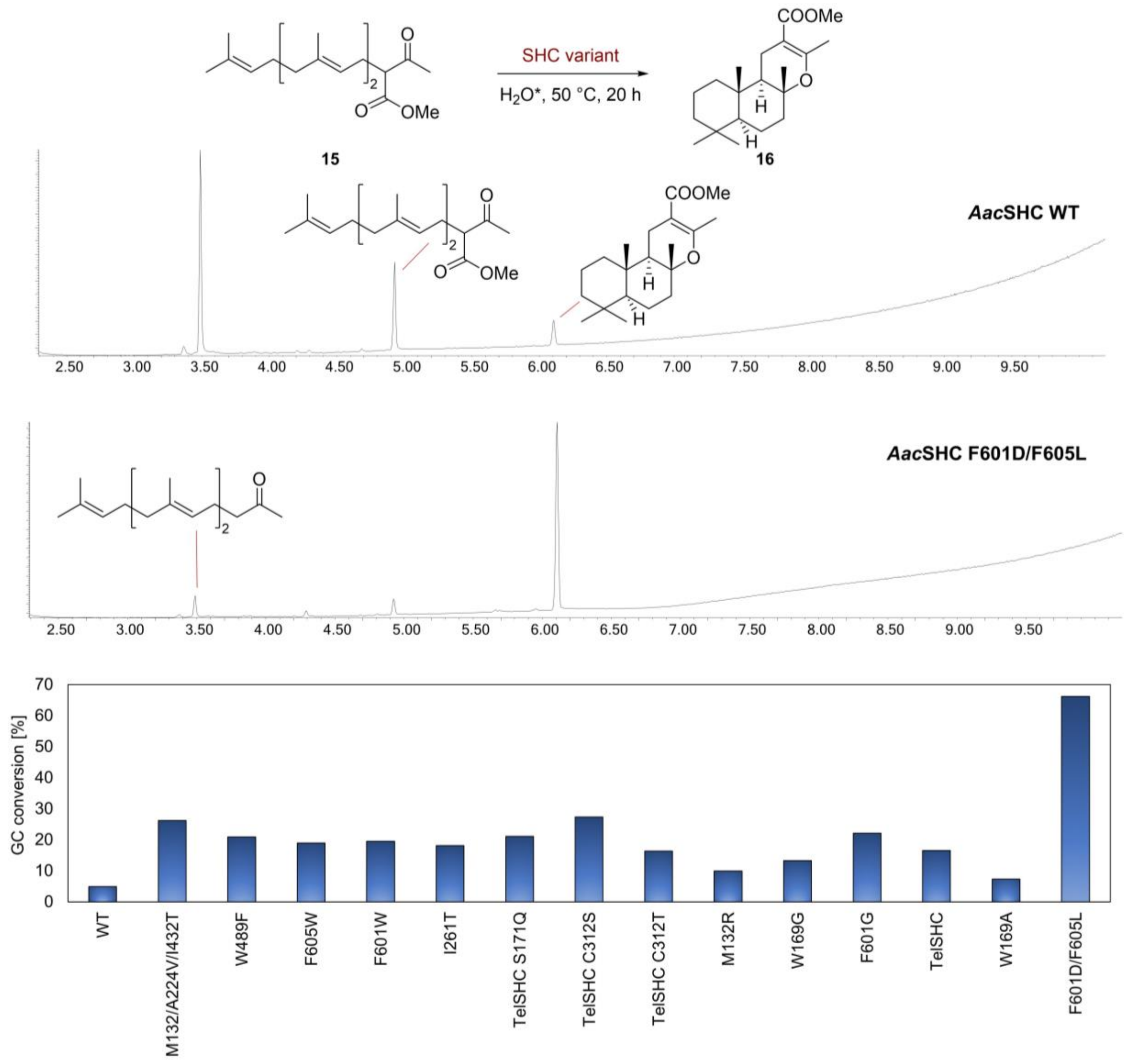

Supplementary Figure 8: Results of the internal SHC library screening for cyclization of ketoester 15 to product 16. Substrate 15 is prone to decarboxylate to 13 under GC injector conditions (300 °C). Products were identifed by NMR. Variants were plotted against their GC conversion (lower columns) and selectivity (upper columns). GC conversion and selectivity was determined as [Area_product]/[Area_substrate+Area_products]. Conditions: 1 mM substrate, E. coli cells expressing SHC variant, OD = 20, 50 °C, 20 h. Similar enzyme expression was determined via SDS-PAGE. Best hits were then compared in analytical biotransformations in water.

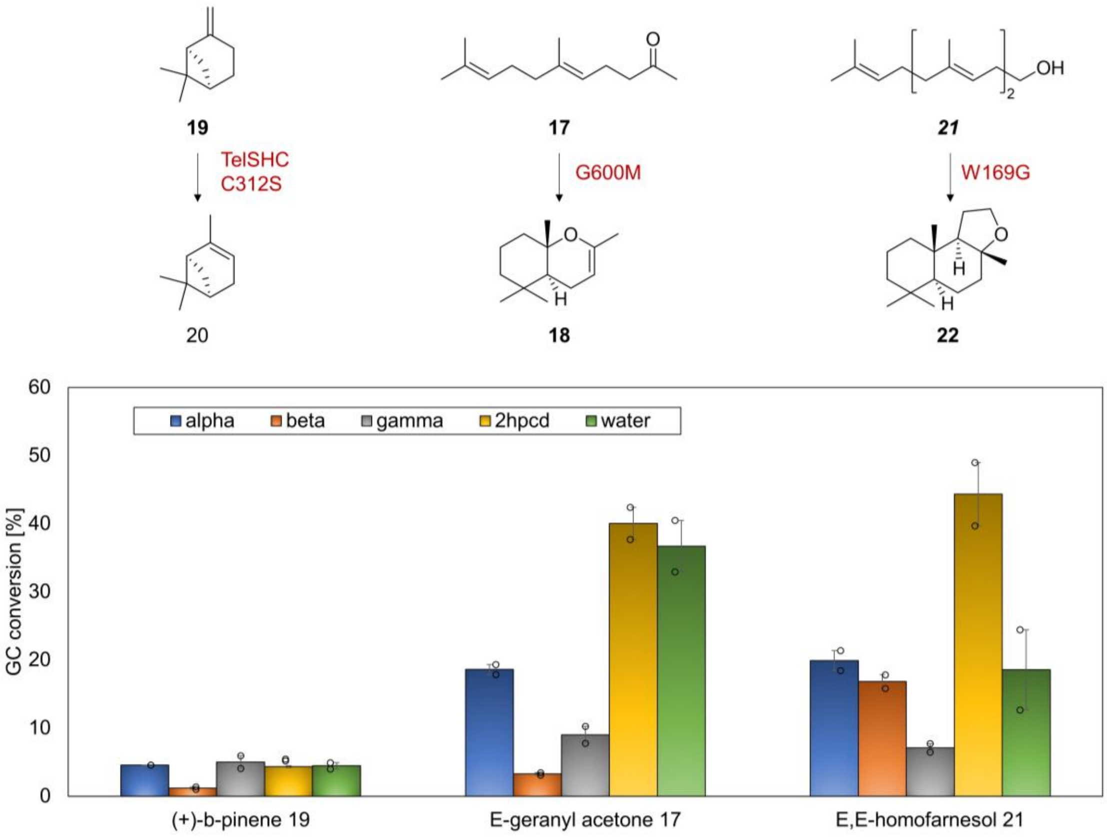

Supplementary Figure 9: Comparing α-, β-, γ-, and 2-hydroxypropyl-β-cyclodextrin in the biotransfromation of 19 to 20, 17 to 18 and 21 to 22 using different AacSHCs (red letters). Conditions: 10 g_{CDW}/L E. coli cells expressing the SHC variant (red letters), 1 mM substrate, 1 mM cyclodextrin, ddH_2O, 30 °C, 20 h. Reactions were performed in technical replicates n = 2 (dot plots). Bars represent mean values ± SD.

a

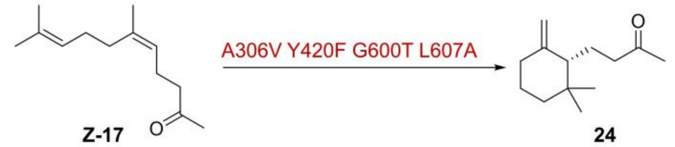

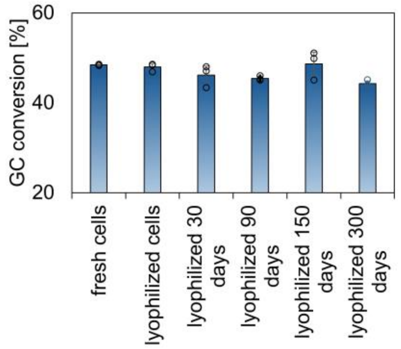

Sample no.	CDW [mg]	CWW [mg]	shrink-ing
1	107.1	23.1	4.64
2	82.1	18.39	4.46
3	92.7	24.24	3.82
4	105.8	16.4	6.45
5	111	23.15	4.79
6	81.1	17.91	4.53
Average	96.633	20.532	4.78

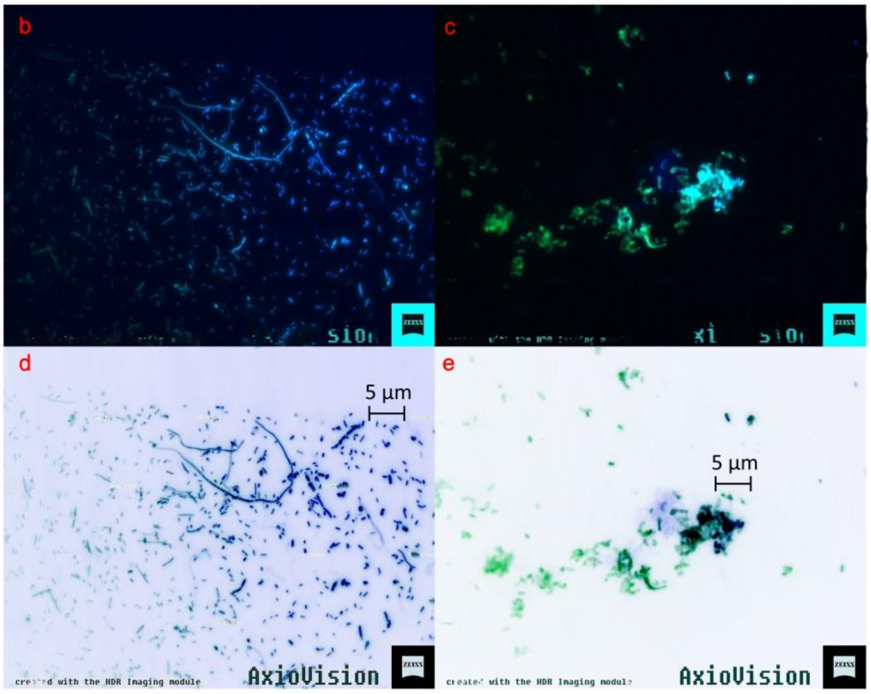

Supplementary Figure 10: Investigation on the SHC biocatalyst preparation as lyophilized E.coli cells. (a) Model reaction of Z-17 monocyclization to (–)-γ-dihydroionone 24 with fresh cells and lyophilized cells shows almost no change in activity even after over 300 days. Average shrinking factor of fresh E. coli cells was determined with ~4.8. Reactions were performed in technical replicates n = 3 (dot plots). Bars represent mean values ± SD. (b) Overlay of fluorescence microscope pictures taken from fresh E. coli cells stained with DAPI (blue) and SYTOX (green) demonstrates high abundance of living (blue) cells. (c) Overlay of fluorescence microscope pictures taken from lyophilized E. coli cells stained with DAPI (blue) and SYTOX (green) demonstrates high abundance of dead (green) cells or a mixture of living and dead cells (cyan). (d) and (e) are the negatives of (b) and (c). For details on Fluorescence microscopy please see section below.

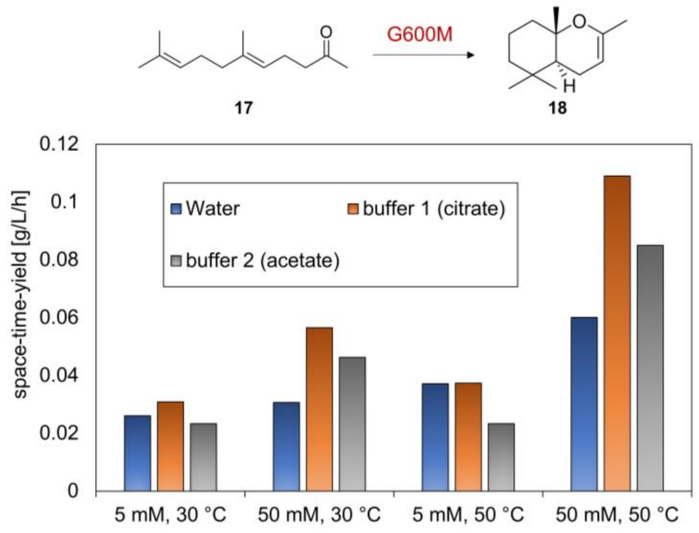

Supplementary Figure 11: Biotransformation of 17 to 18 using either water, 100 mM acetate or 100 mM citrate buffer at pH = 6.0 compared in their space-time-yields. Conditions: 10 g_{CDW}/L E. coli cells expressing the SHC variant (red letters), substrate, 20 h. Space-time-yield was determined by [GC conversion]·[$c_{substrate}$]/[time].

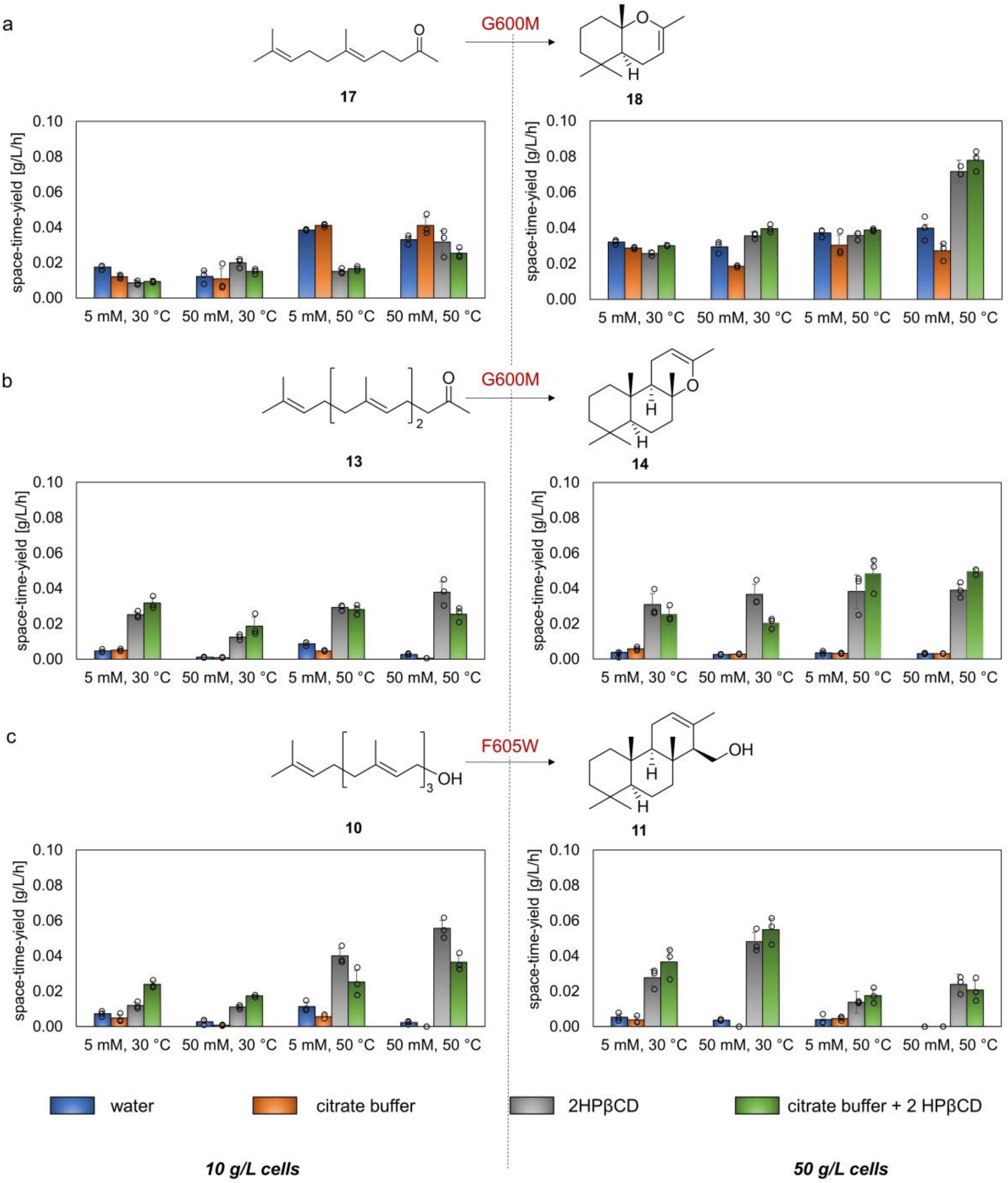

Supplementary Figure 12: Biotransformation of substrates 17 (a), 13 (b) and 10 (c) using 10 (left side) or 50 (right side) g_{CDW}/L E. coli cells expressing the corresponding SHC variant (red letters), different substrate concentrations (5 and 50 mM), temperatures (30 and 50 °C) and additives (water = blue bars, citrate buffer = orange bars, 2HPCD = grey bars, citrate buffer + 2HPCD = green bars; citrate buffer = 100 mM citric acid, pH = 6.0, 2HPβCD = 2-hydroxypropyl-β-cyclodextrin). Reactions were performed in technical replicates n = 3 (dot plots). Bars represent mean values ± SD.

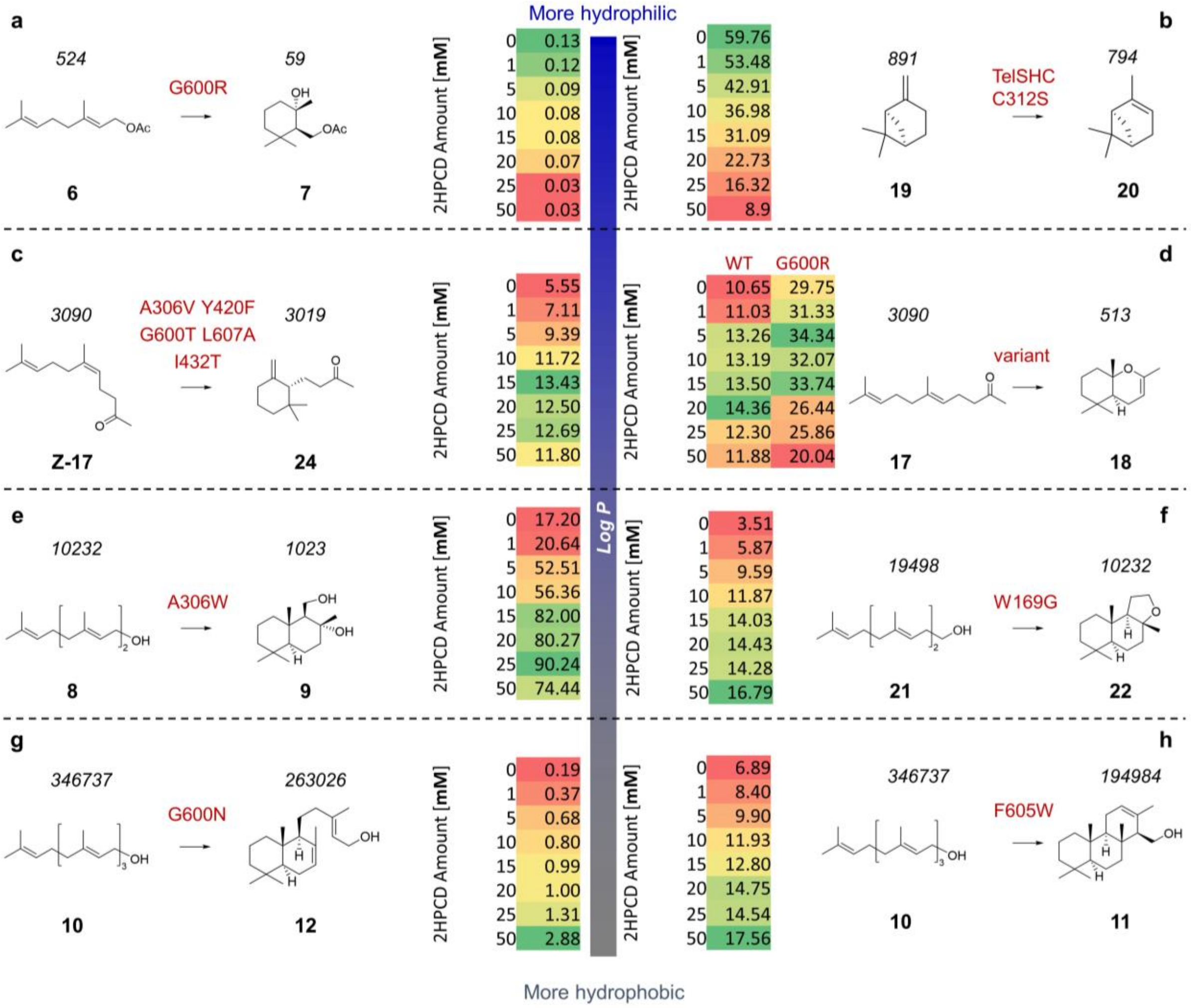

Supplementary Figure 13: Comprehensive evaluation of the beneficial encapsulation effect of 2-hydroxypropyl-β-cyclodextrin (2HPCD) on the biotransformation using substrates 6 (a), 19 (b), Z-17 (c), 17 (d), 8 (e), 21 (f), 10 to 12 (g), 10 to 11 (h) and the identified favorable SHC variants (red letters). Conditions: 20 mM substrate, 10 g_{CDW}/L cells, ddH_2O, 50 °C. The brackets show the GC conversion of the substrates after 20 h. Green designates better conversion. The results demonstrate that with increasing P (= 10^{logP}, black italic numbers above substrates) and thus increasing hydrophobicity the beneficial effect of cyclodextrin increases as well. LogP was calculated by ChemDraw.

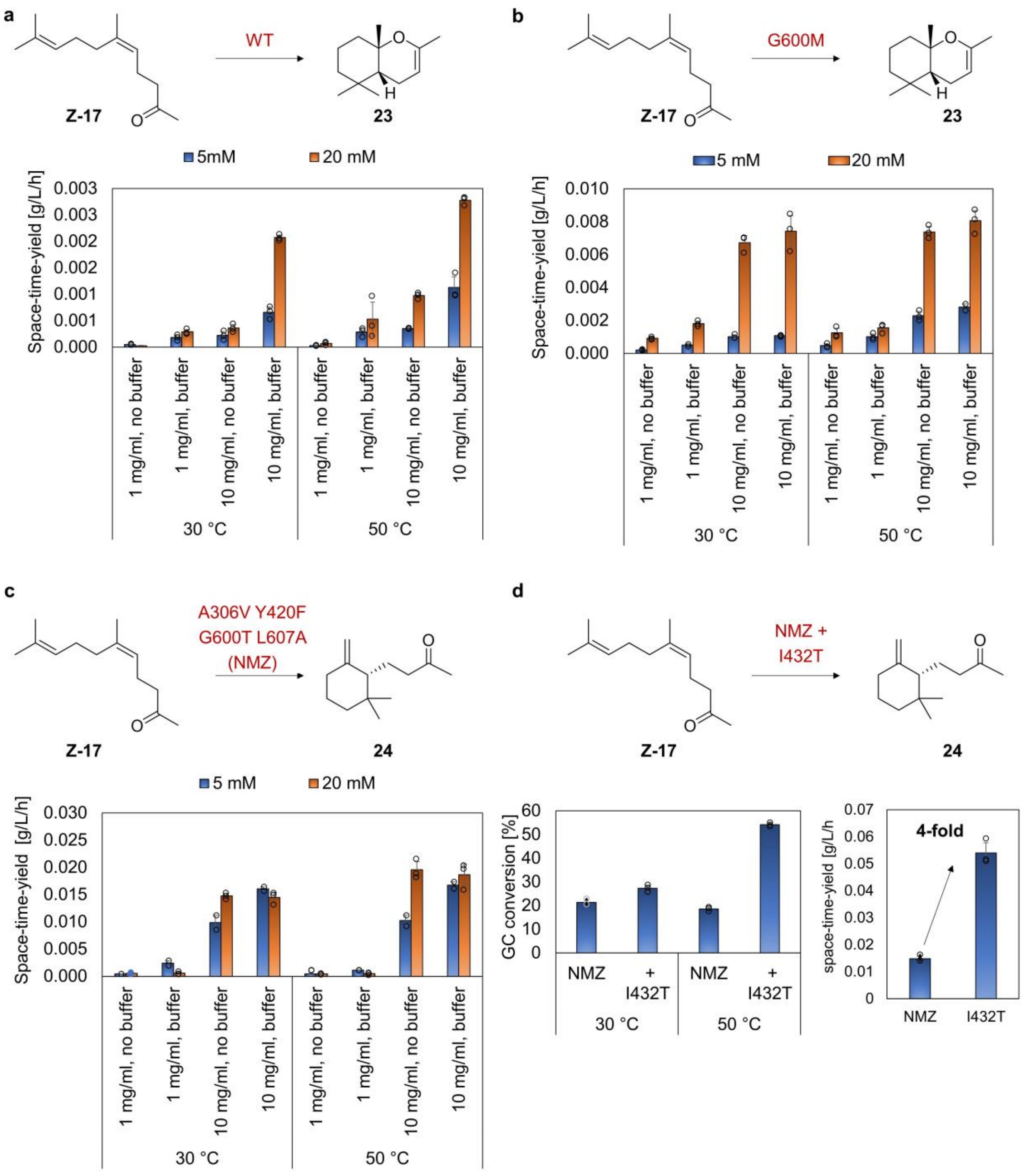

Supplementary Figure 14: Biotransfromations of Z-17 using different SHC variants (red letters) at differing conditions. (a) Biotransformation of Z-17 with AacSHC WT shows increased space-time yields at higher temperatures (50 °C) and higher substrate concentration (20 mM). (b) Single-point mutant G600M of AacSHC still shows improved space-time-yields at higher temperatures (50 °C) and higher substrate concentration (20 mM). (c) Quadruple mutant NMZ shows some lowered space-time-yields higher substrate concentration (20 mM). (d) Pentuple variant NMZ+I432T, which was identified by CAVER[10] analysis and subsequent site-saturation mutagenesis in the designated area (see ref. [11] for more information on this mutagenesis strategy), again showed increased GC conversion at higher temperature entailing 4-fold increased space-time-yield at high substrate concentration. Conditions for right graph: 10 g_{CDW}/L cells, 50 mM substrate, 30 °C, ddH_2O 20 h. Reactions were performed in technical replicates n = 2 or 3 (dot plots). Bars represent mean values ± SD.

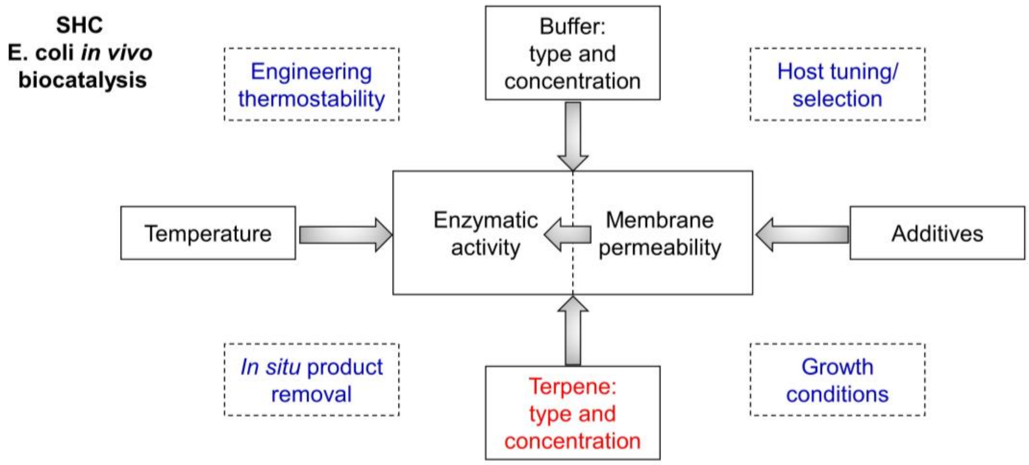

Supplementary Figure 15: Abiotic stress model of SHC biocatalysis. Influences that can affect the system consisting of enzyme and membrane include temperature, additives, terpene and buffer. The system can be augmented by engineering thermostability of the enzyme (see ref. [12] and Figure S14), in situ product removal techniques,[13] cell constitution by means of growth conditions and the enzyme's host.[14] Membrane fluidity can potentially affect enzymatic activity as well.[11,15] Terpene type (hydrophobicity) and concentration are strongly influencing the system.

a

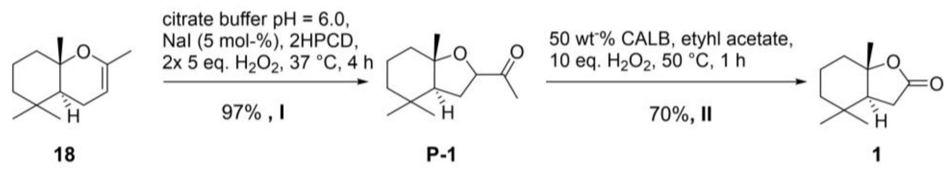

partial reaction	entry	variation from standard conditions	yield [%]
	1	water instead of citrate buffer	5*
	2	EtOAc instead of water	2*
I	3	1 mol-% NaI	12*
	4	No 2HPCD	10*
	5	5 eq. H_2O_2	61†
	6	10 eq. H_2O_2 at once	77†
	7	mCPBA instead of CALB/H_2O_2	54
	8	No H_2O_2	0
II	9	No CALB	0
	10	50:50 H_2O:etyhl acetate	21
	11	30 °C	0

b

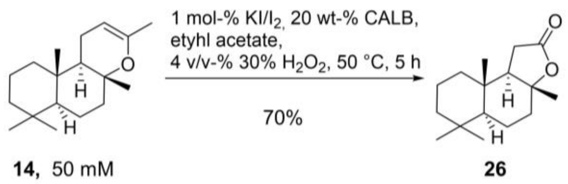

entry	variation from standard conditions	yield [%]
1	100 mM substrate, 5 mol-% KI/I_2	25*
2	100 mM substrate, 5% KI/I_2, 10 eq H_2O_2	12*
3	50 mM substrate, 5 eq. H_2O_2	42
5	0.5 mol-% KI/I_2	53
6	50 wt-% CALB	60 (3h reaction time)
7	45 °C	0
8	water instead of 30% H_2O_2	0
9	no CALB	0

Supplementary Figure 16: Evaluation of optimal reaction condtions for (a) the iodine reaction and peroxidative rearrangement separately and (b) in one-pot. 2-hydroxpropyl-β-cyclodextrin = 2HPCD. Yields are given as isolated yields. *GC yield was directly determined from $AREA_{product}/ (AREA_{substrate}+AREA_{product})*100$. †crude yield

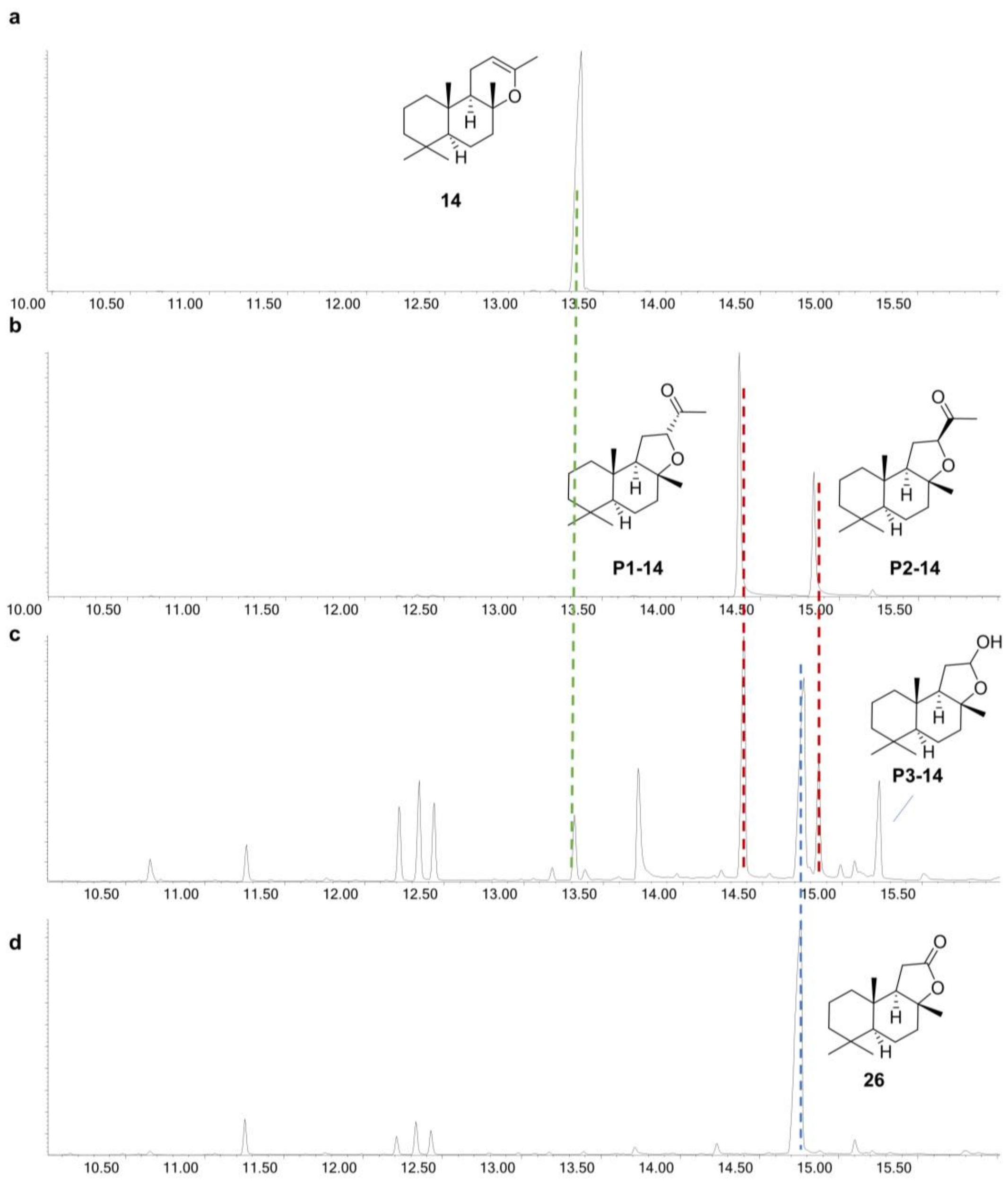

Supplementary Figure 17: Reaction control of the one-pot iodine/lipase rearrangement. (a) Substrate 14. (B) Ketone Intermediates P1-14 and P2-14. Major isomer was determined via NOESY (see NMR section) (c) Reaction chosing bad condtions and showing side-product sclaral P3-14, which presumably accumulates during the cleavage of a dimeric lactol anhydride (for more information on this mechanism please see ref. [16]. d) Reaction under optimized conditions yielding high amounts of 26.

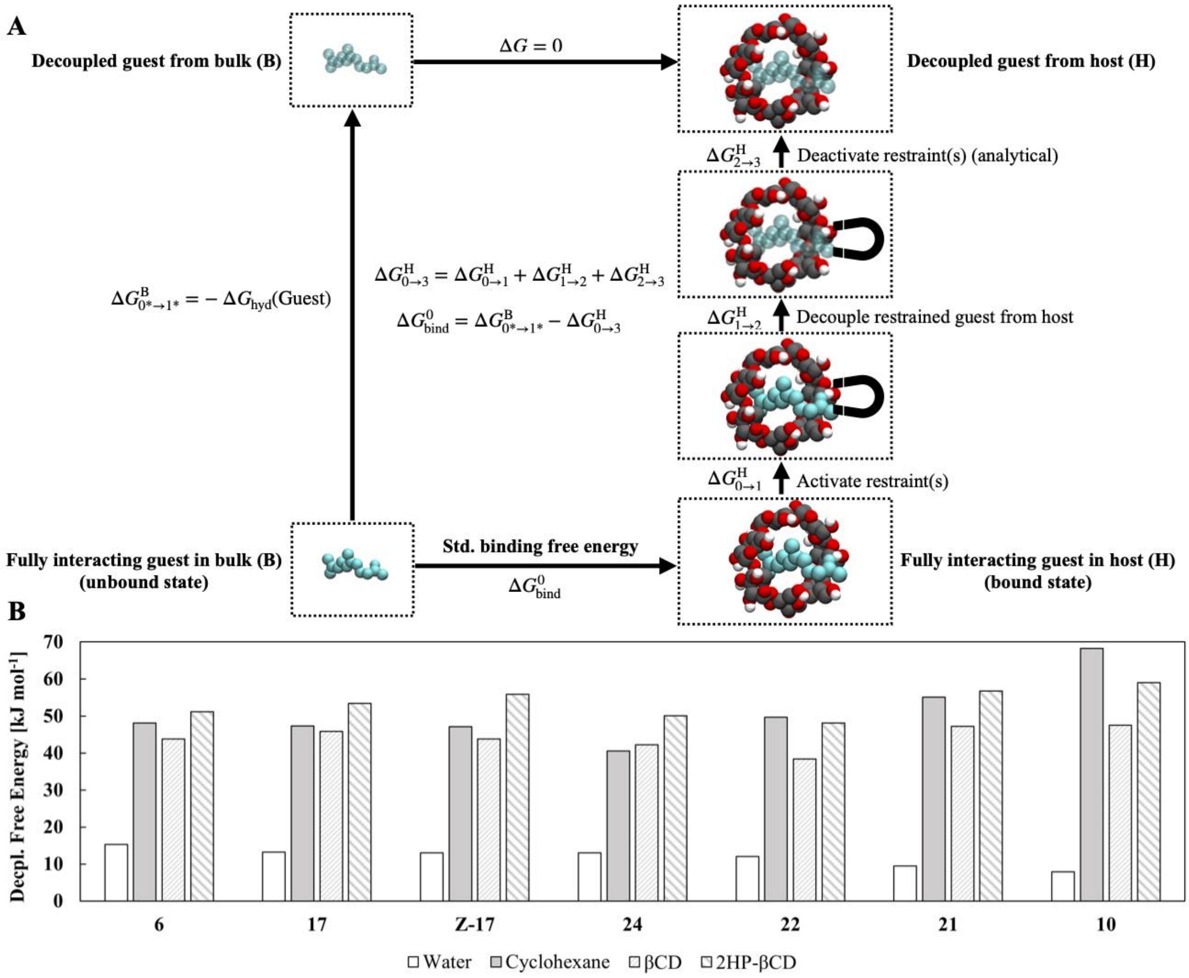

Supplementary Figure 18: (A) Thermodynamic cycle as applied in the double decoupling methodology (DDM). Free energy differences (ΔG) for the relevant steps are denoted next to the arrows. Left vertical branch: substrate or product (both denoted as guest) molecule is decoupled from the bulk solvent environment (superscript B) keeping intact the intramolecular interactions. In case of water, this transfer free energy from solvent to an ideal gas phase $\Delta G^{\mathrm{B}}_{0*\to1*}$ corresponds to the negative hydration free energy of the guest molecule. Right vertical branch: guest molecule is decoupled from the host binding site (superscript H). $\Delta G^{\mathrm{H}}_{0\to1}$ represents the free energy change associated with activating a restraint between the host and the fully interacting guest; $\Delta G^{\mathrm{H}}_{1\to2}$ represents the free energy change associated with the process of decoupling the restrained guest within the host binding site while keeping the intramolecular interactions intact; $\Delta G^{\mathrm{H}}_{2\to3}$ accounts for the contribution of releasing the restraint from the non-interacting guest, which can be evaluated analytically. (B) Estimated decoupling free energies according to (A) from MD simulations for various guest molecules (denoted by their IDs) in different environments: bulk water (white), cyclohexane (grey), bound complex with β-cyclodextrin (βCD) (fine grey stripes), bound complex with 2-hydroxypropyl-β-cyclodextrin (2HP-βCD) (thick grey stripes). For water and cyclohexane, the free energy difference corresponds to $\Delta G^{\mathrm{B}}_{0^*\to1^*}$, whereas the decoupling from the host molecules corresponds to the sum of the free energy differences, $\Delta G^{\mathrm{H}}_{0\to3}$, of the right branch of the thermodynamic cycle (c.f. A). Statistical errors of the free differences were estimated with the MBAR estimator and are bound within +/- 1.5 kJ mol^{-1}. Guest molecules are sorted by ascending size from left to right, with 12 C-atoms for geranyl acetate (6), 13 C-atoms for geranyl acetone (17), neryl acetone (Z-17), γ-dihydroionone (24), 16 C-atoms for ambroxide (22), homofarnesol (21) and 20 C-atoms for geranyl geraniol (10), respectively.

B. Supplementary Tables

Supplementary Table 1: Selected examples of cationic cyclizations from the literature using the same substrates as in this study.

Substrate	Study	Catalyst	Issues
6	Vlad[17]	FSO_3H	requires kryogenic conditions (-78 °C), low product selectivity, stereospecfic, substrate decomposition and polymerization
6	Vlad[18]	Hg	stereospecific, toxic mercury, requires demercuration
6	Tsangarakis[19]	NaY	low product selectivity, low stereocontrol
8	Vlad[17,20,21]	FSO_3H	requires kryogenic conditions (-78 °C), low product selectivity, stereospecfic, substrate decomposition and polymerization, no hydration product
10 to 11	Vlad[17,20]	FSO_3H	requires kryogenic conditions (-78 °C), low product selectivity, stereospecfic, substrate decomposition and polymerization
10 to 12	-		
13	-		
15	Parker[22]	Hg	moderate yield, toxic mercury, requires demercuration, stereospecific

Supplementary Table 2: Summary of upscales of this study.

substrate	density [g/mL]	MW [g/mol]	mol [mol]	mass [g]	[mL]	variant	volume [L]	Temperature [°C]	additives	substrate amount [mM]	substrate amount [g/L]	cell amount [g/L]	SHC amount [g/L]	SHC amount [mM]	catalyst loading [mol-%]	conversion [%]	yield [g]	yield [%]	time [h]	TTN	TOF [h^{-1}]
dihydroionone 24	0.87	194	0.0224	4.35	5.00	A306V/Y420F/G600T/L607A/I432T	1.0	30	15 mM 2HPCD	22.4	4.35	10	1.8	0.03	1.12	97	3.87	89	120	863	7.2
cyclogeranyl acetate hydrate 7	0.87	196	0.0011	0.22	0.25	G600R	0.1	50	-	11.1	2.18	10	2.5	0.03	3.15	95	0.15	71	480	301	0.6
drimendiol 9	0.88	222	0.0019	0.43	0.49	A306W	0.1	30	25 mM 2HPCD	19.4	4.3	10	1.5	0.02	1.08	96	0.34	80	72	888	12.3
drimendiol S1-9	0.88	222	0.0019	0.43	0.49	L36V/Y420F/G600L	0.1	30	25 mM 2HPCD	19.4	4.3	10	2.5	0.03	1.80	90	0.30*	70	192	500	2.6
Labdane 12	0.89	290	0.0008	0.22	0.25	G600N	0.1	50	50 mM 2HPCD	7.7	2.2	10	3.2	0.04	5.84	95	0.17	78	216	163	0.8
ent-iscopalol 11	0.89	290	0.0008	0.22	0.25	F605W	0.1	50	50 mM 2HPCD	7.7	2.2	10	1.7	0.02	3.10	91	0.18	79	96	293	3.1
β-keto ester 16	0.87	320	0.0007	0.22	0.25	F601D/F605L	0.1	50	50 mM 2HPCD	6.8	2.2	10	2.5	0.03	5.15	96	0.17	80	96	186	1.9
trans-hexahydro-chromene 18	0.87	194	0.0011	0.22	0.25	G600M	0.1	50	100 mM citrate buffer	11.2	2.2	10	2.1	0.03	2.62	98	0.20	90	96	374	3.9
cis-hexahydro-chromene 23	0.87	194	0.0011	0.22	0.25	G600M	0.1	50	100 mM citrate buffer	11.2	2.2	10	2.1	0.03	2.62	90	0.18	82	120	343	2.9
sclareoloxide 14	0.87	262	0.0017	0.44	0.50	F601D/F605L	0.1	50	50 mM 2HPCD	16.6	4.4	10	2.5	0.03	2.11	99	0.40	92	72	470	6.5

*crude yield

Supplementary Table 3: List of buffers used in this work.

Buffer	Ingredients
10x phosphate buffer (KP_i-buffer)	0.17 M KH_2PO_4, 0.72 M K_2HPO_4, pH=7.4
lysis buffer	200 mM citric acid, 0.1% EDTA, pH=6.0
extraction buffer	100 mM citric acid, 1% CHAPS, pH=6.0
citrate buffer	100 mM citric acid, pH=6.0
acetate buffer	100 mM acetate acid, pH=6.0
cyclodextrin buffer	5-50 mM 2HPCD, 100 mM citric acid, pH=6.0
PBS buffer	137 mM NaCl, 2.7 mM KCl, 10 mM Na_2HPO_4 pH=7.4

Supplementary Table 4: List of media used in this work.

Medium	Ingredients
Lysogeny broth (LB)	10 g/L tryptone, 10 g/L NaCl, 5 g/L yeast extract
Auto-induction medium (T-DAB)	12 g/L tryptone, 24 g/L yeast extract, 2.9 g/L glucose, 11.1 g/L Glycerol, 7.6 g/L Lactose

Supplementary Table 5: List of primers used in this work.

Entry	Name	Sequence (5´→3´) Forward/Reverse
1	AacSHC I432NNK	GCGATCTGCCGAACCATNNKCCGTTTTGCGATTTTGGC/ GCCAAAATCGCAAAACGGMNNATGGTTCGGCAGATCGC

Supplementary Table 6: Summary of estimated decoupling free energies ($\Delta G^{B}_{0*\rightarrow 1*}$) of the guest molecules in bulk solvent (water and cyclohexane) according to Fig. S18 (a). The value of $\Delta G^{B}_{0*\rightarrow 1*}$ corresponds to the negative solvation free energy in the corresponding solvent. Explanations for the applied abbreviated guest names can be found in the corresponding methods section. All ΔG values are given in kJ·mol^{-1}.

Guest	Water		Cyclohexane		$\Delta G_{H2O\text{->}CH}$	
	mean	+/-	mean	+/-	mean	+/-
6	15.26	0.10	48.10	0.70	-32.84	0.71
10	7.92	0.14	68.20	1.70	-60.28	1.71
17	13.19	0.11	47.30	0.90	-34.11	0.91
Z-17	13.00	0.11	47.10	1.10	-34.10	1.11
22	12.04	0.11	49.70	0.50	-37.66	0.51
21	9.47	0.12	55.10	1.10	-45.63	1.11

24	13.00	0.15	40.60	1.00	-27.60	1.01

Supplementary Table 7: Summary of estimated free energy differences according to Fig. S18 (A) for different combinations of host and guest molecules forming a 1:1 complex solvated in water. Explanations for the applied abbreviated guest and host names can be found in the corresponding methods section. All ΔG values are given in kJ mol[-1]. The standard binding free energy was calculated as $\Delta G^{0}_{\mathrm{bind}} = \Delta G^{\mathrm{B}}_{0^{*}\to 1^{*}} - \Delta G^{\mathrm{H}}_{0\to 3}$, where $\Delta G^{\mathrm{H}}_{0\to 3}$ is given by the sum: $\Delta G^{\mathrm{H}}_{0\to 3} = \Delta G^{\mathrm{H}}_{0\to 1} + \Delta G^{\mathrm{H}}_{1\to 2} + \Delta G^{\mathrm{H}}_{2\to 3}$. Values for the decoupling free energies in bulk water ($\Delta G^{\mathrm{B}}_{0*\to 1*}$) were taken from Tab. S7. Uncertainties for $\Delta G^{\mathrm{H}}_{0\to 3}$ and $\Delta G^{0}_{\mathrm{bind}}$ were estimated from the corresponding statistical uncertainties of $\Delta G^{\mathrm{H}}_{0\to 1}$, $\Delta G^{\mathrm{H}}_{1\to 2}$ and $\Delta G^{\mathrm{B}}_{0^{*}\to 1^{*}}$ according to standard error propagation rules.

Host	Guest	Guest ID	$\Delta G^{H}_{0\text{->}1}$		$\Delta G^{H}_{1\text{->}2}$		$\Delta G^{H}_{2\text{->}3}$	$\Delta G^{H}_{0\text{->}3}$		ΔG^{0}_{bind}	
			mean	+/-	mean	+/-		mean	+/-	mean	+/-
αCD	GACE	17	1.79	0.01	46.27	0.02	-6.80	41.25	0.02	-28.06	0.11
αCD	NACE	Z-17	0.70	0.00	39.95	0.26	-6.80	33.85	0.26	-20.85	0.28
αCD	AMB	22	54.15	0.15	-13.25	0.19	-6.80	34.10	0.24	-22.06	0.26
αCD	HFL	21	7.08	0.02	44.80	0.17	-6.80	45.08	0.17	-35.61	0.21
βCD	GAC	6	2.15	0.04	48.42	0.17	-6.80	43.77	0.17	-28.51	0.20
βCD	GACE	17	1.26	0.02	51.37	0.18	-6.80	45.83	0.18	-32.64	0.21
βCD	NACE	Z-17	0.74	0.03	49.89	0.18	-6.80	43.83	0.18	-30.83	0.21
βCD	C13P	24	7.37	0.09	41.71	0.20	-6.80	42.28	0.22	-29.28	0.27
βCD	AMB	22	7.08	0.02	38.17	0.19	-6.80	38.45	0.19	-26.41	0.22
βCD	HFL	21	1.20	0.04	52.88	0.19	-6.80	47.28	0.19	-37.81	0.23
βCD	GGL	10	1.20	0.04	53.18	0.25	-6.80	47.58	0.25	-39.66	0.29
γCD	GACE	17	0.82	0.04	41.09	0.17	-6.80	35.11	0.17	-21.92	0.21
γCD	NACE	Z-17	0.69	0.02	41.84	0.16	-6.80	35.73	0.16	-22.73	0.20
γCD	AMB	22	0.27	0.00	53.73	0.19	-6.80	47.19	0.19	-35.15	0.22
γCD	HFL	21	1.01	0.03	45.89	0.21	-6.80	40.10	0.21	-30.63	0.24
2HP-αCD	GACE	17	2.30	0.06	46.40	0.27	-6.80	41.90	0.27	-28.71	0.29
2HP-αCD	NACE	Z-17	1.79	0.06	43.66	0.32	-6.80	38.65	0.32	-25.65	0.34
2HP-αCD	AMB	22	47.32	0.03	-10.47	0.20	-6.80	30.05	0.20	-18.01	0.23
2HP-αCD	HFL	21	3.55	0.01	45.15	0.19	-6.80	41.90	0.19	-32.43	0.22
2HP-βCD	GAC	6	1.27	0.03	56.67	0.21	-6.80	51.14	0.21	-35.88	0.23
2HP-βCD	GACE	17	0.57	0.02	59.66	0.22	-6.80	53.43	0.22	-40.24	0.25
2HP-βCD	NACE	Z-17	2.65	0.04	59.99	0.26	-6.80	55.84	0.26	-42.84	0.29
2HP-βCD	C13P	24	6.76	0.08	50.12	0.37	-6.80	50.08	0.38	-37.08	0.41
2HP-βCD	AMB	22	9.35	0.02	45.61	0.40	-6.80	48.16	0.40	-36.12	0.42
2HP-βCD	HFL	21	0.90	0.05	62.69	0.25	-6.80	56.79	0.25	-47.32	0.28
2HP-βCD	GGL	10	2.07	0.01	63.78	0.32	-6.80	59.05	0.32	-51.13	0.35
2HP-γCD	GACE	17	0.65	0.03	57.54	0.25	-6.80	51.39	0.25	-38.20	0.28
2HP-γCD	NACE	Z-17	0.45	0.02	57.19	0.25	-6.80	50.84	0.25	-37.84	0.27
2HP-γCD	AMB	22	0.17	0.00	71.89	0.33	-6.80	65.25	0.33	-53.21	0.34
2HP-γCD	HFL	21	1.08	0.03	64.15	0.34	-6.80	58.43	0.34	-48.96	0.36

C. Supplementary Methods

Chemicals

The chemicals used for syntheses, molecular biology and biochemical work were purchased from Carl-Roth (Karlsruhe, DE), VWR (Pennsylvania, US), Sigma-Aldrich/ Merck (St. Louis, US), TCI Chemicals (Tokyo, JP), and Alfa-Aesar (Ward Hill, US). Cyclodextrins were purchased from Wacker (Munich, DE). Pure E-geranyl acetone and Z-geranyl acetone were kindly provided by BASF. Lipase was purchased from Merck/Sigma-Aldrich (Product-Nr. 54326).

Molecular biological kits

The molecular biological kits for DNA-purification (ZymoClean DNA Clean & Concentrator Kit), Agarose Gel-extraction (ZymoClean Gel DNA Recovery Kit) and plasmid isolation (Zyppy™Plasmid Miniprep Kit) were purchased from ZymoResearch (Irvine, US).

Primers, Buffers & Media

Lists of primers, buffers and media used in this work are given in Supplementary Table 3-5.

Nuclear Magnetic Resonance

^{1}H- und ^{13}C-NMR spectra were recorded on a Bruker Avance 500 or 700 Spectrometer at 500.15 or 700.36 MHz for ^{1}H- and 125.76 MHz for ^{13}C. The chemical shifts are referred to tetramethylsilane (=TMS) in ppm set to 0. All substances were dissolved in $CDCl_3$ and recorded at room temperature.

High-resolution mass spectrometry (HRMS)

HRMS data was measured by the University of Stuttgart using an Exactive GC Orbitrap MS (Thermo Fisher Scientific).

Gas chromatography (GC)

GC-MS analyses were performed using an Agilent GC 7820A equipped with a CTC Pal-Sampler and a mass spectrometer MSD 5977B and a HP-5MS capillary column (Agilent, 30 m x 250 µm x 0.25 µm) and helium as carrier gas. Injections (1 µL) were performed in split mode. Relative conversions were calculated directly from GC-MS spectra by integration-quotient of substrates and products. GC-FID analyses were performed using a Shimadzu GC-2010 Plus equipped with an auto injector and DB-5 capillary column (Agilent, 30 m x 250 µm x 0.25 µm) and hydrogen as carrier gas. Chiral GC analyses were performed on a Shimadzu GC-2010 Plus equipped with a CP ChiraSil-Dex CB capillary column (Agilent, 25 m x 250 µm

x 0.25 µm) and HP chiral 20B (Agilent, 30 m x 320 µm x 0.25 µm) and hydrogen as carrier gas. Injections (1 µL) were performed in split mode.

Circular dichroism

The specific optical rotations were measured on a Perkin Elmer Polarimeter 241. Therefore, the substance was dissolved in $CHCl_3$, and the specific rotation was determined with a sodium and a mercury spectral lamp.

Plasmid isolation

Isolation of the plasmid proceeded following to the standard protocol of Zyppy™Plasmid Miniprep Kit by ZymoResearch. For the photometric determination of the plasmid DNA concentration, 2 µL were measured on a Nanodrop 1000 (Agilent, Santa Clara, US) at a wavelength of 260 nm**.**

Site-saturation mutagenesis

The gene encoding for AacSHC, or variants based on these genes were cloned into a pET-22b(+) vector system (Merck, Darmstadt, Germany). SacI and NdeI were used as restriction sites. Cloning followed the standard protocol of New England Biolabs Phusion High Fidelity DNA Polymerase.

Site-saturation libraries were generated by using NNK codons. PCR products were digested with 1 µL DpnI for 2-4 h at 37 °C, purified by agarose gel electrophoresis and ligated into the pET22b(+) vector by Gibson assembly.[23] Afterwards the plasmids were transformed via heat-shock method (see below). Site-saturation was used to generate variant NMZ+I432T in Figure S14.

Plasmid transformation via heat-shock method

Chemically competent cells based on rubidium chloride[24] were produced for the transformation of the plasmid DNA. The transformation was carried out at 4 °C. For site saturation libraries 3 µL (5 µL for single-point mutations) of the purified (crude) PCR product was added to 25 µL XL1-blue competent cells and incubated for 30 min on ice, followed by a heat shock at 42 °C for 105 s with subsequent ice cooling for 3 min. After adding 500 µL of LB medium, the cells were incubated for 40min at 37 ° C and used for inoculation of a 5 mL LB medium (Ampicillin, cend = 100 µg/mL) pre-culture overnight. Single-point variants were plated on petri dishes and grown colonies were picked and grown in 5 mL LB medium. After isolation of the plasmid, sequencing for quality control was performed. Subsequently, transformation into 50 µL BL21(DE3) was performed using the heat shock method. After regeneration 150 µL were streaked out on an agar plate (Ampicillin, c_{end} = 100 µg/mL) and incubated at 37 °C overnight. For quality control the plasmid was isolated from another 150 µL and sent for sequencing. For

site-directed mutants the PCR product was digested with DpnI overnight and afterwards transformed into XL1-blue competent cells. After regeneration 300 µL were streaked out on an agar plate for single clone picking.

Expression of SHC in Erlenmeyer flasks

Expression cultures were inoculated with 5 mL of the pre-culture into 500 mL of T-DAB autoinduction medium (Ampicillin, c_{end} = 100 µg/mL) with lactose as the inductor. The cultures were incubated for 20 h at 37 °C, 180 rpm and harvested afterwards (8000 rpm, 30 min). The resulting pellets were frozen at −80 °C overnight and lyophilized afterwards for storage. Alternatively, the cell pellets were directly used for biotransformations or thermolysis purification.

Expression of SHC libraries in 96-DW plates

Individual colonies were picked from generated agar plates and cultivated in 500 µL LB medium (Ampicillin, c_{end} = 100 µg/ml) for 18-20 h at 37 °C, 800 rpm. Expression cultures were inoculated with 10 µL of the pre-culture into 1 mL of T-DAB autoinduction medium (Ampicillin, c_{end} = 100 µg/mL) with lactose as the inductor. The cultures were incubated for 20 h at 37 °C, 800 rpm and harvested afterwards (4000 x g, 20 min).

Expression in 24 DW-plates

Individual colonies were picked from generated agar plates and cultivated in 2 mL LB medium (Ampicillin, c_{end} = 100 µg/mL) for 18-20 h at 37 °C, 180 rpm. Expression cultures were inoculated with 40 µL of the pre-culture into 4 mL of T-DAB autoinduction medium (Ampicillin, c_{end} = 100 µg/mL) with lactose as the inductor. The cultures were incubated for 20 h at 37 °C, 600 rpm and harvested afterwards (4000 x g, 20 min).

Lyophilization protocol

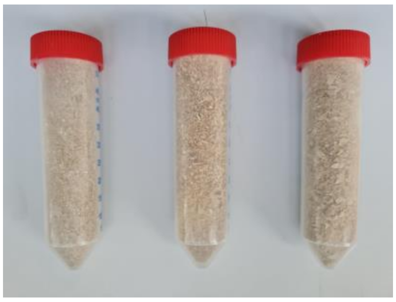

Supplementary Figure 19: Lyophilized E.coli cell powder stored in 50 mL Falcon tubes.

Freshly harvested *E. coli* cells were transferred to petri dishes and frozen at –80°C overnight. On the following day the frozen pellets were quickly transferred to *Christ alpha 2-4 LD plus* lyophilizer and lyophilized at –80 °C at 0.0001 atm for one. The resulting lyophilized whole cells were mortared gently and stored in 50 mL Falcon tubes at room temperature. The enzyme content for each batch was determined using the thermolysis purification protocol.

Thermolysis purification[25,26]

Lyophilized cells (10 mg) were resuspended in 1 mL *Lysis buffer* and incubated for 30 min at 70 °C. The cell suspension was centrifuged (14000 x g, 1 min) and the supernatant was discarded. As the enzyme is membrane-bound, 1 mL *CHAPS buffer* was added to extract it from the cell pellet by shaking at room temperature for 1-2 d, 600 rpm. After subsequent centrifugation (14000 x g, 1 min) the supernatant containing the SHC (*Aac* or *Tel*) was transferred to a new tube followed by SDS-PAGE analysis and determination of enzyme concentration by using the *Nanodrop 1000* (Agilent, Santa Clara, US). Therefore the "Protein A280" mode was chosen with MW = 71439 Da and molar extinction coefficient ε = 185180 as protein specific data for *Aac*SHC and MW = 72668 and ε = 189230 for *Tel*SHC.

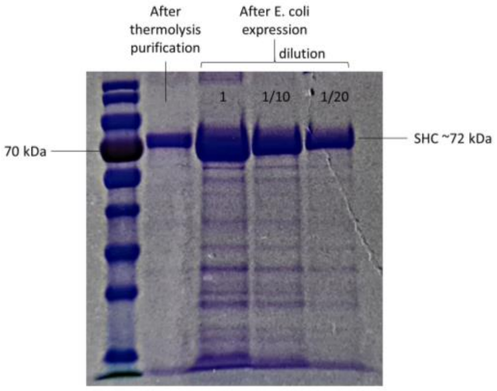

Supplementary Figure 20: SDS-PAGE of the AacSHC expressed by E. coli after and before (including dilutions) using the thermolysis purification.

SDS-PAGE

After expression or purification 15 µL of the cell suspension (10 µL of the enzyme preparation) was mixed with 5 µL (2 µL) SDS loading buffer (Expedeon) and heated to 95 °C for 10 min. Afterwards 10 µl of the preparation was loaded on the pre-prepared SDS-PAGE (Expedeon) and ran for 70 min at 110V. Exemplary SDS-PAGE with four different SHC variants:

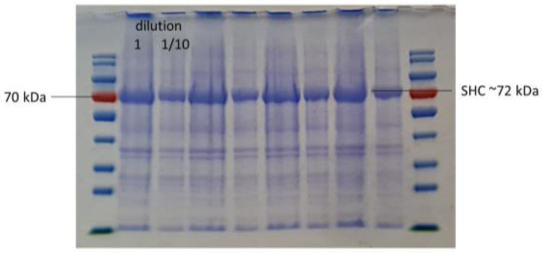

Supplementary Figure 21: SDS-PAGE of various AacSHC variants expressed by E. coli.

Analytical biotransformations in GC screw-cap-vials

10 mg/ml lyophilized cells were resuspended in water, water supplemented with cyclodextrins or buffer or both. 495 µL of the cell suspension were transferred to GC screw-cap-vials and

5 µL of a substrate/DMSO stock (c_{end}, substrate = 1 mM, unless otherwise noted in the reaction conditions) were added to start the reactions. Reactions were shaken at 30 °C or 50 °C for 20 h, depending on the substrate (see Fig 2). Reactions were stopped by adding 600 µL CH:EtOAc (1:1), vortexing and shaking for 5 min. After centrifugation (4000 rpm, 5 min) the organic phase was analyzed via GC-MS equipped with a PAL-Sampler directly from the two-phase system. Quantification was done by GC-FID and dodecane as internal standard.

Screening of *Aac*SHC libraries via GC-MS

Harvested pellets were resuspended in 396 µL H_2O and transferred to another 96-DW plate equipped with 1.2 mL glass inlets. Afterwards 4 µL substrate/DMSO stock solution (substrate c_{end} = 1 mM) was added directly into the cell suspension, the plates were sealed and shaken for 20 h at 30 or 50 °C, 600 rpm. To stop the reaction 600 µL ethyl acetate/o-xylene (1:1) was added and the mixture was inverted for 10 min and incubated for 30 min. The plates were centrifuged (4000 x g, 5 min), sealed using polypropylene-sealings and a GC-MS equipped with a PAL-Sampler was used to inject directly from the organic phase. Quantification (GC conversion) was done directly from the Total Ion Count chromatogram by quotient $AREA_{product}$/ ($AREA_{substrate}$+$AREA_{product}$)*100. In total 90 variants (6 control wells) per plate were screened. Promising variants were rescreened by expression in 24 DW-plates.

Verification of promising hits

Promising candidates from the 96-DW screening were taken for inoculation of a 5 mL LB pre-culture. Afterwards the plasmids were isolated and transformed for single colony picking. The single colonies were expressed in 24 DW-plates and after harvesting, the OD_{600} was set to 10 in *dd*H_2O. 495 µL were transferred to a 2 mL screw cap vial and 1 mM (5 µL) substrate was added as DMSO stock solution (c_{DMSO} = 1%). The reactions were carried out at least in technical duplicates as analytical biotransformations. Reactions were carried out at 50 °C and 600 rpm for 24 h. Reactions were stopped by adding CH:EtOAc (1:1) and inversion of the suspension. After two extractions, the resulting organic phase was measured directly over GC-MS or FID. Quantification was done by dodecane is internal standard.

Determination of turnover frequency and total turnover *in vivo*

For the determination of the whole cell turnover frequency ($mol_{product}/mol_{enzyme}*h^{-1}$) and total turnover ($mol_{product}/mol_{enzyme}$), the SHC content of 10 mg_{CDW} of the lyophilized batch was determined *via* Thermolysis protocol. The concentrations were determined in triplicates and full extraction was followed by SDS-PAGE.

Fluorescence microscopy

Samples for Fluorenscence microscopy were prepared as follows: 1 mg_{CDW} lyophilized and 4.5 mg_{CWW} freshly prepared *E. coli* cells were fixed using 100 µl 4% paraformaldehyde in PBS solution. In order to wash the cells, the mixture was centrifuged (12.000 x g, 30 sec), the supernatant was discarded, and 1 mL of PBS solution was added. The cells were resuspended and centrifuged (12.000 x g, 30 sec) again. The supernatant was discarded, and the resulting pellet was resuspended on 100 µl sterile H_2O. Afterwards 5 µl of the washed samples were struck out on a glass plate (Thermo Scientific) marked with 5 µl DAPI (Sigma-Aldrich) and SYTOX^{TM} 4-64 (Thermo Scientific) and visualized using Fluorescence microscope (ZEISS Axiovert 200 equipped with a AxioCam HRm) with filters: Fs01 365 ± 12 nm and Fs09 470 ± 40 nm. Pictures were edited with ImageJ. The resulting pictures are presented in Fig. 3 and S10.

Docking simulations

Docking studies were performed using YASARA,[6] which uses Autodock and VINA algorithms for the calculation of defined ligand-receptor interaction. Homolog SHC models of *Tel* was generated by RoseTTaFold.[13] *In silico* mutations of SHCs were introduced by changing the specific amino acid in the sequence based, which homology structure was modeled by RoseTTaFold. The most plausible and lowest energy model was used for visualization.

Free energy calculations

Molecular model

Native (αCD, βCD, γCD) and hydroxypropyl-cyclodextrins (2HP-αCD, 2HP-βCD, 2HP-γCD) were modelled with the GROMOS-compatible force field $53A6_{GLYC}$[27], which is based on the GROMOS 53A6 force field[28] and includes modified torsional-angle parameters for hexopyranoses. In the context of pure carbohydrate systems, the force field 53A6 is equivalent to 53A5[28] as well as recent modifications referred to as 54A7[29,30] and 54A8[31] and nearly identical to the set 45A4[32]. The only minor difference between 45A4 and 53A6 force fields was a change in the repulsive coefficient of the Lennard-Jones interactions between atom type OA (used for all sugar oxygen atoms in these two force fields) and nonpolar carbon atoms (intermolecular or intramolecular beyond third covalent neighbors)[33]. Molecular models for all guest molecules except ambroxide were generated manually by transferring parameters from similar molecules in the GROMOS building block library[30]. For ambroxide (**22**) an initial topology was obtained from the automated topology builder[34]. This topology was adapted manually by replacing tailor-made covalent force field parameters by similar ones from the standard types of the 54A8 set. Moreover, the partial charges were adapted to be consistent with the other molecules. GROMOS topologies of all molecules are provided in the Data

Repository of the University of Stuttgart (https://doi.org/10.18419/darus-4135). The simple point charge (SPC) model[35] was used to describe the solvent water. The membrane was not modelled explicitly but instead mimicked by bulk cyclohexane based on the good agreement between calculated free energies for transferring amino acid side chains from water into the center of a lipid bilayer and the experimental water to cyclohexane transfer free energy.[36] Previous work using the force fields mentioned above indicated good agreement with experimentally determined structural properties for the native cyclodextrins[37] while the binding free energy for different organic molecules including alcohols, ketones and ethers were overestimated by 5 to 10 kJ/mol.[38–40] However, in the present work the difference between two free energies for transferring a guest molecule from water into a hydrophobic environment is relevant. This difference benefits from error compensation because also the transfer free energy of the guest molecule from water into cyclohexane is likely to be overestimated by a similar amount as indicated from comparison between simulated and reported thermophysical properties for neryl acetone (***Z*-17**) (enthalpy of vaporization, vapor pressure and aqueous solubility).

Simulation Details

All simulations were conducted with the GROMACS 2016.4 program[41] patched to the free-energy library PLUMED 2.4.2[42] for restraints definition. Each system was solvated with ~1450 water molecules in an orthorhombic box of approximate dimensions 3.6 x 3.6 x 3.6 nm^3. Production simulations were run at isothermal-isobaric conditions at 300 K and ambient pressure, with temperature control using a Nosé-Hoover thermostat[43,44] (friction constant $\tau_T = 1.0\ \mathrm{ps}^{-1}$) and a Parrinello-Rahman barostat[45] (coupling constant $\tau_P = 2.0\ \mathrm{ps}^{-1}$) for pressure control. A Verlet-buffered neighbor list[46] which was updated every 40 steps, was applied for the treatment of short-range electrostatic and van der Waals interactions with potentials shifted to zero at 1.4 nm. The latter were modeled by the Lennard-Jones potential. Analytic dispersion corrections were applied for energy and pressure calculation. Long-range electrostatic interactions were treated with the smooth particle-mesh Ewald (PME) method[47] using a real-space cut-off of 1.4 nm with a cubic splines interpolation scheme and a grid spacing of 0.12 nm. The center of mass (COM) translation of the computational box was removed every 1000 steps. All bond lengths as well as water bond angles were constrained using the LINCS algorithm.[48]

Double Decoupling

Following the double decoupling methodology (DDM), the standard binding free energy for a guest bound to a host molecule (here in the form of a solvated 1:1 complex) is calculated as:[49]

$$\Delta G^0_{\mathrm{bind}} = \Delta G^{\mathrm{B}}_{0*\to 1*} - \Delta G^{\mathrm{H}}_{0\to 3} \tag{1}$$

with

$$\Delta G^{\mathrm{H}}_{0\to 3} = \Delta G^{\mathrm{H}}_{0\to 1} + \Delta G^{\mathrm{H}}_{1\to 2} + \Delta G^{\mathrm{H}}_{2\to 3} \tag{2}$$

(see Fig. S18 (A)). $\Delta \mathrm{G}^{0}_{\mathrm{bind}}$ is equivalent to the transfer free energy from bulk water to the cyclodextrin (CD)-based host ($\Delta G_{\mathrm{H2O}\to\mathrm{CD}}$). $\Delta G^{\mathrm{H}}_{0\to 1}$ corresponds to the free energy contribution for the application of a distance restraint between the COM of the fully interacting and bound guest and the COM of cyclodextrin in order to prevent the guest from leaving the host's binding pocket once it is decoupled. This distance restraint (harmonic flat-bottom potential with force constant $K_r = 500\ \mathrm{kJ\ mol^{-1} nm^{-2}}$ and threshold parameter $r_0 = 0.2\ \mathrm{nm}$) was activated prior to the decoupling of the guest by gradually increasing the value of K_r from 0 (unrestrained bound guest) to the final value within a sequence of 8 discrete steps with 20 ns per step. The free energy contribution for removing this distance restraint again from the decoupled i.e. non-interacting guest is given by $\Delta G^{\mathrm{H}}_{2\to 3}$. This purely entropic contribution can be calculated analytically and accounts for the transfer of the decoupled guest from the effective volume V_{eff} to the standard state volume $V^0 = 1.661\ \mathrm{nm}^3$ and therefore represents the standard state correction for the calculation of $\Delta G^{0}_{\mathrm{bind}}$:[50]

$$\Delta G^{\mathrm{H}}_{2\to 3} = -RT \ln\left(\frac{V^0}{V_{\mathrm{eff}}}\right) \tag{3}$$

The effective volume V_{eff} accessible to the decoupled restrained guest is given by the integral:

$$V_{\mathrm{eff}} = \frac{4}{3}\pi r_0^3 + \int_{r_0}^{\infty} \exp\left\{-\frac{K_r}{2\,RT}(r - r_0)^2\right\} 4\pi\, r^2\, \mathrm{d}r \tag{4}$$

where the integration variable r denotes the radial host-guest-COM-COM distance. Numerical evaluation of the integral using the parameters given above, yields $\Delta G^{\mathrm{H}}_{2\to 3} \approx -6.80\ \mathrm{kJ\ mol^{-1}}$. Results for the decoupling free energies $\Delta G^{\mathrm{B}}_{0*\to 1*}$ and $\Delta G^{\mathrm{H}}_{0\to 3}$ for all simulated systems are reported in supplementary figure 18 (B) and supplementary tables 6 and 7.

For bulk simulations, a single guest molecule immersed in a bath of solvent (water or cyclohexane) molecules was considered, while for the host-guest complexes, a single guest bound to a single solvated host molecule (1:1 complex) was simulated. The scaling of the non-bonded interactions between the guest and its environment was controlled via a coupling parameter λ, such that λ = 0 and λ = 1 represents the fully interacting and fully decoupled guest, respectively, while retaining the intramolecular interactions. The decoupling was conducted in a sequence of 20 discrete steps, using simulation times of 20 ns per λ-state. In the applied perturbation scheme, electrostatic interactions were deactivated first within 5 steps, followed by the deactivation of the Lennard-Jones interactions. To avoid numerical problems close to the end states, soft-core (sc) potentials were used with parameters $\alpha_{sc} = 0.5$, $\sigma_{sc} = 0.3$ and a power for the soft-core scaling function of $p_{sc} = 1$.[41] For enhanced sampling, including

the possibility of sampling different orientations of the guest inside the binding pose, Hamiltonian replica exchange (HRE)[51,52] was applied, with attempted exchanges of the Hamiltonians of neighboring λ-states every 1000 steps. All free energy changes (decoupling, activation of distance restraint) were estimated from the sampled potential energy differences between all λ-states using the MBAR estimator[53] as implemented in a freely available Python program.[54] For selected systems, we also conducted complementary umbrella sampling (US) simulations combined with HRE among adjacent umbrella windows (denoted as HRE-US), and estimated $\Delta G^{0}_{\mathrm{bind}}$ from the resulting one-dimensional free energy profile as done previously.[55] In all cases, results from HRE-US and DDM were found to be in good agreement (data not shown).

D. Preparative scale biotransformations using lyophilized *E. coli* cells harboring the *Aac*SHC variants.

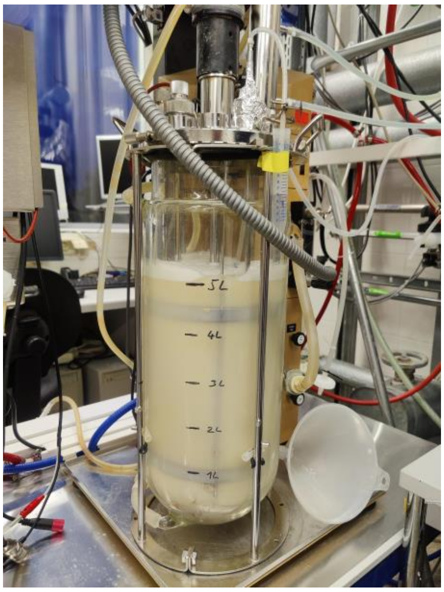

Supplementary Figure 22: Biotransformation carried in a 5 L reactor. The process only involved heating and stirring.

Reactor conditions: To an Infors HT 5L equipped with Infors AG CH-4103 were added 50 g_{CDW}, 5 L *dd*H_2O, 25 g substrate 17 and 70 g of 2-hydroxypropyl-β-cyclodextrin. The reactor was warmed to 30 °C and the reaction was stirred at 100 rpm for 84 days. 100 µL samples were taken and extracted to monitor reaction progress. For reaction progress see Fig. 3 in the main text. The reaction was extracted with ethyl acetate by overlaying and slowly stirring the cells/organic solvent mixture, to avoid phase mixture. The organic phase was dried over Mg_2SO_4 and purified via silica flash column (10:1, cyclohexane:ethyl acetate). Product 18 was obtained as colorless oil 22.4 g (115 mmol, 89.6%).

General procedure of preparative scale biotransformation.

After the product selective *Aac*SHC variant was identified, it was produced in *E. coli* in large scale (6-12 Erlenmeyer flasks) following the protocol described above. The resulting cell pellets were lyophilized afterwards, and the enzyme concentration was determined via the thermolysis protocol (see above). The reaction mixture contained a given amount of cells, the given amount of substrate, and optionally a given amount of 2-HPCD or citrate buffer. The reactions were performed in closed 100 mL or 1L Schott-flasks in an INFORS-HT incubator at

180 rpm for the given time. After the reaction was finished (GC monitoring) the cell suspension was overlayed with 50 mL or 500 mL cyclohexane:ethyl acetate (1:1) and stirred slowly to avoid mixing of the phases for 24 h. The organic phase was dried over $MgSO_4$, reduced, and the crude products were analyzed via GC prior to column chromatography (20:1 cyclohexane:ethyl acetate). GC yield is given below the reaction arrows and was determined by $[area_{product}]:[area_{substrate}+area_{product}]$. **A comprehensive summary of the reaction conditions and yields can be found in Supplementary Table 2**. Asterisks mean no determination via chiral GC but suggesting the chirality due to the inherent ability of the SHC to produce a single enantiomer facilitated by the strongly confined active site.[56] Inconsistencies between preparative biotransformation yields stem from the availability of the linear precursors.

6 with G600R to cyclogeranyl acetate hydrate (7)

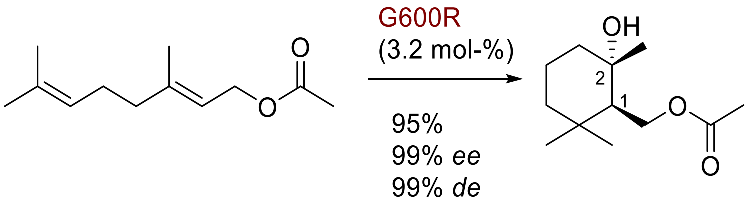

90 % *E*-isomer
6 **7**

Product 7 was obtained as pale-yellow oil. For details, please see Table S2. $C_{12}H_{22}O_3$ **^{1}H-NMR (CDCl$_3$, 500 MHz)**: δ (ppm) 0.87 (s, 3H), 1.02 (s, 3H), 1.22 (s, 3H), 1.36-1.80 (m, 7H), 2.06 (s, 3H), 4.32-4.34 (d, J = 2.9 Hz, 2H). **^{13}C-NMR (CDCl$_3$, 125 MHz):** δ (ppm) 20.0 (1C), 21.3 (1C), 24.0 (1C), 26.9 (1C), 32.7 (1C), 34.1 (1C), 41.8 (1C), 42.4 (1C), 54.8 (1C), 63.5 (1C), 72.7 (1C), 171.0 (1C). The data is consistent with the literature.[17]

17 with variant G600M to *trans*-chromene (18)

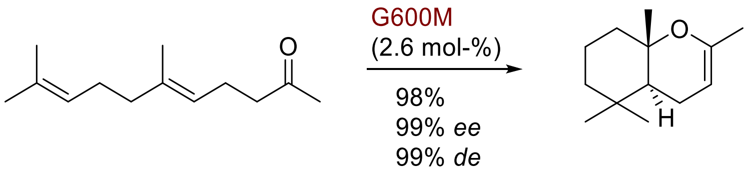

99% *E*-isomer
17 **18**

Product 18 was obtained as a colorless oil. $C_{13}H_{22}O$ **^{1}H-NMR (CDCl$_3$, 500 MHz):** δ (ppm) 0.81 (s, 3H), 0.91 (s, 3H) 1.17 (s, 3H), 1.21-1.29 (m, 1H), 1.40-1.60 (m, 5H), 1.68 (s, 3H), 1.72-1.94 (m, 3H), 4.40-4.50 (m, 1H). **^{13}C-NMR (CDCl$_3$, 125 MHz):** δ (ppm) 19.1 (1C), 19.2 (1C), 19.8 (1C), 20.5 (1C), 20.8 (1C) 30.3 (1C), 32.3 (1C), 40.0 (1C), 41.7 (1C), 48. 4 (1C), 76.5 (1C),

95.0 (1C), 148.0 (1C). The data is consistent with the literature.[57]

Z-17 with variant G600M to *cis*-chromene (23)

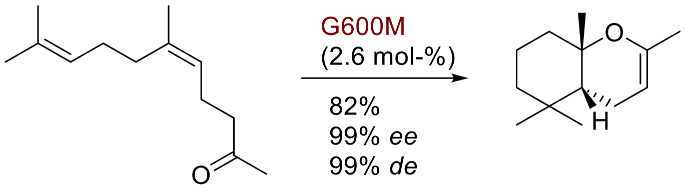

99% *Z*-isomer
Z-17 **23**

Product 23 was obtained as a colorless oil. $C_{13}H_{22}O$ **^{1}H-NMR** (**$CDCl_3$**, **500 MHz**): δ (ppm) 0.85 (s, 3H), 0.87 (s, 3H) 1.19 (s, 3H), 1.22-1.42 (m, 6H), 1.57 (s, 3H), 1.66-2.23 (m, 3H), 4.36-4.39 (d, J = 2.6 Hz, 1H). **^{13}C-NMR** (**$CDCl_3$**, **125 MHz**): δ (ppm) 18.1 (1C), 19.8 (1C), 20.5 (1C), 21.2 (1C), 26.5 (1C) 32.5 (1C), 33.7 (1C), 39.6 (1C), 42.0 (1C), 44.0 (1C), 74.7 (1C), 94.5 (1C), 148.7 (1C).

13 with variant F601D F605L to sclareoloxide (14)

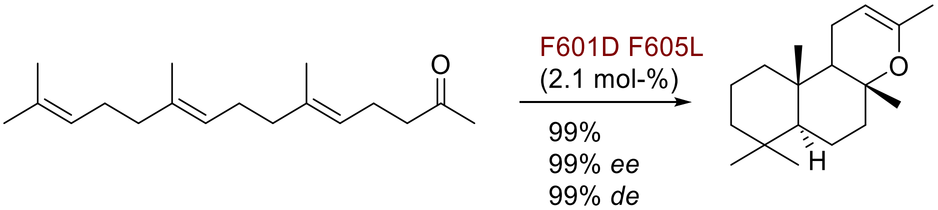

99% *E*-isomer
13 **14**

Product 14 was obtained as a yellow oil that solidified upon cooling. $C_{18}H_{30}O$ **^{1}H-NMR ($CDCl_3$, 500 MHz):** δ (ppm) 0.81 (s, 3H), 0.813 (s, 3H) 0.88 (s, 3H), 1.15 (s, 3H), 1.24-1.51 (m, 8H), 1.56-1.63 (m, 3H), 1.68 (s, 3H), 1.69-1.87 (m, 2H), 1.69-19.60 (dt, J_d = 5.4, J_t = 3.0, 1H), 4.40 (dt, J_d = 1.9 Hz, J_t = 1.0 Hz, 1H) **^{13}C-NMR ($CDCl_3$, 125 MHz):** δ (ppm) 15.0 (1C), 18.3 (1C), 18.6 (1C), 19.8 (1C), 20.1 (1C), 20.5 (1C), 21.6 (1C), 33.2 (1C), 33.5 (1C), 36.7 (1C), 39.3 (1C), 41.1 (1C), 41.9 (1C), 52.4 (1C), 56.2 (1C), 76.2 (1C), 94.6 (1C), 147.9 (1C) $[\alpha]_D^{20}$ = 6.4 (c = 0.78 in $CHCl_3$); Lit. = 4.9 (c = 1.3 in $CHCl_3$). The data is consistent with the literature.[58]

Z-17 with variant G600T L607A Y420F A306W D432T to γ-dihydroionone (24)

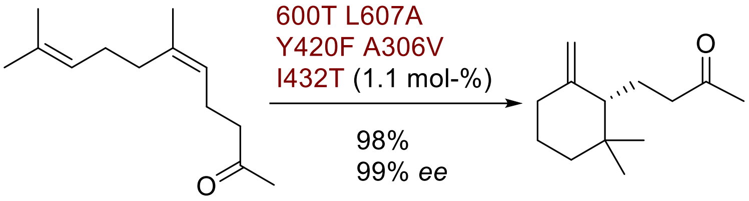

99% Z-isomer
Z-17 **24**

Product 24 was obtained as a colorless oil. $C_{13}H_{22}O$ **^{1}H-NMR ($CDCl_3$, 500 MHz):** δ (ppm) 0.87 (s, 3H), 0.92 (s, 3H) 1.10-1.30 (m, 2H), 1.42-1.62 (m, 3H), 1.66-1.70 (m, 1H), 1.76-1.83 (m, 1H), 1.97-2.04 (m, 2H), 2.11 (s, 3H), 2.22-2.45 (m, 2H), 4.50-4.51 (d, J = 1.03 Hz, 1H), 4.75-4.77 (m, 1H). **^{13}C-NMR ($CDCl_3$, 125 MHz):** δ (ppm) 20.3 (1C), 22.6 (1C), 23.5 (1C), 26.5 (1C), 28.3 (1C), 30.2 (1C), 32.0 (1C), 34.8 (1C), 42.4 (1C), 53.4 (1C), 109.5 (1C), 149.1 (1C), 209.5 (1C). The data is consistent with the literature.[59] Stereoselectivity was determined in ref. [8].

8 with variant A306W to drimane diol (9)

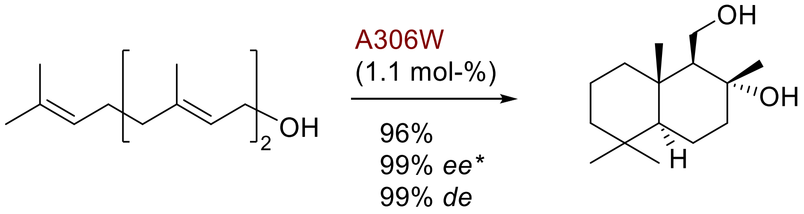

85% E,E-isomer
8 **9**

Product 9 was obtained as a white solid. $C_{15}H_{28}O_2$ **^{1}H-NMR** (**$CDCl_3$**, **500 MHz**): δ (ppm) 0.79 (s, 6H), 0.88 (s, 3H), 0.95-0.98 (m, 1H), 1.07-1.29 (m, 4H), 1.35 (s, 3H), 1.42-1.57 (m, 4H), 1.63-1.67 (m, 2H), 1.87-1.90 (tt, J_t = 6.1 Hz, 1H), 3.91-3.93 (m, 2H). **^{13}C-NMR ($CDCl_3$, 125 MHz):** δ (ppm) 16.1 (1C), 18.6 (1C), 20.2 (1C), 21.6 (1C), 24.3 (1C), 33.3 (1C), 33.6 (1C), 37.5 (1C), 40.0 (1C), 41.7 (1C), 44.5 (1C), 55.9 (1C), 60.5 (1C), 61.1 (1C), 75.1 (1C). The data is consistent with the literature.[60] $[\alpha]_D^{20}$ = +10.2 (c = 0.98 in $CHCl_3$); Lit. = 5.5 (c = 0.5 in $CHCl_3$).[61]
*assumed due to enantioselective cyclization of *E*,*E*-farnesol 8 to drimenol (see chromatograms)

8 with variant Y420F G600L L36V to (+)-drimane diol (S1-9)

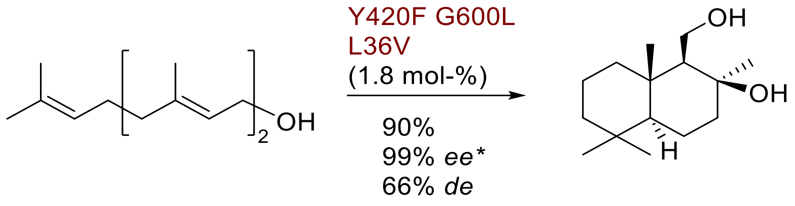

85% *E*,*E*-isomer
8

S1-9

Product S1-9 was obtained as a white solid. $C_{15}H_{28}O_2$ ^{1}**H-NMR** (**CDCl$_3$**, **700 MHz**): δ (ppm) 0.86 (s, 3H), 0.87 (s, 3H), 1.09-1.16 (td, J_t = 13 Hz, J_d = 1.3 Hz, 1H), 1.23 (s, 3H), 1.34 (s, 3H), 1.35 (s, 3H), 1.39-1.76 (m, 4H), 1.63-1.67 (m, 1H), 1.86-1.92 (m, 1H), 2.33-2.36 (t, J = 7.5 Hz, 2H), 4.05-4.14 (m, 2H). ^{13}**C-NMR (CDCl$_3$, 176 MHz):** δ (ppm) 14.1 (1C), 17.0 (1C), 18.4 (1C), 21.7 (1C), 22.7 (1C), 24.7 (1C), 33.4 (1C), 38.4 (1C), 40.0 (1C), 41.9 (1C), 42.9 (1C), 55.9 (1C), 58.3 (1C), 60.1 (1C), 74.2 (1C). The data is consistent with the literature.[62] $[\alpha]_D^{20}$ = +16.4 (c = 0.83 in $CHCl_3$); Lit. = 12 (c = 1.05 in $CHCl_3$).[62]

10 with variant G600N to labdane (12)

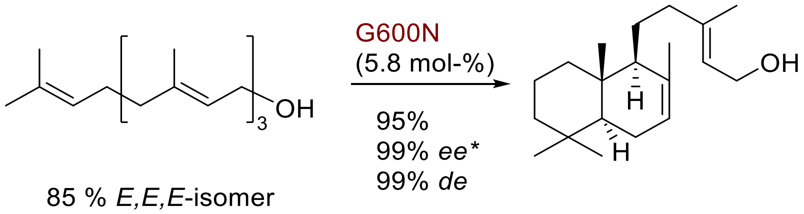

85 % *E,E,E*-isomer
10

12

Product 12 was obtained as a colorless highly viscous oil. $C_{20}H_{34}O$ ^{1}**H-NMR** (**CDCl$_3$**, **400 MHz**): δ (ppm) 0.76 (s, 3H), 0.82 (s, 3H), 0.84 (s, 3H), 0.80−1.09 (m, 2H), 1.56 (s, 3H), 1.69 (s, 3H), 1.02−2.01 (m, 11H), 2.13−2.31 (m, 1H), 4.09−4.20 (m, 2H), 5.10-5.13 (m, 1H), 5.40−5.43 (t, *J* = 7.2 Hz, 1H). ^{13}**C-NMR (CDCl$_3$, 100 MHz):** δ (ppm) 12.5 (1C), 15.9 (1C), 17.7 (1C), 20.1 (1C), 22.2 (1C), 23.7 (1C), 25.3 (1C), 30.9 (1C), 33.9 (1C), 37 (1C), 39.1 (1C), 42.0 (1C), 42.3 (1C), 50.1 (1C), 53.4 (1C), 59.4 (1C), 120.2 (1C), 121.3 (1C), 135.2 (1C), 141.4 (1C). The data is consistent with the literature.[63] $[\alpha]_D^{20}$ = +9.0 (c = 0.64 in $CHCl_3$); Lit. = 5.0 (c = 0.5 in $CHCl_3$).[63]

10 with variant F605W to *ent*-isocopalol (11)

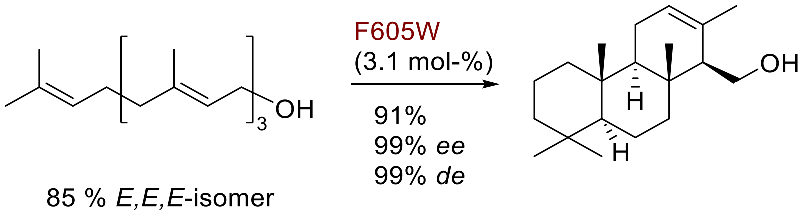

85 % *E,E,E*-isomer
10

11

Product 11 was obtained as pale green solid. $C_{20}H_{34}O$ **^{1}H-NMR** (**$CDCl_3$**, **400 MHz**): δ (ppm) 0.81 (s, 3H), 0.83 (s, 3H), 0.86 (s, 3H), 0.88 (s, 3H), 1.1-1.4 (m, 9H), 1.78 (m, 3H), 1.84-2.07 (m, 6H), 3.71-3.74 (dd, *J* = 6.3 Hz, 1H), 3.84-3.87 (dd, *J*= 6.3 Hz, 1H), 5.5-5.51 (m, 1H). **^{13}C-NMR ($CDCl_3$, 125 MHz):** δ (ppm) 15.8 (1C), 15.8 (1C), 18.5 (1C), 18.8 (1C), 21.7 (1C), 21.8 (1C), 22.6 (1C), 33.1 (1C), 33.4 (1C), 36.2 (1C), 37.2 (1C), 39.9 (1C), 41.5 (1C), 41.9 (1C), 54.8 (1C), 56.3 (1C), 57.9 (1C), 60.9 (1C), 123.9 (1C), 132.6 (1C). The data is consistent with the literature.[64] $[\alpha]_D^{20}$ = –6.0 (c = 0.55 in $CHCl_3$); Lit. = –10.1 (c = 0.42 in $CHCl_3$).[65]

15 with variant F601D F605L to sclareol oxide acetate (16)

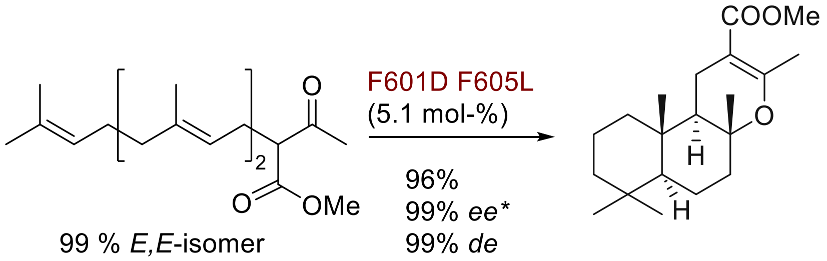

Product 16 was obtained as pale colorless wax. $C_{20}H_{32}O_3$ **^{1}H-NMR** (**$CDCl_3$**, **500 MHz**): δ (ppm) 0.82 (s, 3H), 0.84 (s, 3H) 0.88 (s, 3H), 0.91-0.98 (m, 1H), 1.13 (s, 3H), 1.25-1.39 (m, 4H), 1.50-1.74 (m, 6H), 1.91-2.00 (m, 2H), 2.19 (s, 3H), 2.26-2.32 (dd, *J* = 7.1 Hz, 1H), 3.69 (s, 3H). **^{13}C-NMR ($CDCl_3$, 125 MHz):** δ (ppm) 14.9 (1C), 18.5 (1C), 19.4 (1C), 19.6 (1C), 20.4 (1C), 20.6 (1C), 21.5 (1C), 33.2 (1C), 33.4 (1C), 36.8 (1C), 39.2 (1C), 40.6 (1C), 41.9 (1C), 50.9 (1C), 51.6 (1C), 56.1 (1C), 78.3 (1C), 100.0 (1C), 163.1 (1C), 169.3 (1C). $[\alpha]_D^{20}$ = 7.3 (c = 0.63 in $CHCl_3$). HRMS (EI): calcd. for $C_{20}H_{32}O_3$: 320.2401; found: 320.2409. *assumed due to enantioselective cyclization of *E*,*E*-farnesyl acetone 13 to sclareoloxide 14 (see chromatograms)

E. Chemical synthesis

Synthesis of *E*,*E*-keto ester (15)

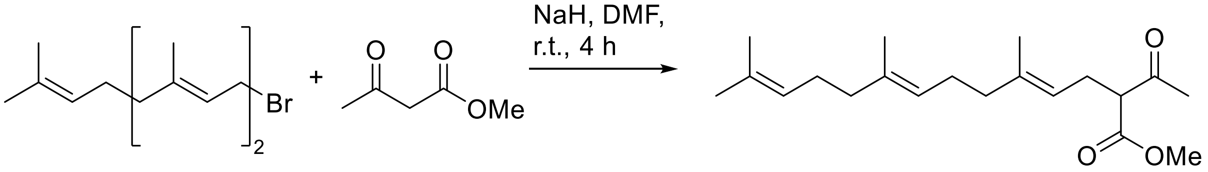

The reaction was carried out under nitrogen atmosphere. A 60 % dispersion of NaH in mineral oil (210.0 mg, 5.25 mmol) was washed with hexane (3x1mL) and suspended in DMF (5mL).

methyl acetoacetate (580 µL, 5.40 mmol was added dropwise. After 30 min, the resulting solution was treated with *E*,*E*-farnesyl bromide (500 mg, 1.75 mmol) and stirred for 4 h. The reaction mixture was quenched with 5 mL of saturated $NaHCO_3$, extracted with EtOAc (3 x 10 mL), and washed with H_2O (4 x 5 mL). The combined organic layers were dried over $MgSO_4$ and concentrated. Column chromatography (CH:EtOAc, 1:20) afforded a colorless oil of 15 (435 mg, 1.36 mmol, 78 %).

$C_{20}H_{32}O_3$ **^{1}H-NMR ($CDCl_3$, 500 MHz):** δ (ppm) 1.58 (s, 3H), 1.60 (s, 3H), 1.62 (s, 3H), 1.67 (s, 3H), 1.94-1.99 (m, 4H), 2.03-2.07 (m, 4H), 2.22 (s, 3H), 2.54-2.57 (t, *J* = 7.5 Hz, 2H), 3.44-3.47 (t, *J* = 7.7 Hz, 1H), 3.72 (s, 3H), 5.02-5.08 (m, 3H). **^{13}C-NMR ($CDCl_3$, 125 MHz):** δ (ppm) 16.0 (1C), 16.1 (1C), 17.7 (1C), 25.7 (1C), 26.5 (1C), 26.7 (1C), 26.9 (1C), 29.2 (1C), 39.68 (1C), 39.71 (1C), 52.3 (1C), 59.6 (1C), 119.5 (1C), 123.8 (1C), 124.3 (1C), 131.3 (1C), 135.2 (1C), 138.6 (1C), 170.1 (1C), 203.1 (1C). The data is consistent with the literature.[22]

Synthesis of ketone (P-1)

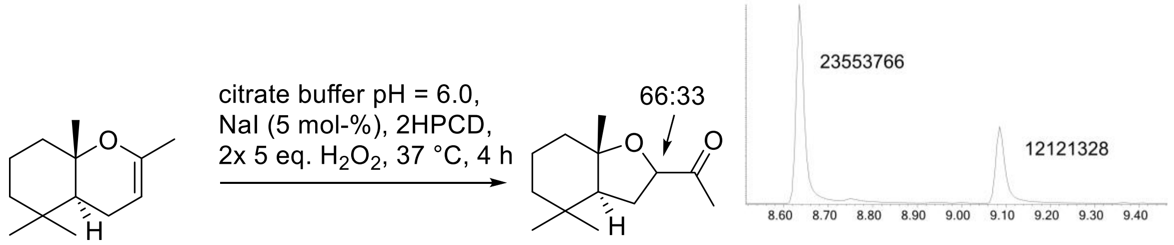

To a 10 mL glass vessel were added 5 mL buffer solution containing 1 mM NaI and 20 mM 2HPCD, was added 21.8 µL (19.4 mg, 0.10 mmol) *trans*-hexahydrochromene 18 and 39.0 µL of 30% H_2O_2 in H_2O (0.50 mmol, 5 eq). The reaction mixture was shaken for 2 h at 37 °C before another portion of 39.0 µL of 30% H_2O_2 was added. After reaction completion determined via GC-MS (4 h total reaction time), the reaction was quenched with sat. $Na_2S_2O_3$ and extracted with ethyl acetate. The organic phase was dried over Mg_2SO_4 and reduced under vacuo to afford a 3:2 diastereoisomeric mixture of P-1 as a colorless oil (20.4 mg, 0.097 mmol, 97% crude yield). Reactions were monitored by taking 10 µL samples that were quenched with 10 µL sat. $Na_2S_2O_3$, extracted with ethyl acetate and measured via GC-MS.

$C_{13}H_{22}O_2$ **^{1}H-NMR ($CDCl_3$, 500 MHz) major diastereoisomer:** δ (ppm) 0.81 (s, 3H), 0.85 (s, 3H) 0.93 (s, 3H), 1.15 (s, 3H), 1.30-1.34 (m, 1H), 1.43-1.46 (m, 2H), 1.66-1.73 (m, 2H), 1.92-2.14 (m, 4H), 2.21 (s, 3H), 4.33-4.37 (dd, J_d = 5.3 Hz, J_d = 1.7 Hz, 1H). **^{13}C-NMR ($CDCl_3$, 125 MHz):** δ (ppm) 19.8 (1C), 20.4 (1C), 21.1 (1C), 26.8 (1C), 28.0 (1C) 32.7 (1C), 33.2 (1C), 38.6 (1C), 40.8 (1C), 55.7 (1C), 80.1 (1C), 82.6 (1C), 211.1 (1C).

Synthesis of ketone (P-2)

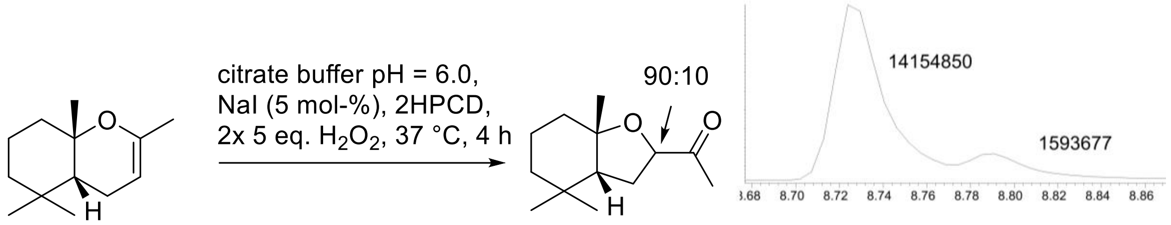

To a 10 mL glass vessel were added 5 mL buffer solution containing 1 mM NaI and 20 mM 2HPCD, was added 21.8 µL (19.4 mg, 0.10 mmol) *cis*-hexahydrochromene 23 and 39.0 µL of 30% H_2O_2 in H_2O (0.05 mmol, 5 eq). The reaction mixture was shaken for 2 h at 37 °C before another portion of 39.0 µL of 30% H_2O_2 was added. After reaction completion determined via GC-MS (4 h total reaction time), the reaction was quenched with sat. $Na_2S_2O_3$ and extracted with ethyl acetate. The organic phase was dried over Mg_2SO_4 and reduced under vacuo to afford a 9:1 diastereoisomeric mixture of P-2 as a colorless oil (16.4 mg, 0.09 mol, 90% crude yield).

$C_{13}H_{22}O_2$ **^{1}H-NMR ($CDCl_3$, 700 MHz) major diastereoisomer:** δ (ppm) 0.90 (s, 3H), 1.01 (s, 3H) 1.17-1.38 (m, 4H), 1.42 (s, 3H), 1.44-1.55 (m, 5H), 2.06-2.09 (m, 1H), 2.22 (s, 3H), 4.22-4.25 (dd, J_d = 5.1 Hz, J_d = 1.4 Hz, 1H). **^{13}C-NMR ($CDCl_3$, 176 MHz):** δ (ppm) 19.9 (1C), 26.5 (1C), 26.5 (1C), 27.3 (1C), 28.3 (2C) 30.6 (1C), 32.3 (1C), 32.9 (1C), 33.9 (1C), 34.0 (1C), 53.0 (1C), 80.1 (1C), 83.5 (1C), 212.9 (1C).

Synthesis of ketone (P-3)

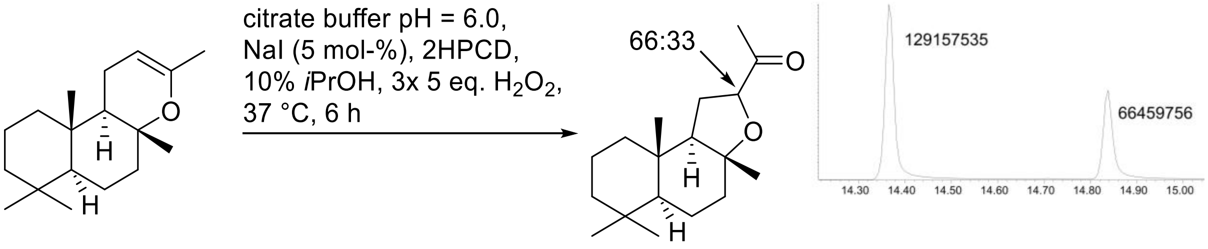

To a 10 mL glass vessel were added 5 mL buffer solution containing 1 mM NaI and 20 mM 2HPCD and 10% isopropanol, was added 26.2 mg (0.10 mmol) sclareoloxide 14 and 39.0 µL of 30% H_2O_2 in H_2O (0.5 mmol, 5 eq). The reaction mixture was shaken for 2 h at 37 °C before another portion of 39.0 µL of 30% H_2O_2 was added. This step was repeated. After reaction completion determined via GC-MS (6 h of total reaction time), the reaction was quenched with sat. $Na_2S_2O_3$ and extracted with ethyl acetate. The organic phase was dried over Mg_2SO_4 and reduced under vacuo to afford a 3:2 diastereoisomeric mixture of P-3 as a colorless oil (25.3 mg, 0.091 mmol, 91% crude yield).

$C_{18}H_{30}O_2$ Characteristic signals of the major diastereoisomer. $C_{18}H_{30}O_2$ **^{1}H-NMR ($CDCl_3$, 500 MHz):** δ (ppm) 2.21 (s, 3H) and 4.37-4.39 (dd, J_d = 5.15 Hz, J_d = 1.6 Hz, 1H). The data is consistent with the literature.[16]

Synthesis of trans-tetrahydroactinidiolide (1) via P-1

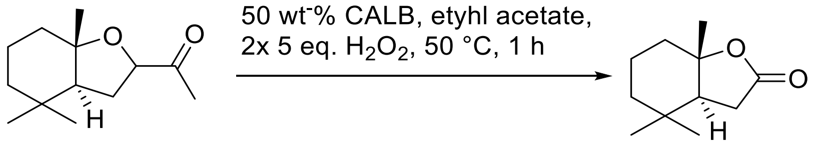

To a 1 mL glass vial were added 500 µL ethyl acetate, 5.9 µL P-1 (5.3 mg, 0.025 mmol), 2.5 mg CALB and 9.7 µL of 30% H_2O_2 in H_2O (0.13 mmol, 5 eq.) sequentially and the reaction mixture was shaken for 30 min at 50 °C. Afterwards another 9.7 µL of 30% H_2O_2 were added. After reaction completion determined via GC-MS (1 h total reaction time), the reaction was quenched with sat. $Na_2S_2O_3$ and extracted with ethyl acetate. The organic phase was dried over Mg_2SO_4 and reduced under vacuo to afford trans-tetrahydroactinidiolide 1 as a white solid (3.2 mg, 0.018 mol, 70%). Reactions were monitored by taking 10 µL samples that were quenched with 10 µL sat. $Na_2S_2O_3$, extracted with ethyl acetate and measured via GC-MS.

$C_{11}H_{18}O_2$ **^{1}H-NMR ($CDCl_3$, 500 MHz):** δ (ppm) 0.93 (s, 3H), 0.96 (s, 3H) 1.34 (s, 3H), 1.53-1.81 (m, 5H), 1.96-2.03 (m, 2H), 2.26-2.48 (m, 2H). **^{13}C-NMR ($CDCl_3$, 125 MHz):** δ (ppm) 19.8 (1C), 20.5 (1C), 20.7 (1C), 29.2 (1C) 32.3 (1C), 32.7 (1C), 37.2 (1C), 40.1 (1C), 55.6 (1C), 86.5 (1C), 178.5 (1C). The data is consistent with the literature.[66]

Synthesis of sclareolide (26) via Lipase/Iodine tandem protocol

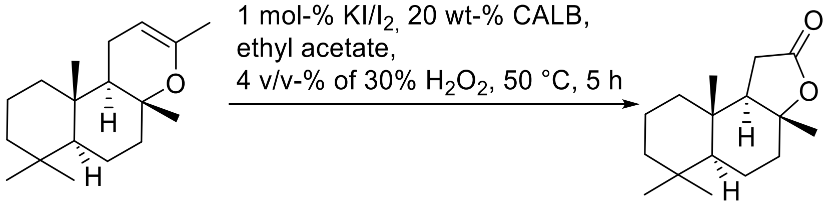

Lugol's iodine (KI/I_2) was prepared by dissolving 1 g of I_2 and 2 g of KI in 300 mL *dd*H_2O (53 mM of which 47 mM is 'iodide' catalyst. To a 10 mL glass vial was added 5 mL ethyl acetate, 12 mg Lipase, 100 µL lugol's iodine (1.0 mol-% iodide) and 66 mg sclareoloxide 14 (0.25 mmol, 50 mM). To this was added 97 µL of 30% H_2O_2 in H_2O (1.25 mmol, 5 eq.) and the reaction was shaken at 50 °C for 3 h. After this time another 97 µL of 30% H_2O_2 in H_2O (1.25 mmol) and the reaction was shaken at 50 °C for another 2 h. After reaction completion determined via GC-MS (5 h total reaction time), the reaction was quenched with sat. $Na_2S_2O_3$ and extracted with ethyl acetate. The organic phase was dried over Mg_2SO_4 and reduced under vacuo to afford

sclareolide 26 as a white solid (44.0 mg, 0.18 mmol, 70%). Reaction time can be shortened to 3 h by adding 50 wt-% CALB.

$C_{16}H_{26}O_2$ **^{1}H-NMR ($CDCl_3$, 700 MHz):** δ (ppm) 0.84 (s, 3H), 0.89 (s, 3H), 0.91 (s, 3H), 1.05-1.07 (m, 2H), 1.19-1.24 (m, 2H), 1.33 (s, 3H), 1.35-1.48 (m, 4H), 1.63-1.74 (m, 2H), 1.85-1.90 (m, 1H), 1.94-2.00 (dd, J_d = 7.3 Hz, J_d = 3.3 Hz, 1H), 2.06-2.09 (m, 1H), 2.21-2.25 (dd, J_d = 7.5 Hz, J_d = 3.4 Hz, 1H), 2.38-2.43 (m, 1H). **^{13}C-NMR ($CDCl_3$, 176 MHz):** δ (ppm) 15.1 (1C), 18.1 (1C), 20.5 (1C), 20.9 (1C), 21.6 (1C), 28.7 (1C), 33.1 (1C), 33.2 (1C), 36.0 (1C), 38.7 (1C), 39.5 (1C), 42.2 (1C), 56.6 (1C), 59.1 (1C), 86.4 (1C), 176.9 (1C). The data is consistent with the literature.[67]

Synthesis of *cis*-tetrahydroactinidiolide (25) via Lipase/Iodine tandem protocol

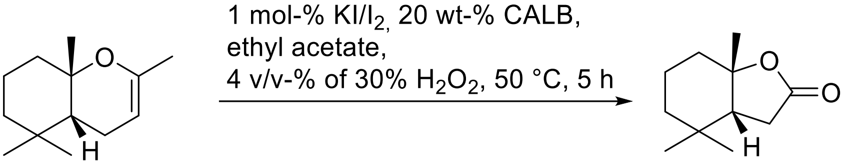

To a 1 mL glass vial was added 500 µL ethyl acetate, 1.0 mg Lipase, 10 µL lugol's iodine (1.0 mol-% iodide) and 5.5 µL cis-hexahydrochromene 23 (4.9 mg, 0.025 mmol, 50 mM). To this was added 9.7 µL of 30% H_2O_2 in H_2O (0.13 mmol, 5 eq.) and the reaction was shaken at 50 °C for 3 h. Afterwards another 9.7 µL of 30% H_2O_2 in H_2O (0.13 mmol) and the reaction was shaken at 50 °C for another 2 h. After reaction completion determined via GC-MS (5 h total reaction time), the reaction was quenched with sat. $Na_2S_2O_3$ and extracted with ethyl acetate. The organic phase was dried over Mg_2SO_4 and reduced under vacuo to afford trans-tetrahydroactinidiolide **1** as a white solid (2.5 mg, 0.014 mmol, 56%).

$C_{11}H_{18}O_2$ **^{1}H-NMR ($CDCl_3$, 700 MHz):** δ (ppm) 0.91 (s, 3H), 1.05 (s, 3H) 1.57 (s, 3H), 1.34-1.63 (m, 5H), 1.84-1.88 (m, 1H), 2.03-2.10 (m, 1H), 2.35-2.57 (m, 2H). **^{13}C-NMR ($CDCl_3$, 176 MHz):** δ (ppm) 19.8 (1C), 20.5 (1C), 20.7 (1C), 29.2 (1C) 32.3 (1C), 32.7 (1C), 37.2 (1C), 40.1 (1C), 55.6 (1C), 86.5 (1C), 178.5 (1C). The data is consistent with the literature.[68]

Synthesis of trans-tetrahydroactinidiolide (1) via Lipase/Iodine tandem protocol

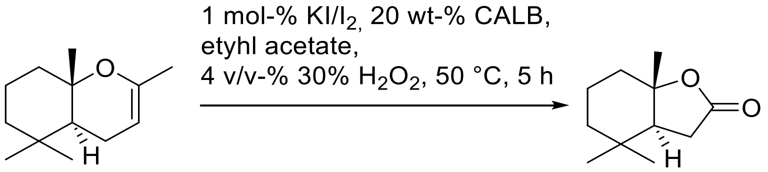

To a 1 mL glass vial was added 500 µL ethyl acetate, 1.0 mg Lipase, 10 µL lugol's iodine (1 mol-% iodide) and 5.5 µL trans-hexahydrochromene 18 (4.9 mg, 0.025 mmol, 50 mM). To

this was added 9.7 µL of 30% H_2O_2 in H_2O (0.13 mmol, 5 eq.) and the reaction was shaken at 50 °C for 3 h. Afterwards another 9.7 µL of 30% H_2O_2 in H_2O (0.13 mmol) and the reaction was shaken at 50 °C for another 2 h. After reaction completion determined via GC-MS (5 h total reaction time), the reaction was quenched with sat. $Na_2S_2O_3$ and extracted with ethyl acetate. The organic phase was dried over Mg_2SO_4 and reduced under vacuo to afford trans-tetrahydroactinidiolide **1** as a white solid (2.7 mg, 0.015 mmol, 59%). For NMR data see 'synthesis of trans-tetrahydroactinidiolide (1) via P-2'.

Synthesis of chromanone (3)

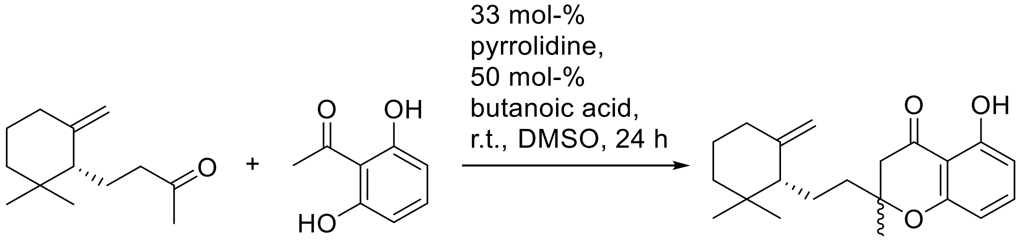

The reaction was carried out under nitrogen atmosphere. To a solution of 24 (50.0 mg, 0.26 mmol) and 2,6-hydroxyacetophenon (39.0 mg, 0.25 mmol) in dry THF (2 mL) and molecular sieves 4Å (400.0 mg), pyrrolodine (6.5 µL, 0.08 mmol) and butanoic acid (11.8 µL, 0.13 mmol) were added. After stirring at room temperature for 24 h, the reaction was extracted with methyl-tert-butyl ether (MTBE, 3x5 mL). The organic phase was washed with 2N HCl (3x5 mL) and brine (3x5 mL), dried over Na_2SO_4 and concentrated in vacuo. Purification by flash chromatography (cyclohexane: MTBE, 95:5) afforded a 50:50 diastereomeric mixture of chromanone **3** (74.0 mg, 0.23 mmol, 90%).

$C_{21}H_{28}O_3$ **^{1}H-NMR ($CDCl_3$, 500 MHz):** δ (ppm) 0.83 (s, 3H), 0.91 (s, 3H), 1.19-1.29 (m, 2H), 1.41-1.83 (m, 9H), 1.89-2.01 (m, 2H), 2.56-2.85 (m, 2H), 4.40 (m, 1H), 4.70 (m, 1H), 6.40 (d, *J* = 1.5 Hz, 1H), 6.45 (d, *J* = 1.5 Hz, 1H), 7.33 t, *J* = 9.0 Hz, 1H). **^{13}C-NMR ($CDCl_3$, 125 MHz):** δ (ppm) 18.9 (1C), 22.6 (1C), 22.8 (1C), 22.9 (1C), 27.3 (1C), 34.0 (1C), 37.0 (1C), 45.8 (1C), 46.0 (1C), 53.0 (1C), 79.9 (1C), 106.6 (1C), 106.8 (1C), 107.5 (1C), 108.3 (1C), 137.2 (1C), 147.7 (1C), 147.8 (1C), 159.0 (1C), 160.7 (1C), 197.5 (1C).

Synthesis of carbonate (2)

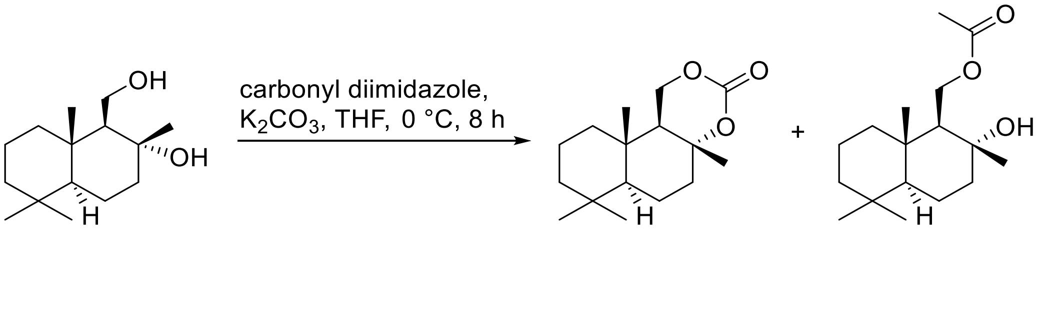

The reaction was carried out under nitrogen atmosphere. To a solution of drimendiol 9 (30.0 mg, 0.13 mmol) in 1 mL dry THF was added potassium carbonate (34.3 mg, 0.25 mmol, 2 eq.) and the reaction mixture was stirred at 0 °C for 10 min. Next, carbonyl diimidazole (40.2 mg, 2 eq.) was added and the reaction was stirred for another 8 h at 0 °C. Upon completion (determined by GC-MS), the reaction was extracted with ethyl acetate, the organic phase was dried over $MgSO_4$ and concentrated under vacuo. Purification by flash chromatography (20:1, cyclohexane:ethyl acetate) afforded **2** mixed 50:50 with acetate S1-2 as a white solid (23.0 mg, 0.08 mmol, 66%). The acetate is presumably generated by unreacted carbonylmonoimidazole-drimenol adduct that reacts with ethyl acetate during quenching, which could potentially be circumvented by longer reaction times.

Dioxanone 2 $C_{16}H_{26}O_3$ **^{1}H-NMR ($CDCl_3$, 500 MHz):** δ (ppm) 0.83 (s, 3H), 0.89 (s, 3H), 0.91 (s, 3H), 1.31-1.51 (m, 6H), 1.48 (s, 3H), 1.63-1.74 (m, 6H), 2.05 (m, 2H), 4.33-4.42 (m, 2H). **^{13}C-NMR ($CDCl_3$, 125 MHz):** δ (ppm) 15.4 (1C), 18.1 (1C), 19.5 (1C), 21.4 (1C), 21.7 (1C), 33.01 (1C), 33.3 (1C), 36.3 (1C), 39.0 (1C) 39.9 (1C), 41.5 (1C), 51.3 (1C), 55.8 (1C), 66.9 (1C), 82.2 (1C), 149.1 (1C).

Drimenol acetate S1-2 $C_{17}H_{30}O_3$ **^{1}H-NMR ($CDCl_3$, 500 MHz):** δ (ppm) 0.81 (s, 3H), 0.86 (s, 3H), 0.88 (s, 3H), 0.93-0.97 (m, 1H), 1.18 (s, 3H), 1.29-1.63 (m, 9H), 1.62-1.69 (m, 2H), 1.87-1.91 (dt, J_d = 6.4 Hz, J_t = 3.1 Hz, 1H), 2.05 (s, 3H), 4.23-4.37 (qd, J_q = 27.3 Hz, J_d = 2.4 Hz, 2H). **^{13}C-NMR ($CDCl_3$, 125 MHz):** δ (ppm) 15.8 (1C), 18.4 (1C), 20.3 (1C), 21.3 (1C), 21.5 (1C), 24.6 (1C), 33.2 (1C), 33.5 (1C), 38.1 (1C), 39.7 (1C), 41.7 (1C), 43.9 (1C), 55.7 (1C), 59.9 (1C) 62.6 (1C), 72.6 (1C), 171.3.

Synthesis of α-pyrone meroterpenes (28) and (29)

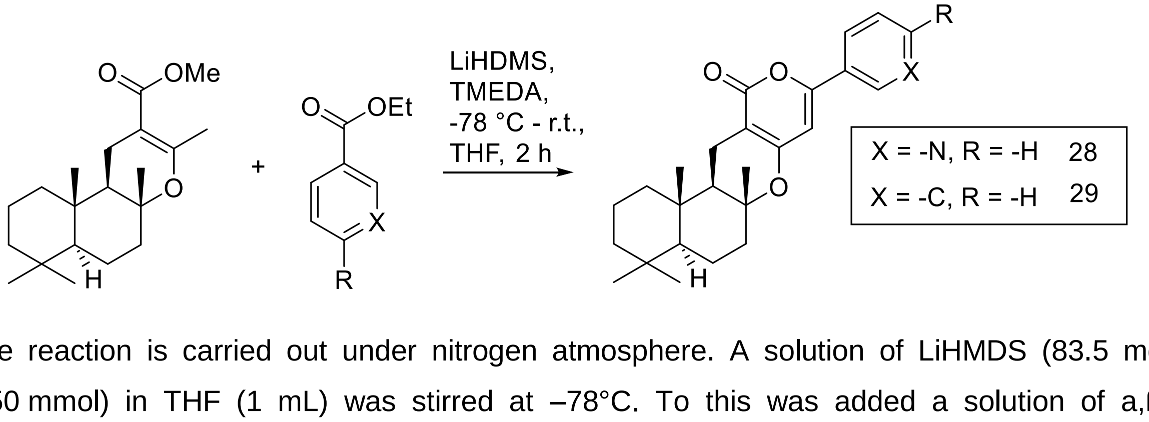

The reaction is carried out under nitrogen atmosphere. A solution of LiHMDS (83.5 mg, 0.50 mmol) in THF (1 mL) was stirred at –78°C. To this was added a solution of a,ß-unsaturated ester 16 (40.0 mg, 0.13 mmol) in 3 mL of dry THF. The mixture was stirred for 25 min and then treated with nicotinoate (57.0 mg, 0.38 mmol) or benzoate (53.6 µL, 0.38 mmol). During the reaction, the mixture was allowed to warm up to room temperature to facilitate the intramolecular Aldol condensation. After 2 h the reaction mixture was quenched with H_2O

(15 mL) and extracted with EtOAc (3x20 mL). The combined organic layers were washed with brine (10 mL), dried over $MgSO_4$ and purified by flash chromatography (CH:EtOAc, 20:1) to afford bright yellow 28 (31.0 mg, 0.080 mmol, 64%). For product 29, 108 µL (0.13 mmol) mesitylene as an internal standard was added to determine the yield. (0.036 mmol, 28%)

Dehydroxypyropene 28 $C_{25}H_{31}NO_3$ **^{1}H-NMR ($CDCl_3$, 500 MHz):** δ (ppm) 0.85 (s, 3H), 0.90 (s, 3H), 0.91 (s, 3H), 1.26 (s, 3H), 1.31-1.54 (m, 5H), 1.63-1.83 (m, 6H), 2.10-2.14 (dt, J_d = 8.5 Hz, J_d = 2.4 Hz, 1H), 2.18-2.27 (m, 1H), 2.50-2.57 (dd, J_d = 6.2 Hz, J_t = 3.4 Hz, 1H), 6.43 (s, 1H), 7.36-7.31 (m, 1H), 8.10 (dt, J_d = 4.4 Hz, J_t = 1.8 Hz, 1H), 8.65 (dd, J_d = 2.3 Hz, J_d = 0.7 Hz, 1H), 9.00 (d, J = 1 Hz, 1H). **^{13}C-NMR ($CDCl_3$, 125 MHz):** δ (ppm) 15.1 (1C), 17.2 (1C), 18.4 (1C), 19.7 (1C), 20.8 (1C), 21.5 (1C) 33.2 (1C), 33.4 (1C), 37.1 (1C), 39.2 (1C), 40.4 (1C), 41.7 (1C), 51.7 (1C), 56.0 (1C), 81.3 (1C), 99.5 (1C), 100.5 (1C), 123.6 (1C), 127.7 (1C), 132.8 (1C), 146.6 (1C), 151.0 (1C), 155.5 (1C), 162.9 (1C), 164.2 (1C).

Chlorosulfonic acid catalyzed cyclizations of terpenes as racemic standards

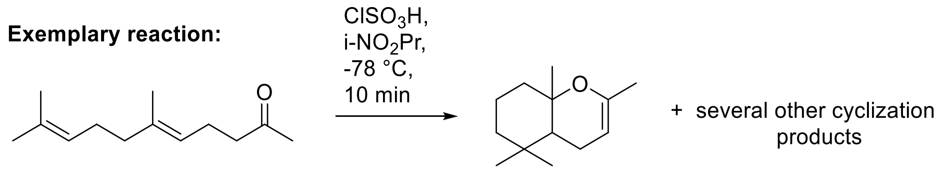

The reactions were carried out under nitrogen atmosphere. To a solution of 5 Eq. (2.5 mmol, 160µL) of chlorosulfonic acid in 2-nitropropane (3 mL) was added a solution of terpene (0.5 mmol, 1 eq.) in 2-nitropropane (6 mL) at –78 °C and was stirred for 10 min. The reaction was quenched by adding saturated aq. $NaHCO_3$ solution (10 ml) and then further portions of solid $NaHCO_3$ were added to obtain basic pH. The reaction mixture was extracted with Et_2O (3x 15 mL), dried over $MgSO_4$ and the resulting crude product was used for chiral GC analysis. For chromatograms see G.

F. Chromatograms

The following GC chromatograms show the chlorosulfonic acid-catalyzed cyclization of the substrate in the scheme in (a) and the enzyme-catalyzed reaction in (b) and (c). Chiral chromatograms are shown in the enzyme-catalyzed reaction. For (–)-γ-dihydroionone **24** stereochemistry please see ref. [8].

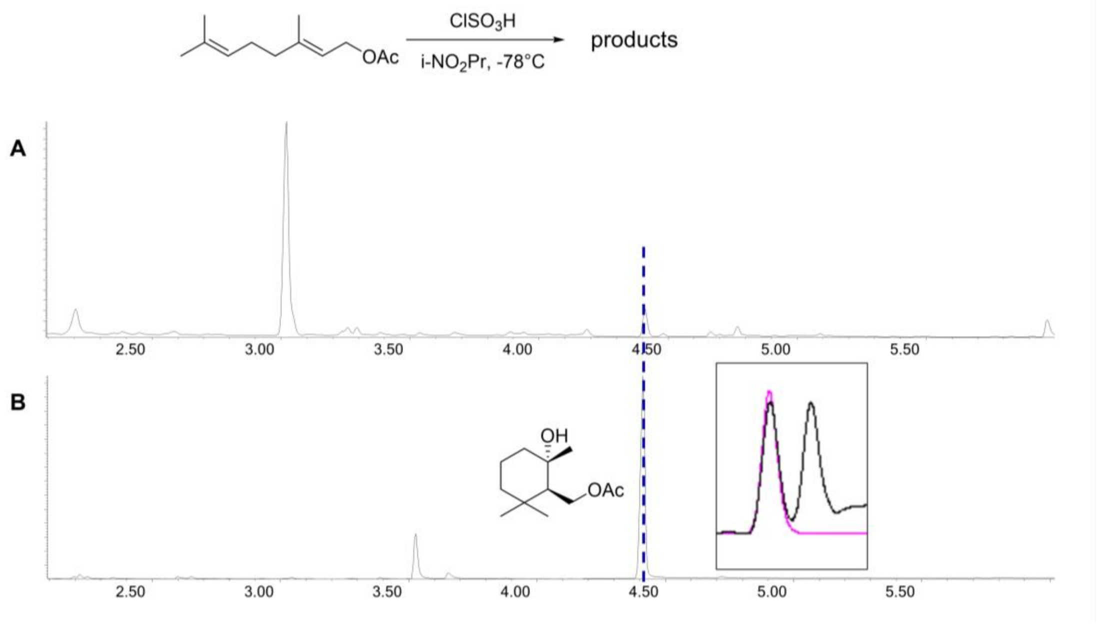

Supplementary Figure 23: Ger*an*yl acetate 6 cyclization (a) using chlorosulfonic acid and (b) AacSHC G600R including the chiral chromatogram.

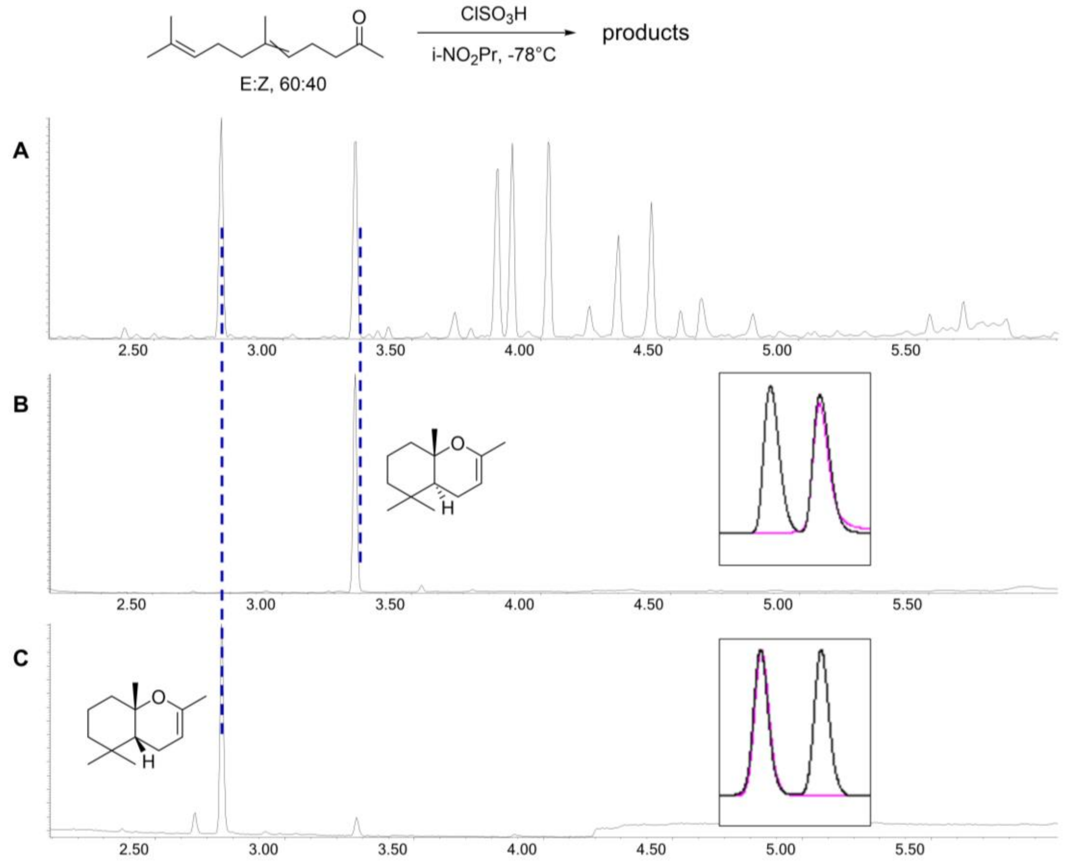

Supplementary Figure 24: Geranyl acetone 17 and neryl acetone cyclization Z-17 using (a) chlorosulfonic acid and (b) and (c) AacSHC G600M including the chiral chromatograms.

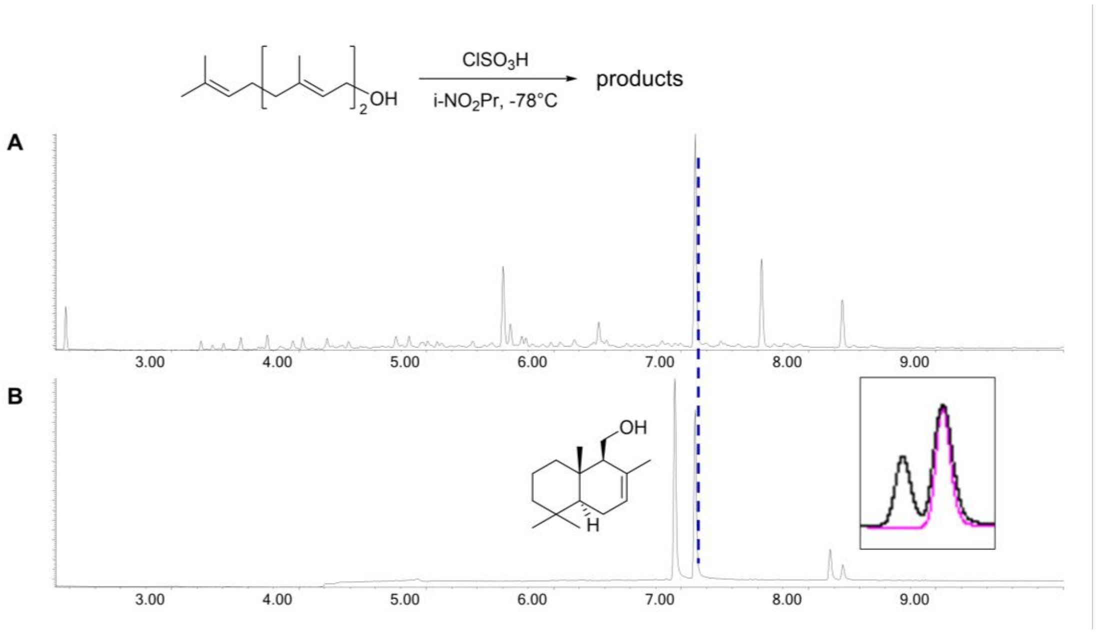

Supplementary Figure 25*:* Farnesol 8 cyclization using (a) chlorosulfonic acid and (b) AacSHC A306W including the chiral chromatogram. The dehydration of 9 on the GC column led to a formation of deprotonation products γ- and α-Drimenol in a ratio of 55:45.

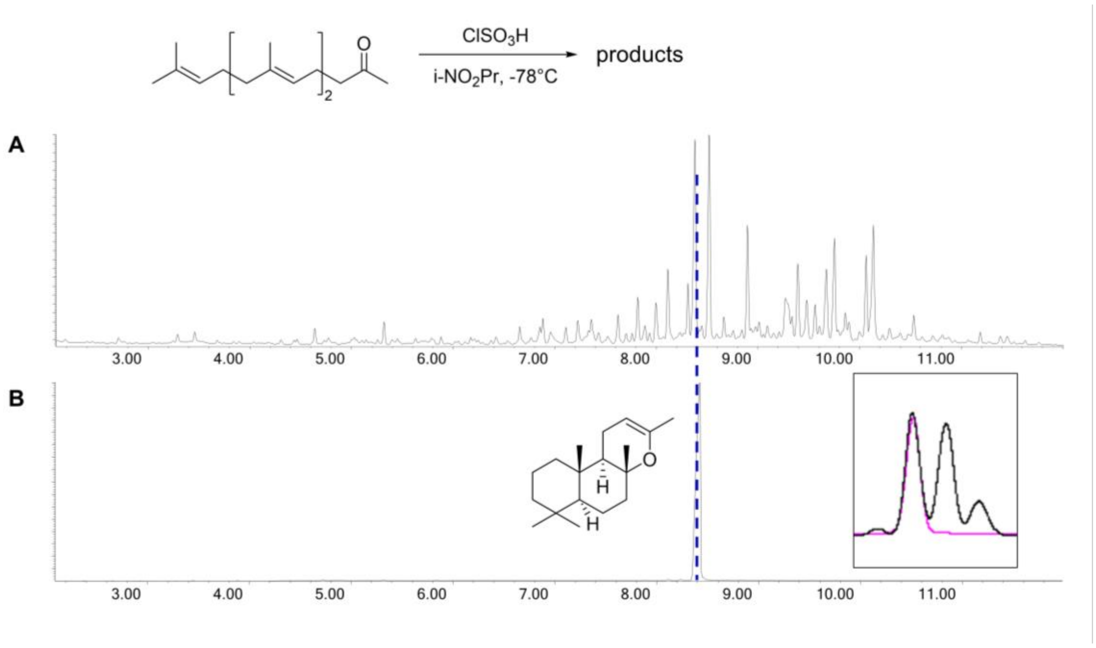

Supplementary Figure 26: Farnesyl acetone 13 cyclization using (a) chlorosulfonic acid and (b) AacSHC F601D F605L including the chiral chromatogram.

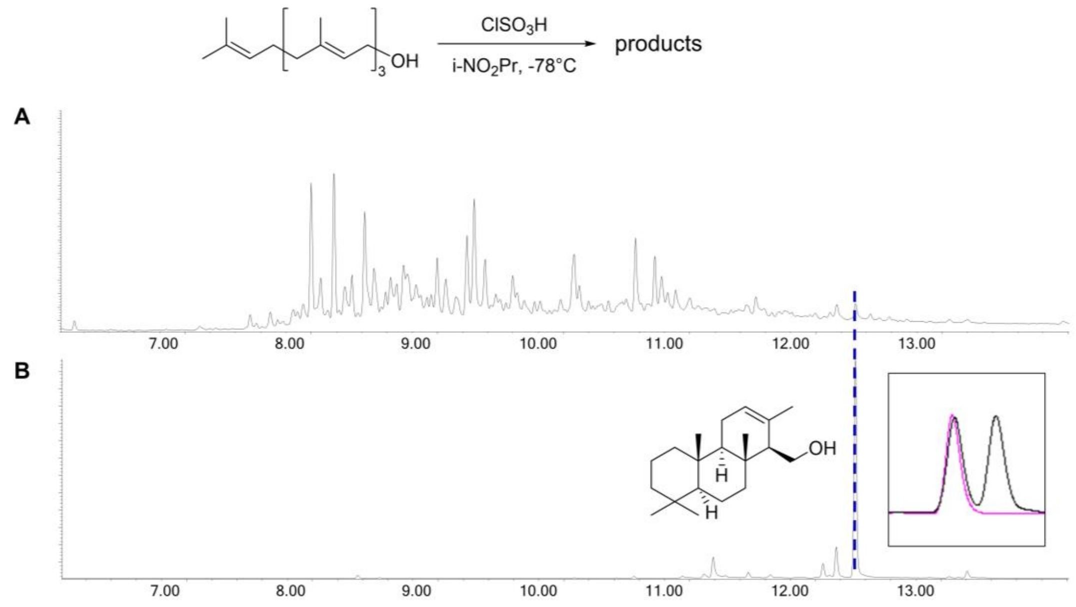

Supplementary Figure 27: Geranyl geraniol 10 cyclization using (a) chlorosulfonic acid and (b) AacSHC F605W including the chiral chromatogram.

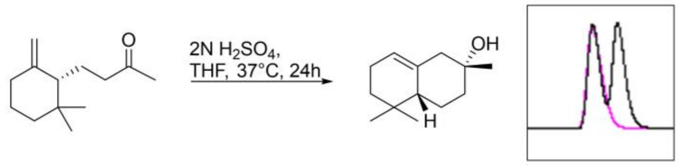

Supplementary Figure 28: (–)-γ-dihydroionone 24 cyclization to (+)-α-ambrinol using sulfuric acid.[8]

G. Mass spectra

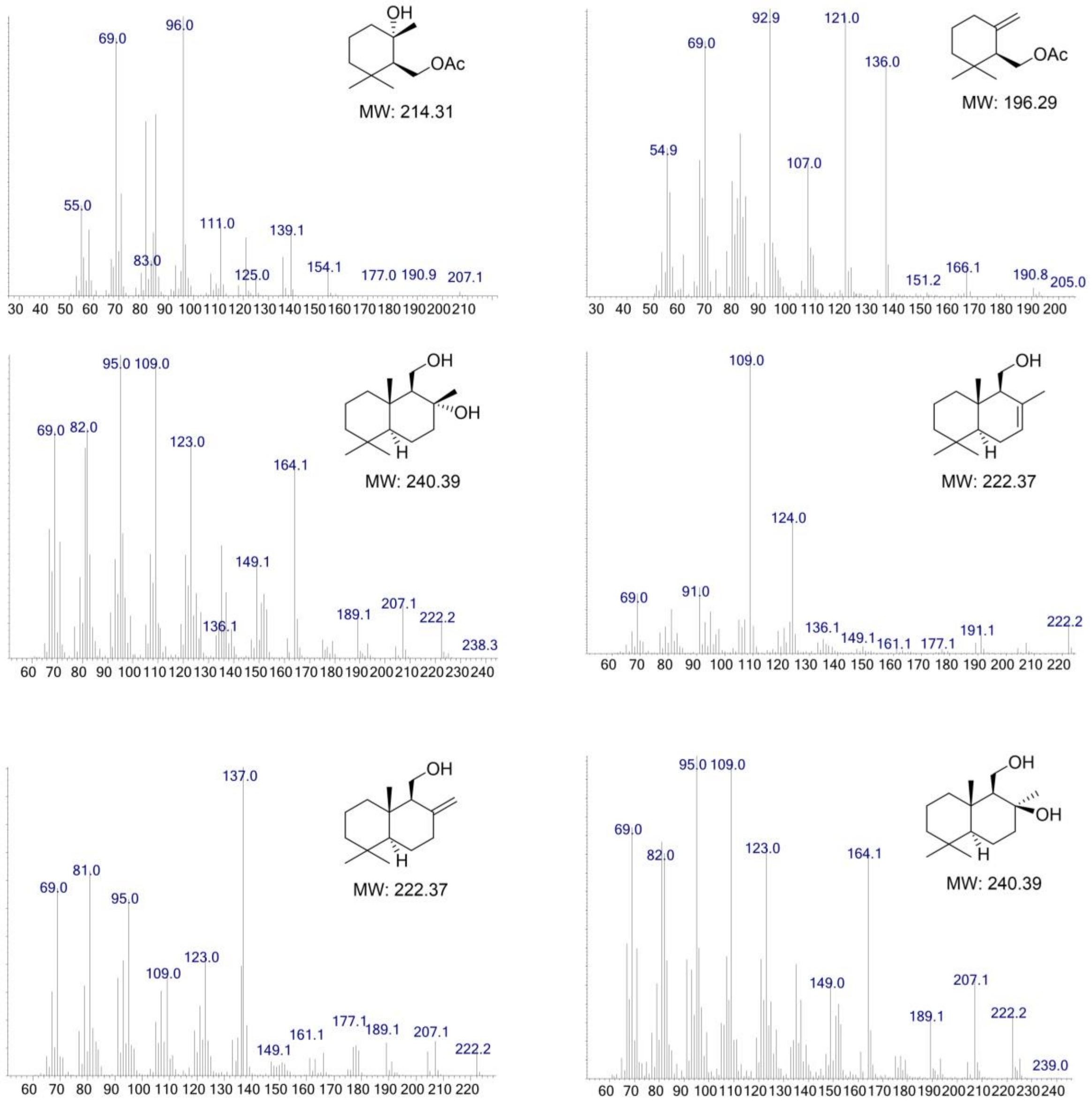

Supplementary Figure 29: Mass spectra of compounds 7, S1-7, 9, S2-9, S3-9, S1-9.

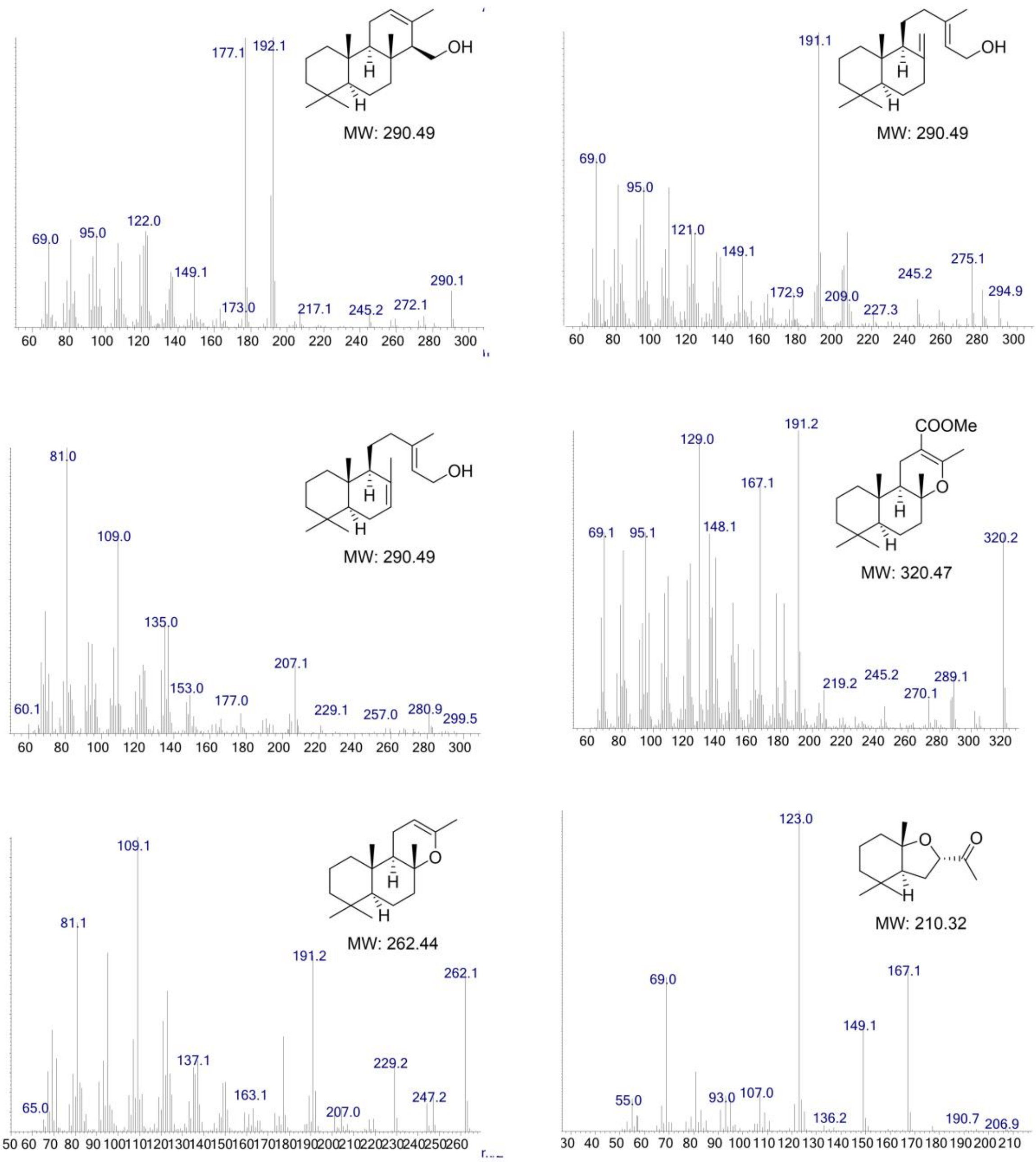

Supplementary Figure 30: Mass spectra of compounds 11, S1-11, 12, 16, 14 and P-1.

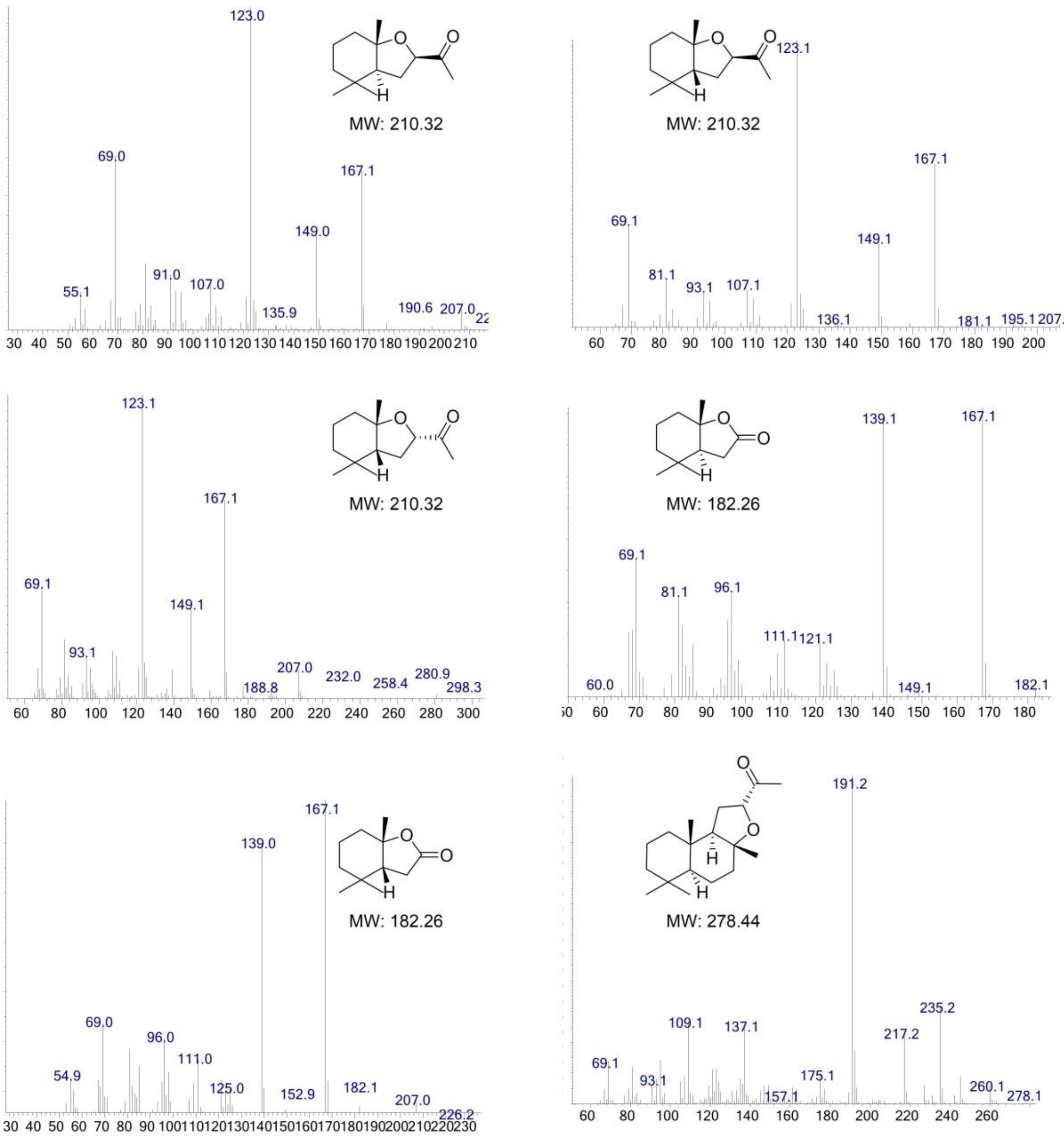

Supplementary Figure 31: Mass spectra of compounds P-4, P-2, P-5, 1, 25 and P-3.

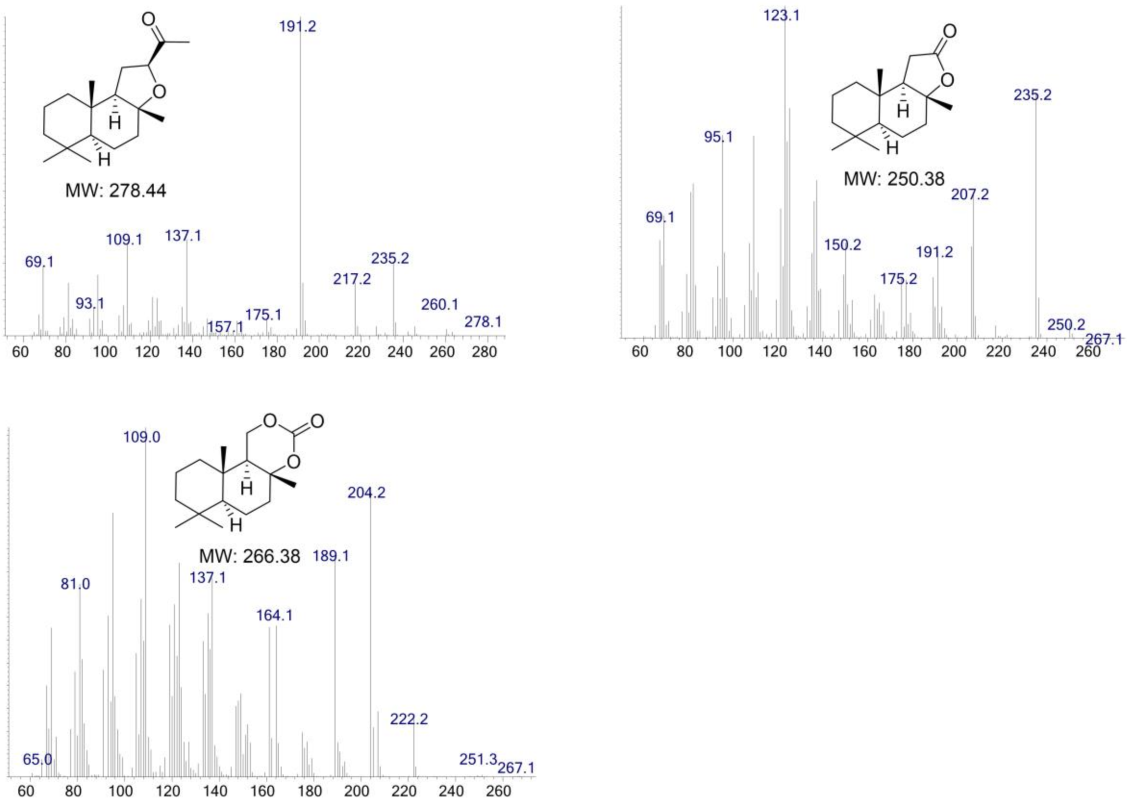

Supplementary Figure 32: Mass spectra of compounds P-6, 26 and 2.

H. NMR spectra

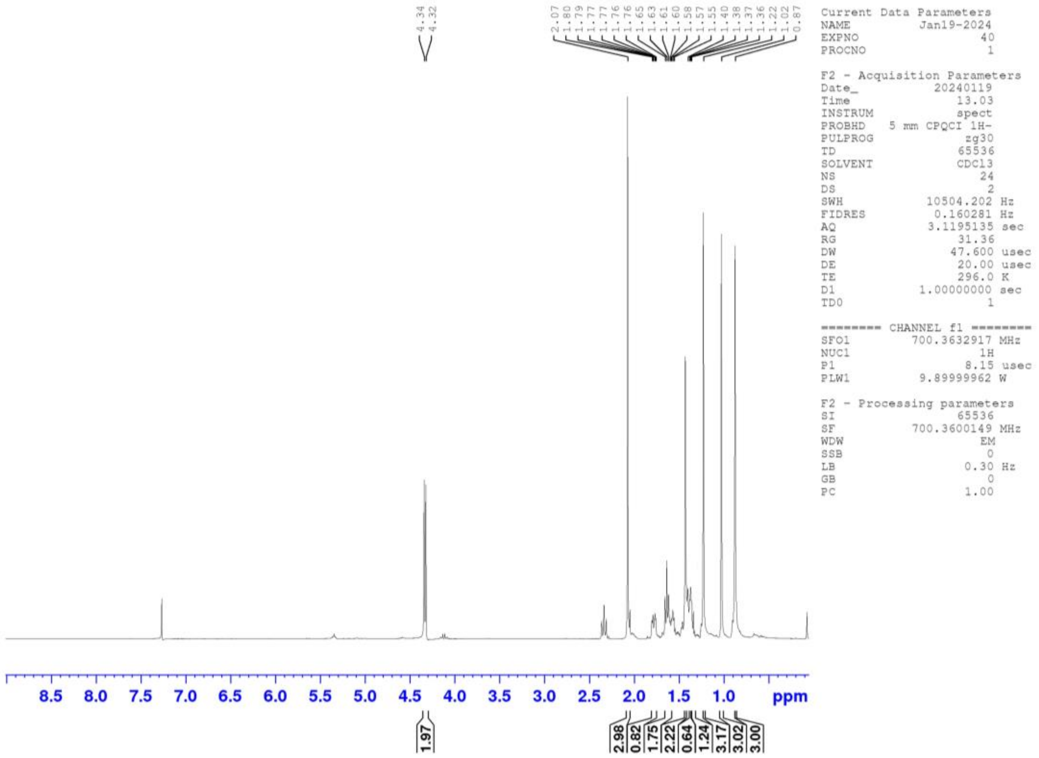

Supplementary Figure 33: ^{1}H of cyclogeranyl acetate hydrate (8)

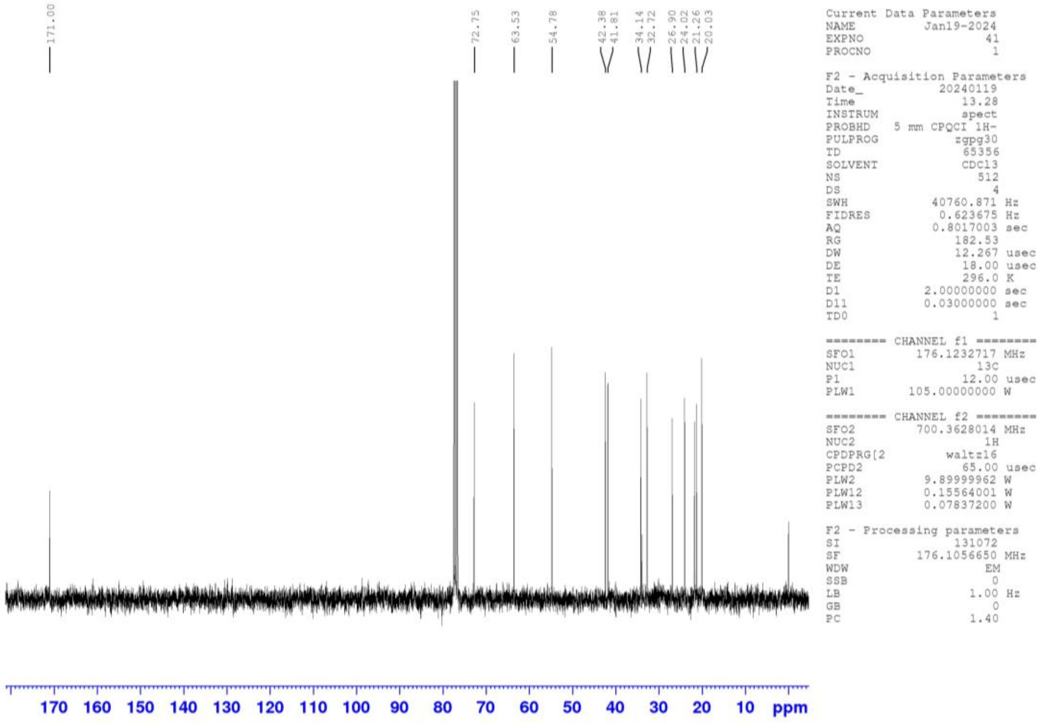

Supplementary Figure 34: ^{13}C of cyclogeranyl acetate hydrate (8)

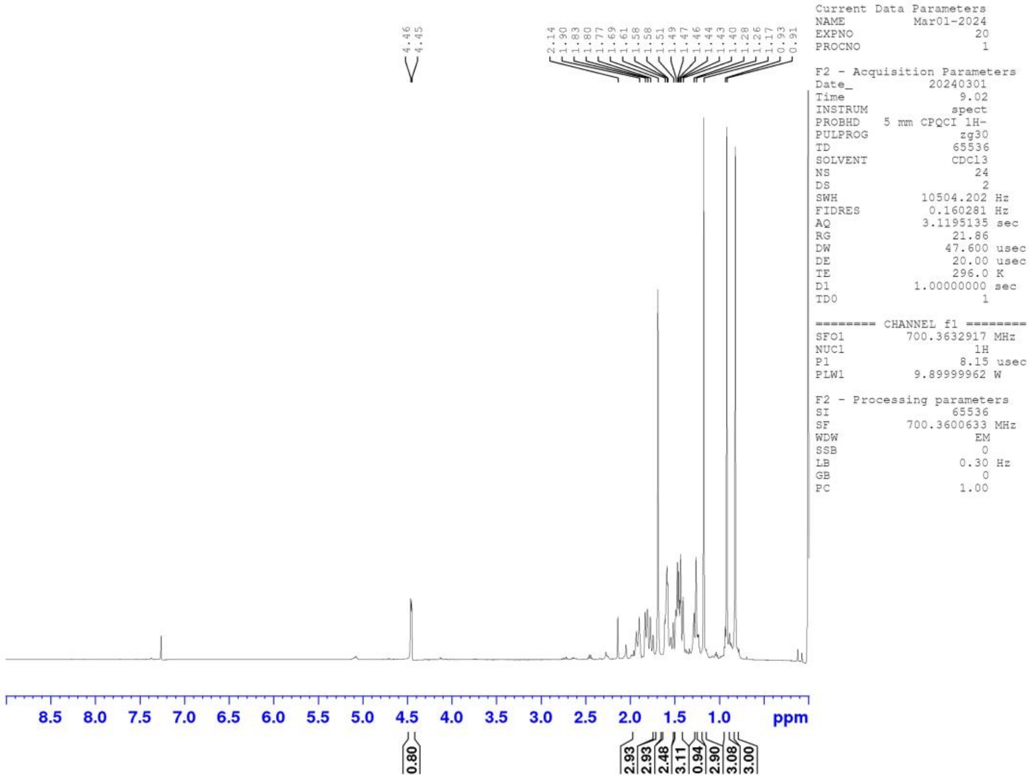

Supplementary Figure 35: ^{1}H of trans-hexahydrochromene (18)

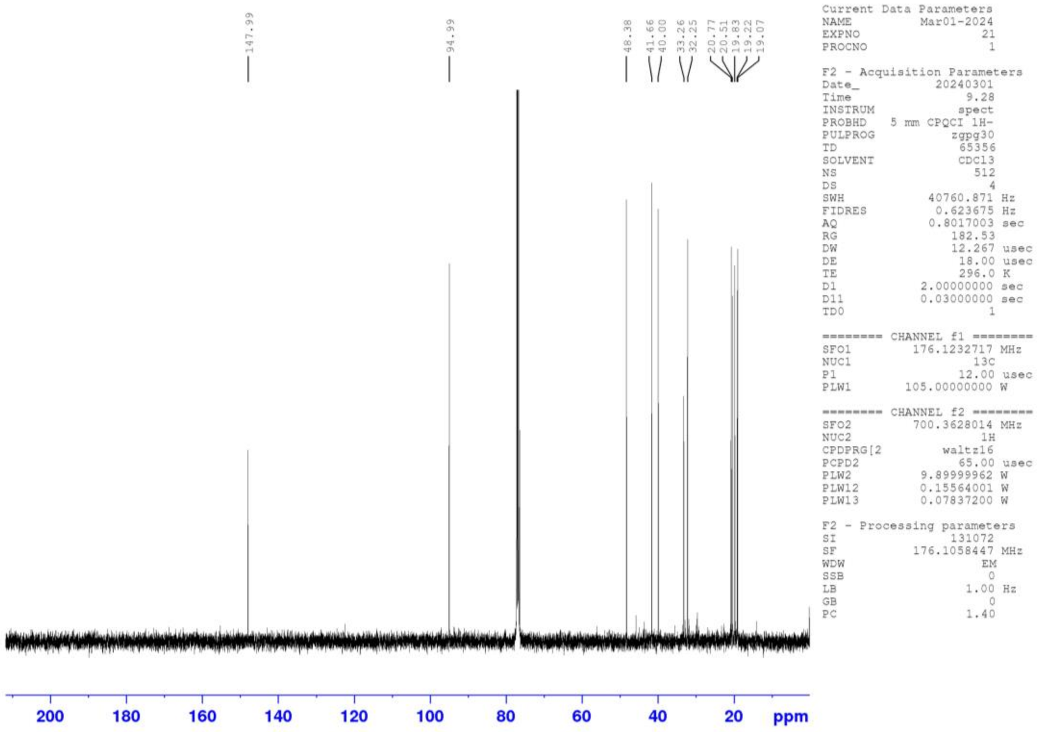

Supplementary Figure 36: ^{13}C of trans-hexahydrochromene (18)

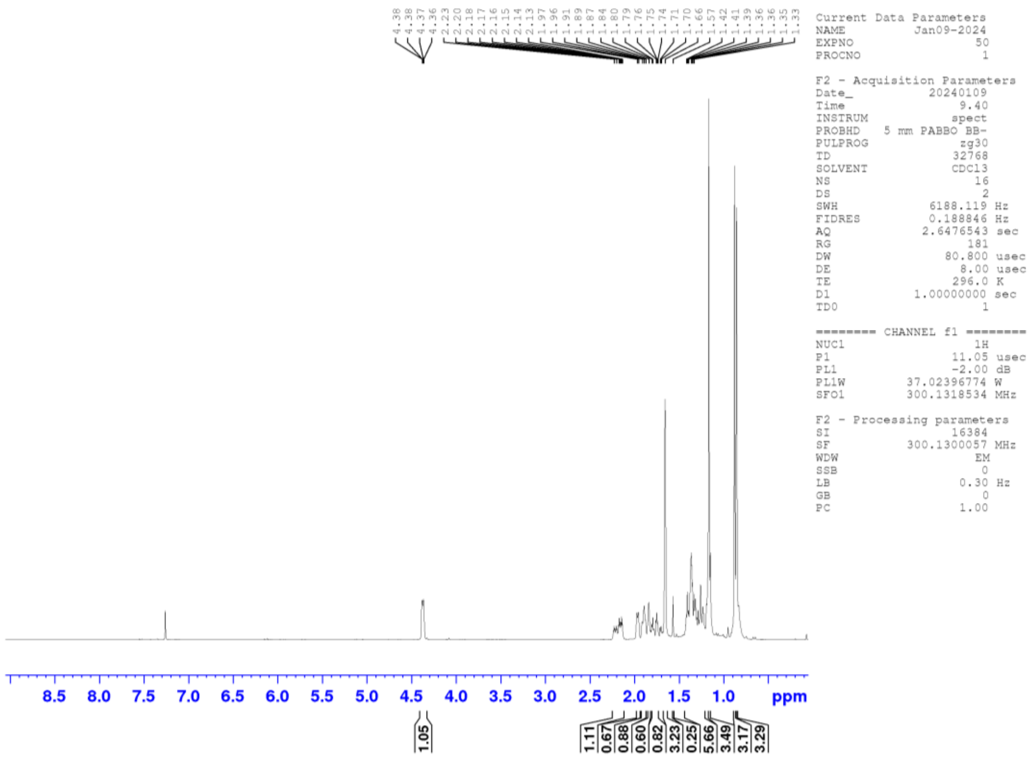

Supplementary Figure 37: ^{1}H of cis-hexahydrochromene (23)

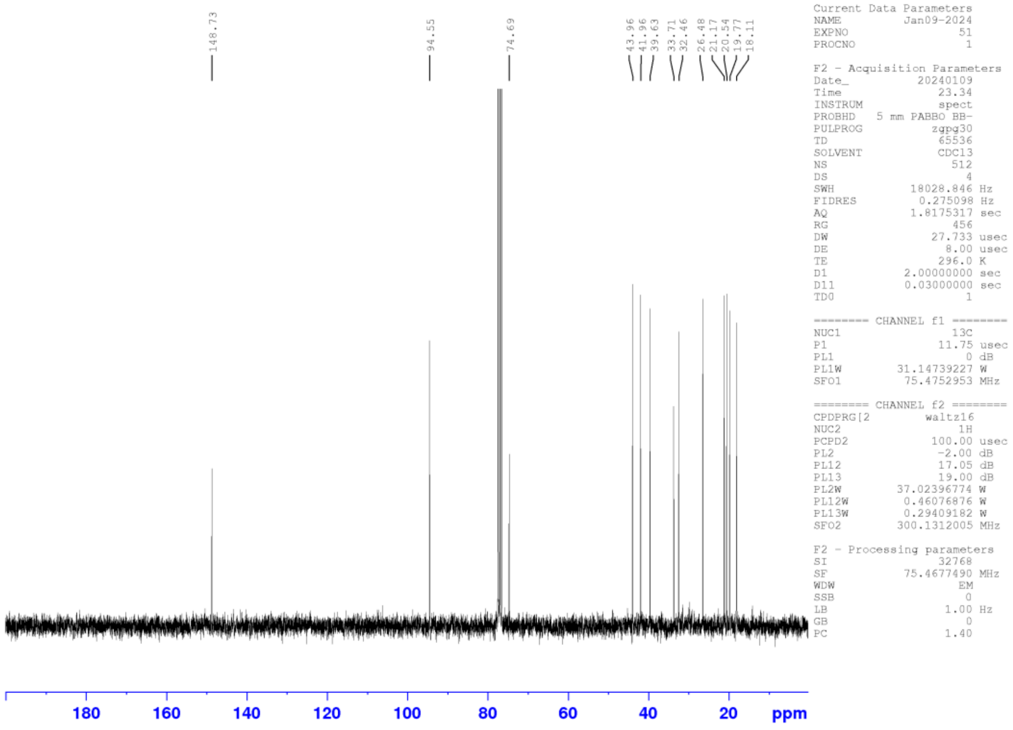

Supplementary Figure 38: ^{13}C of cis-hexahydrochromene (23)

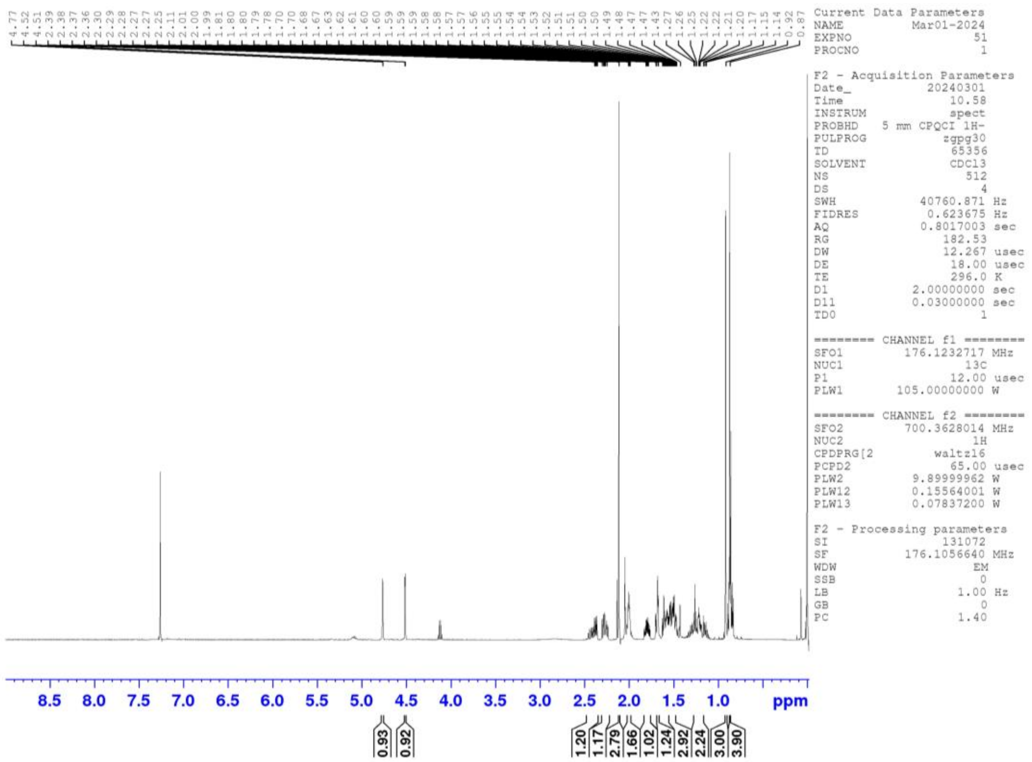

Supplementary Figure 39: ^{1}H of γ-(–)-dihydroionone (24)

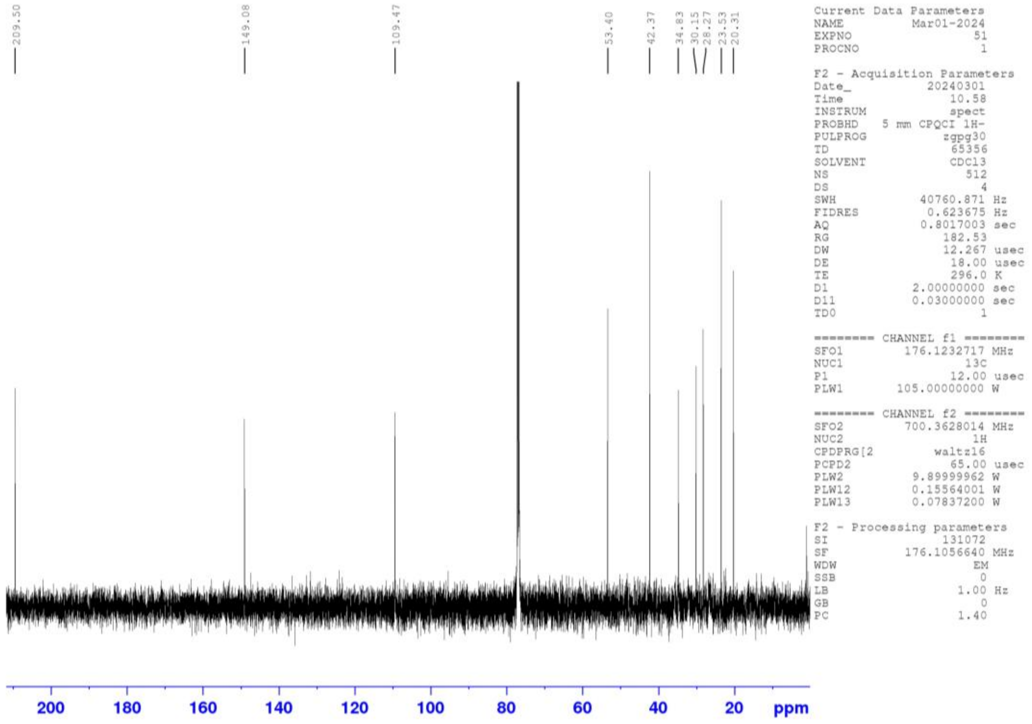

Supplementary Figure 40: ^{13}C of γ-(–)-dihydroionone (24)

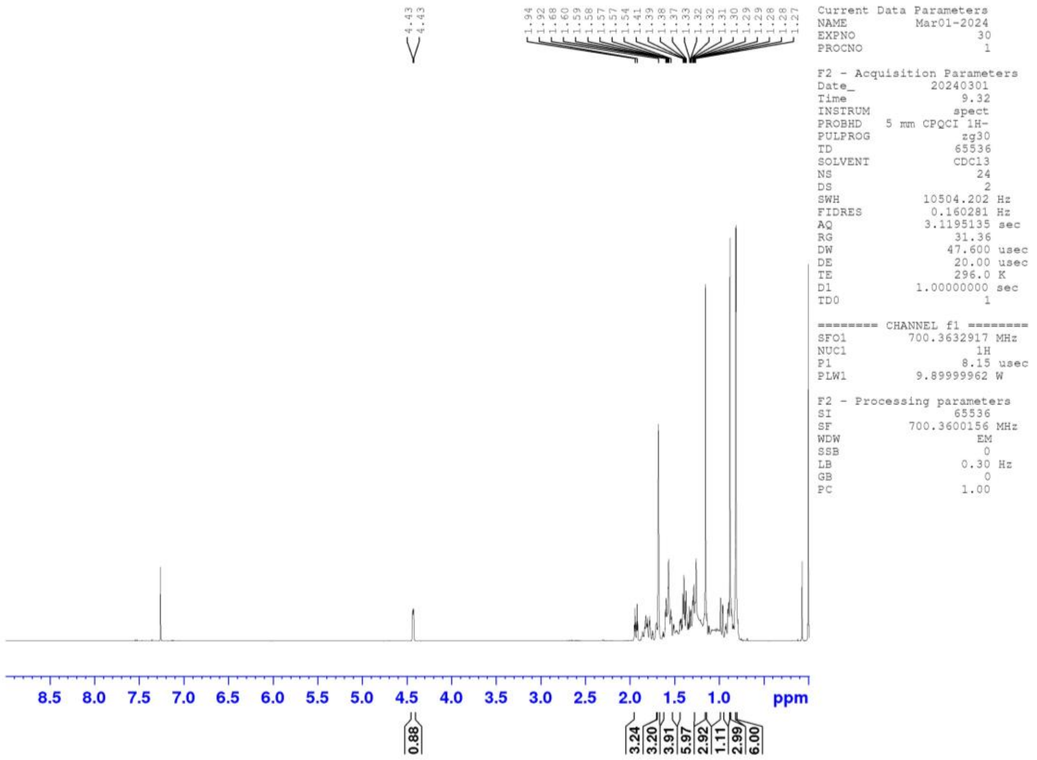

Supplementary Figure 41: ^{1}H of sclareoloxide (14)

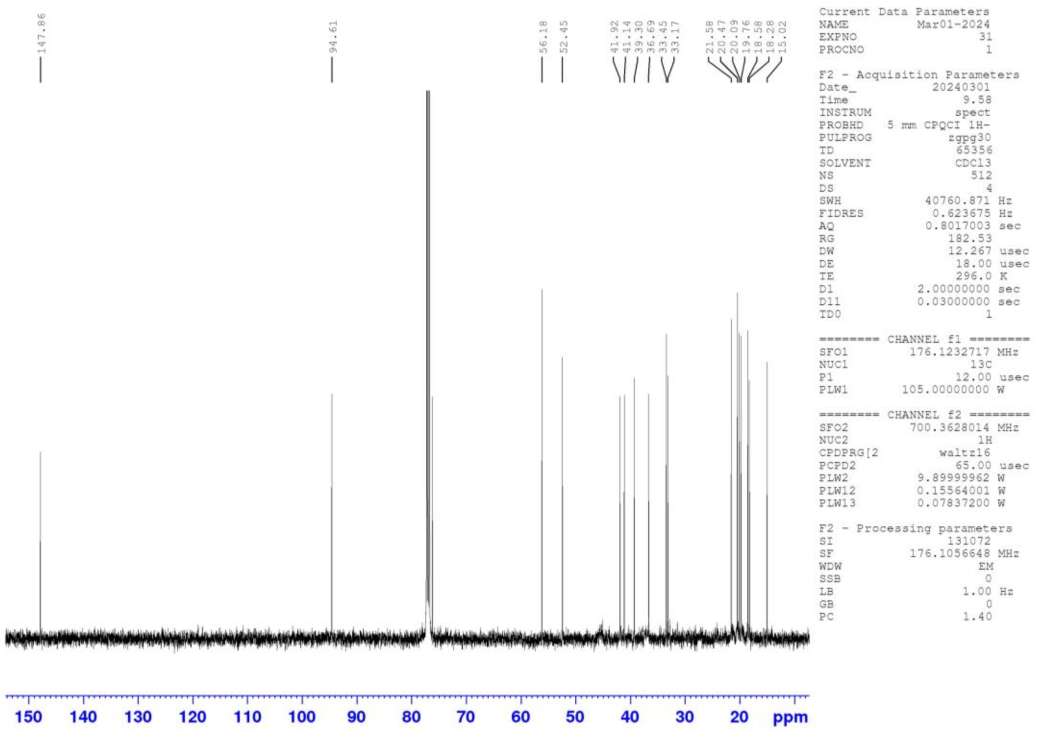

Supplementary Figure 42: ^{13}C of sclareoloxide (14)

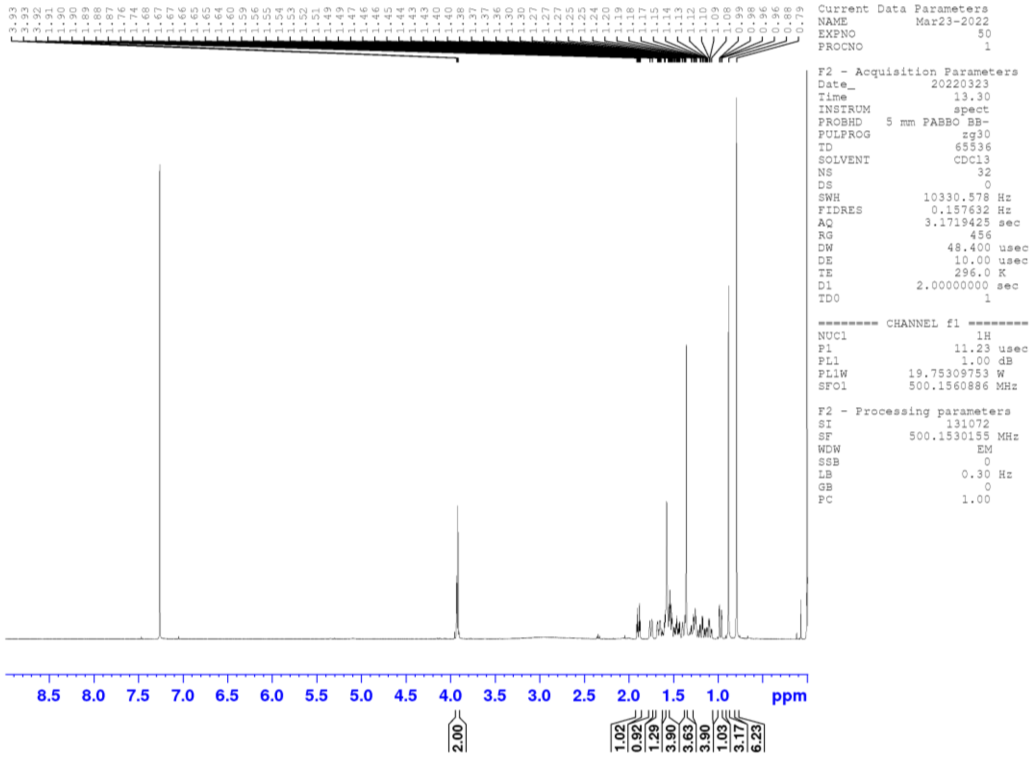

Supplementary Figure 43: ^{1}H of *S*,*R*,*S*,*S*-drimendiol (9)

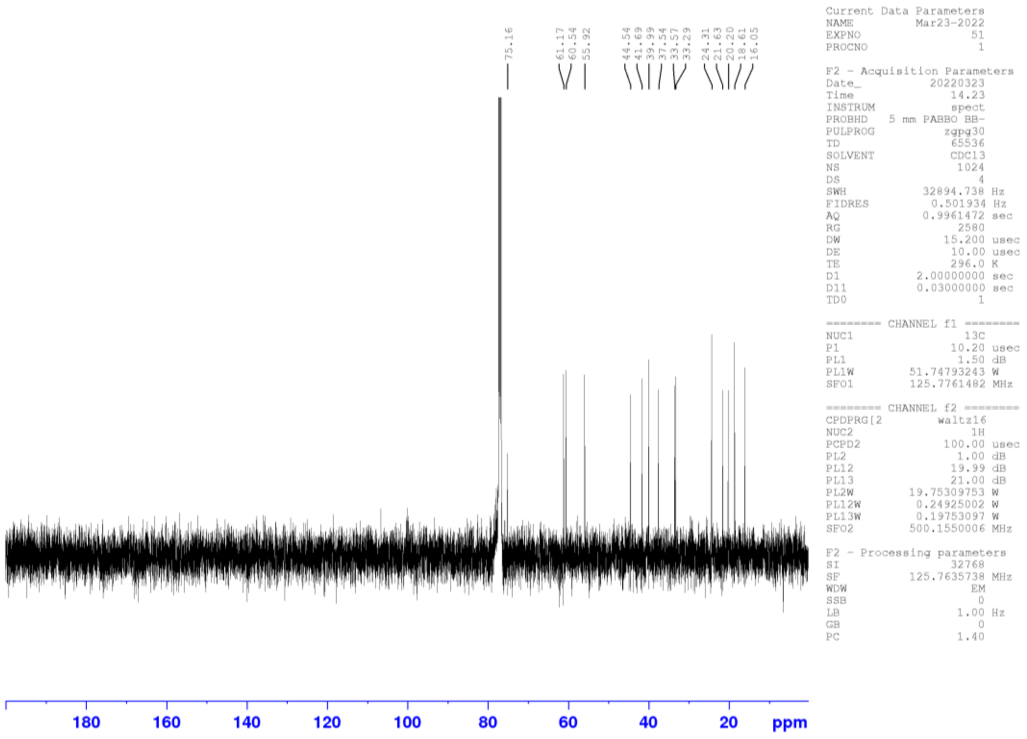

Supplementary Figure 44: ^{13}C of *S*,*R*,*S*,*S*-drimendiol (9)

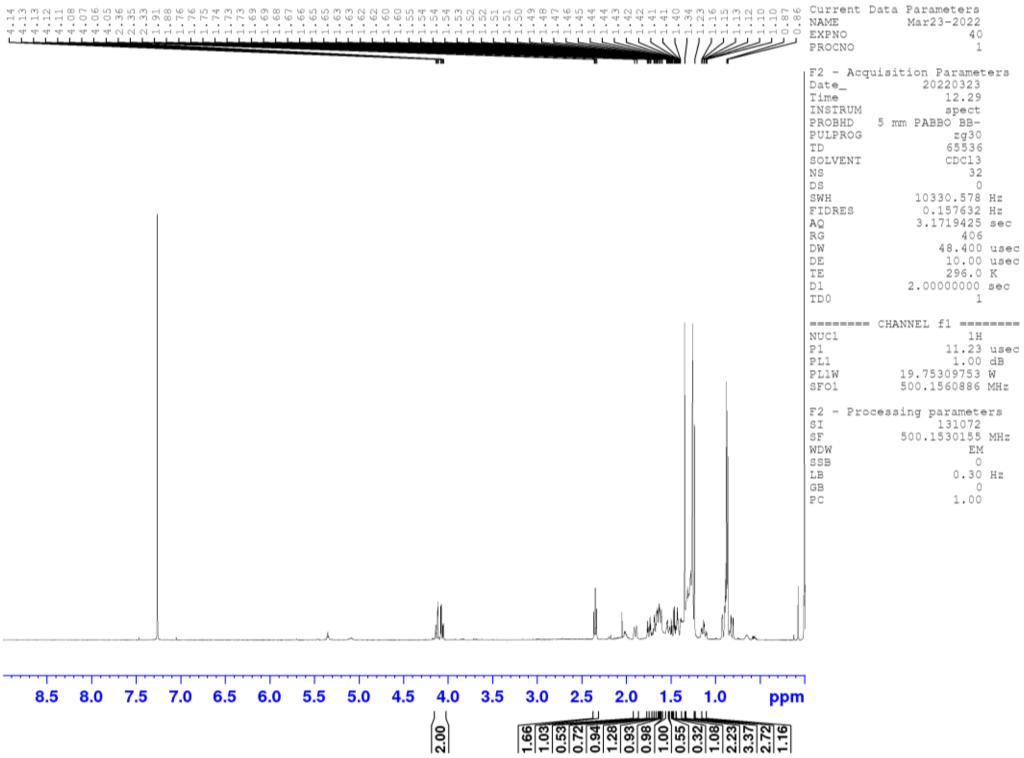

Supplementary Figure 45: ^{1}H of *S*,*S*,*S*,*S*-drimendiol (S1-9)

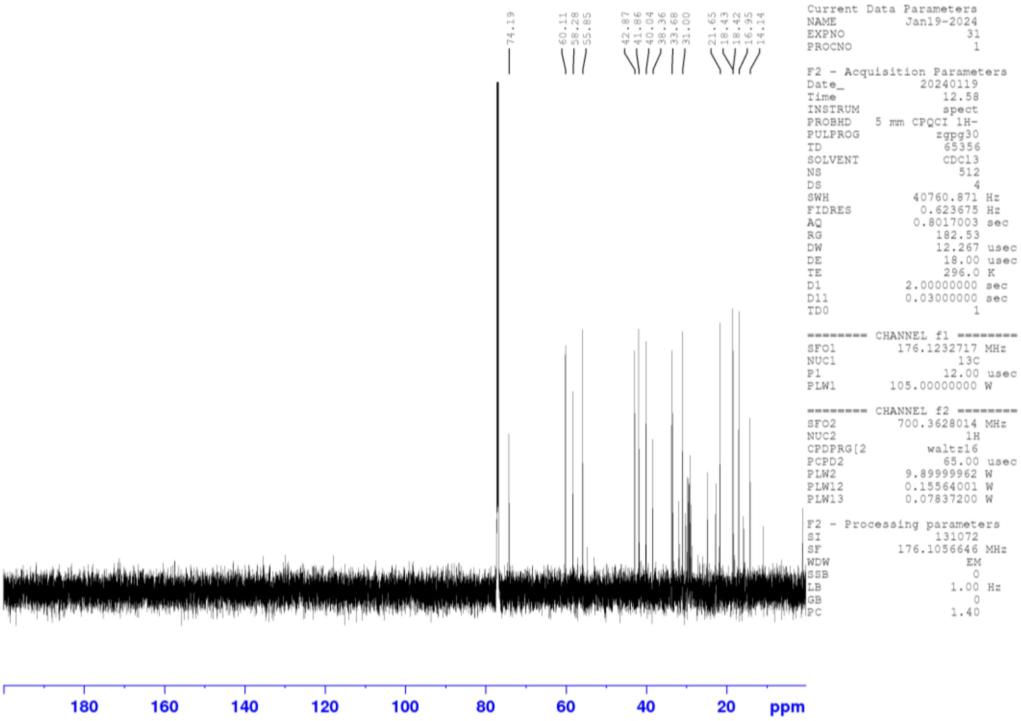

Supplementary Figure 46: ^{13}C of *S*,*S*,*S*,*S*-drimendiol (S1-9)

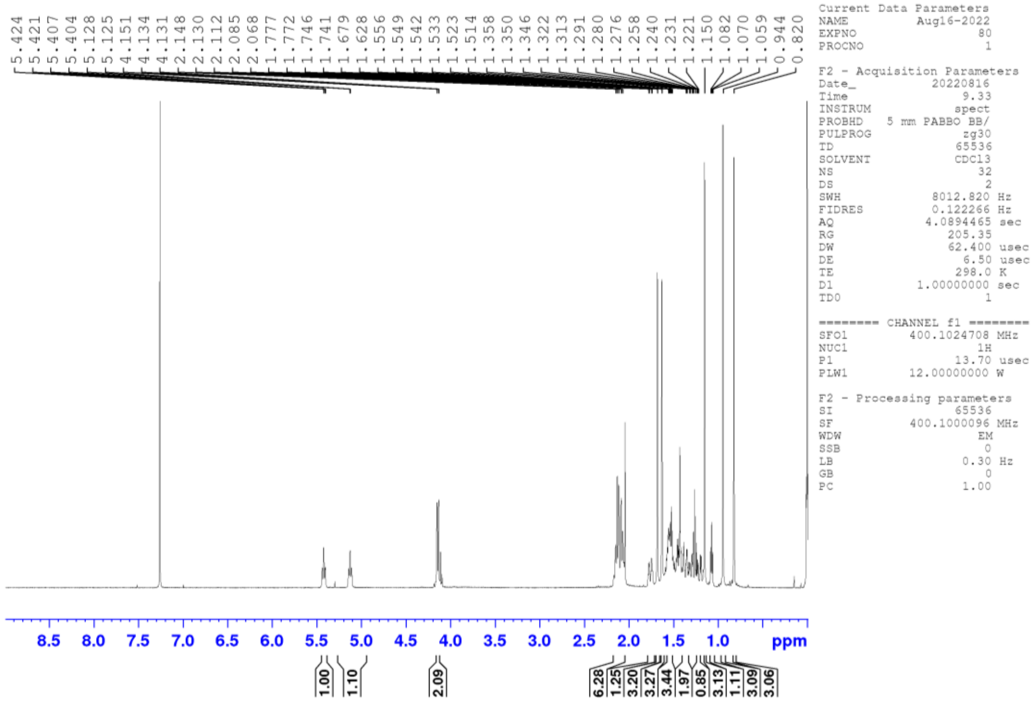

Supplementary Figure 47: ^{1}H of labdane (12)

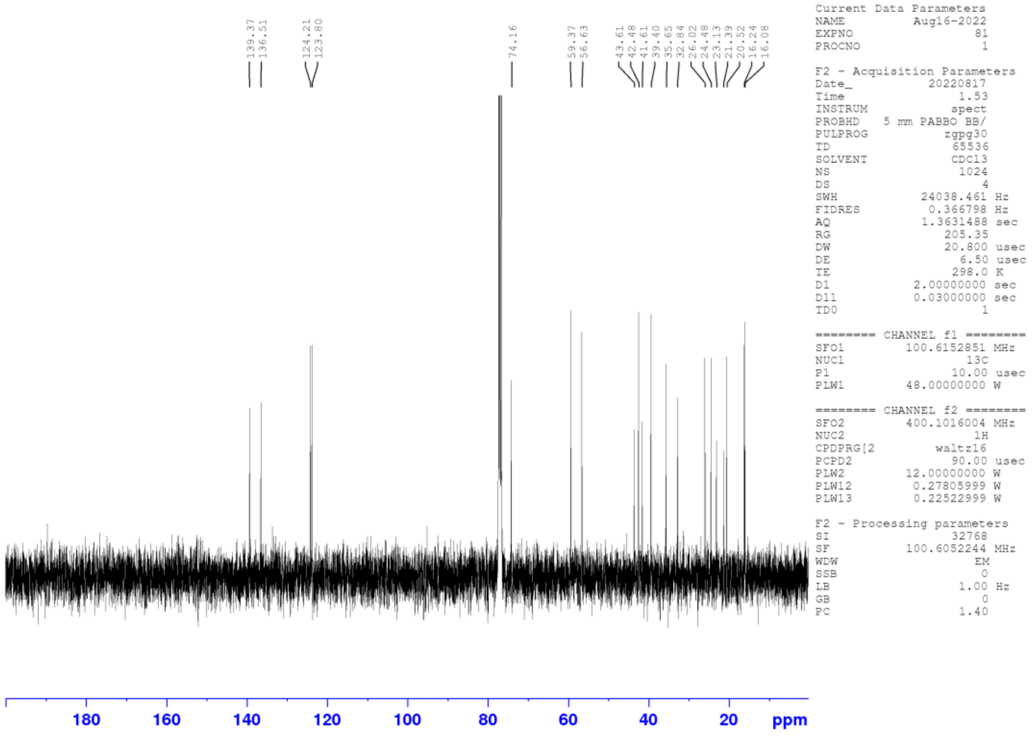

Supplementary Figure 48: ^{13}C of labdane (12)

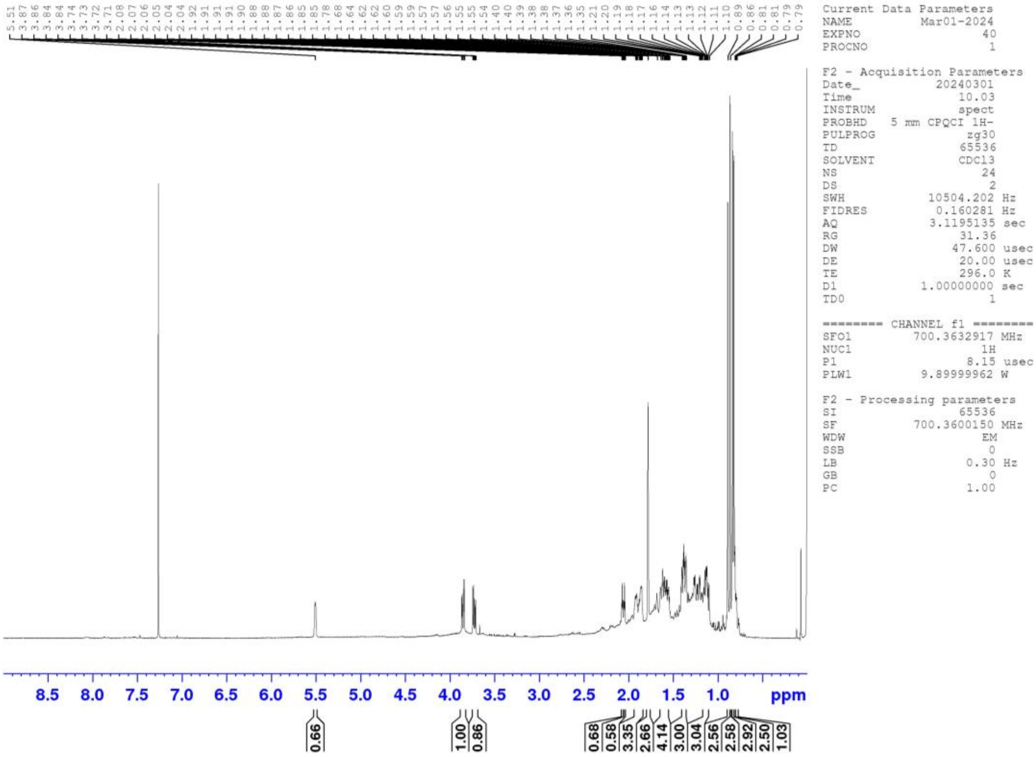

Supplementary Figure 49: ^{1}H of *ent*-isocopolol (11)

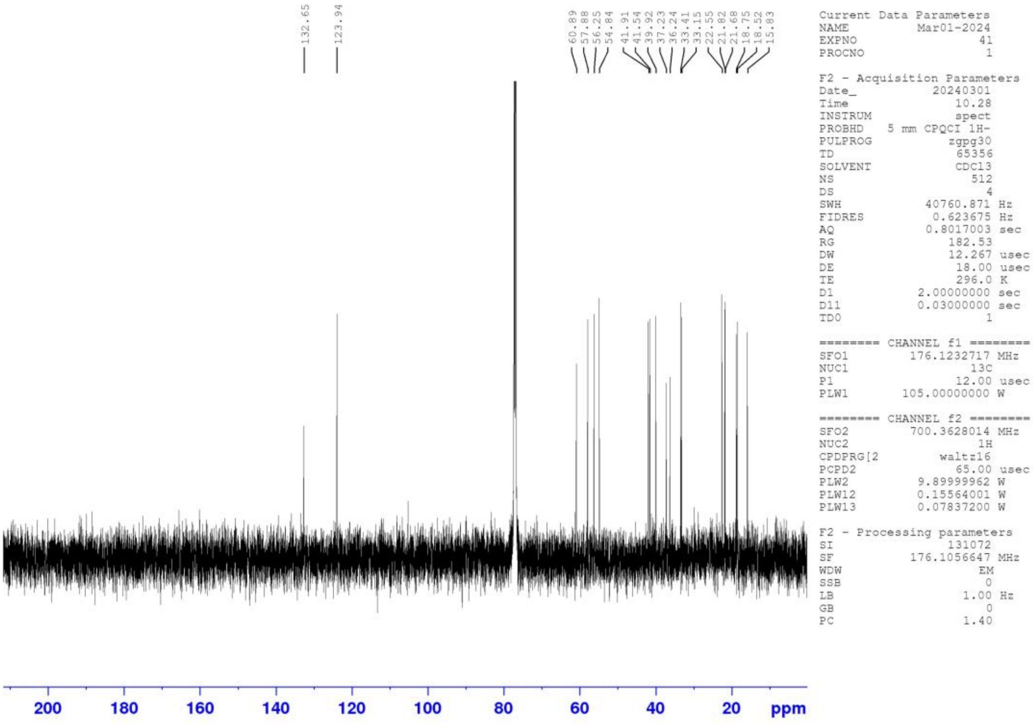

Supplementary Figure 50: ^{13}C of *ent*-isocopolol (11)

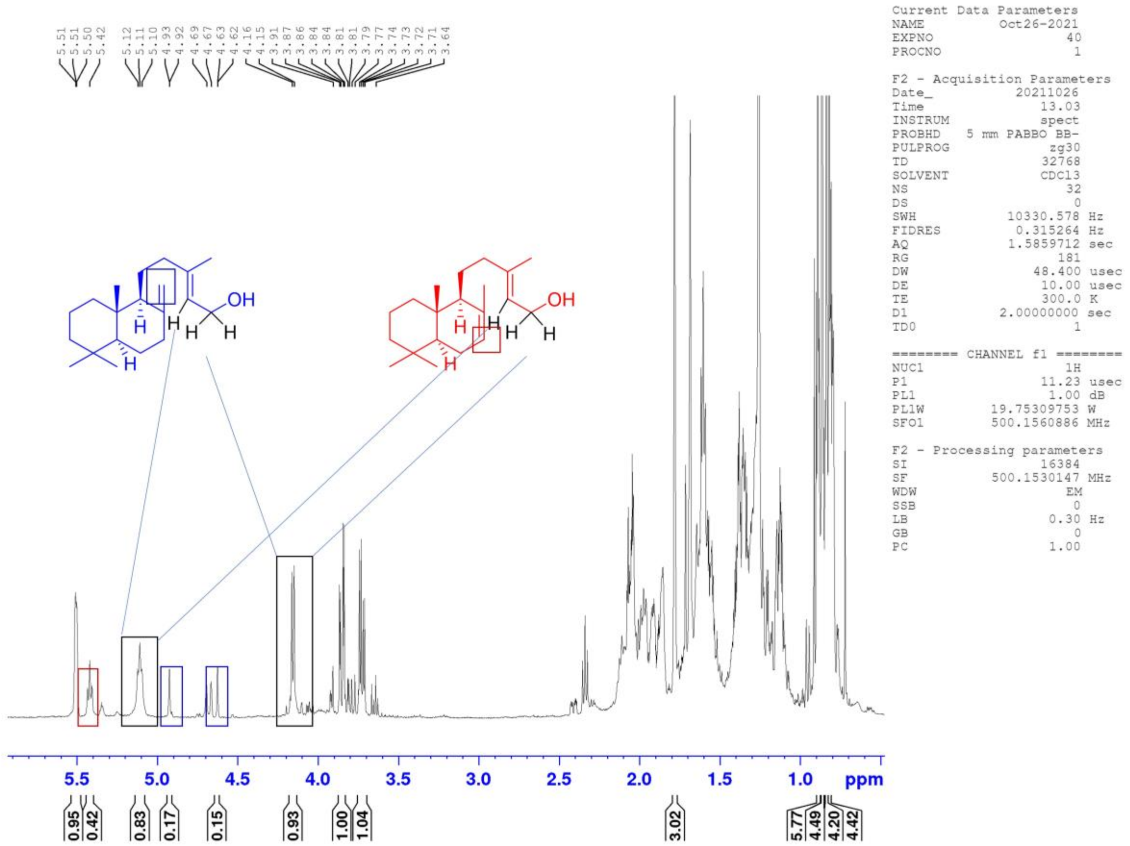

Supplementary Figure 51: ^{1}H of *ent*-isocopolol mix fraction for side product identification. Duplett at 4.15-4.16 = 2H next to the hydroxy group of α- and γ-labdane. Singletts at 4.62 and 4.92 = exocyclic double-bond of γ-labdane. Triplett at 5.1 = prenyl-double-bond-H. Triplett at 5.42 endocyclic double-bond-H of α-labdane product.

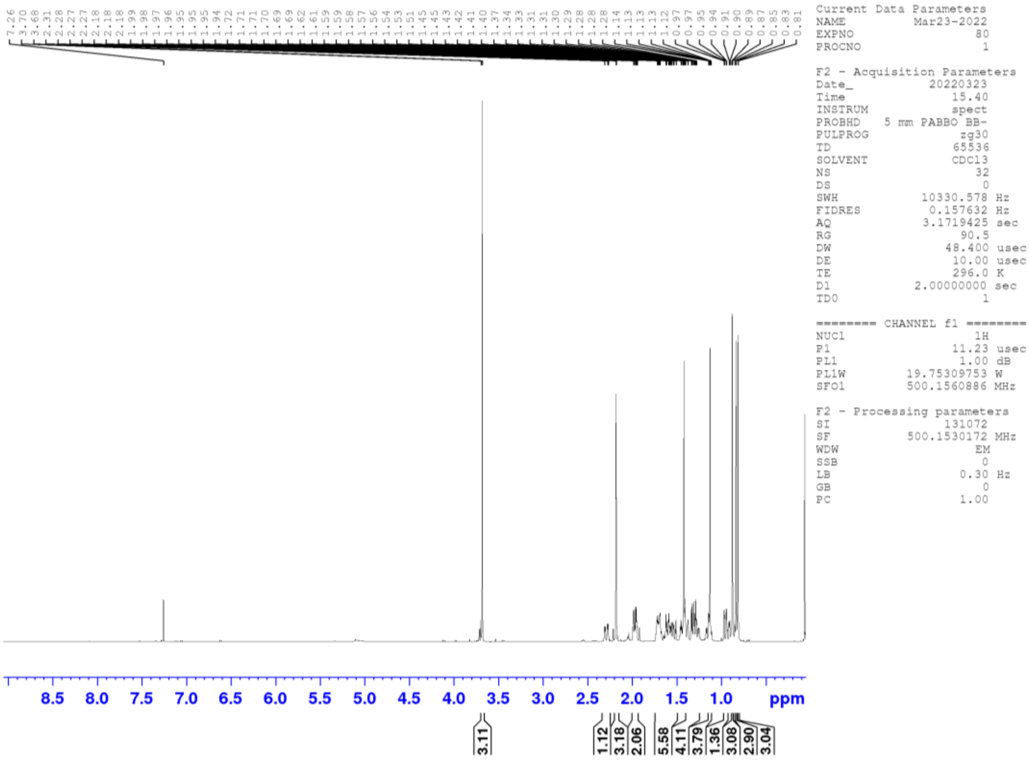

Supplementary Figure 52: ^{1}H of cyclic β-keto ester (16)

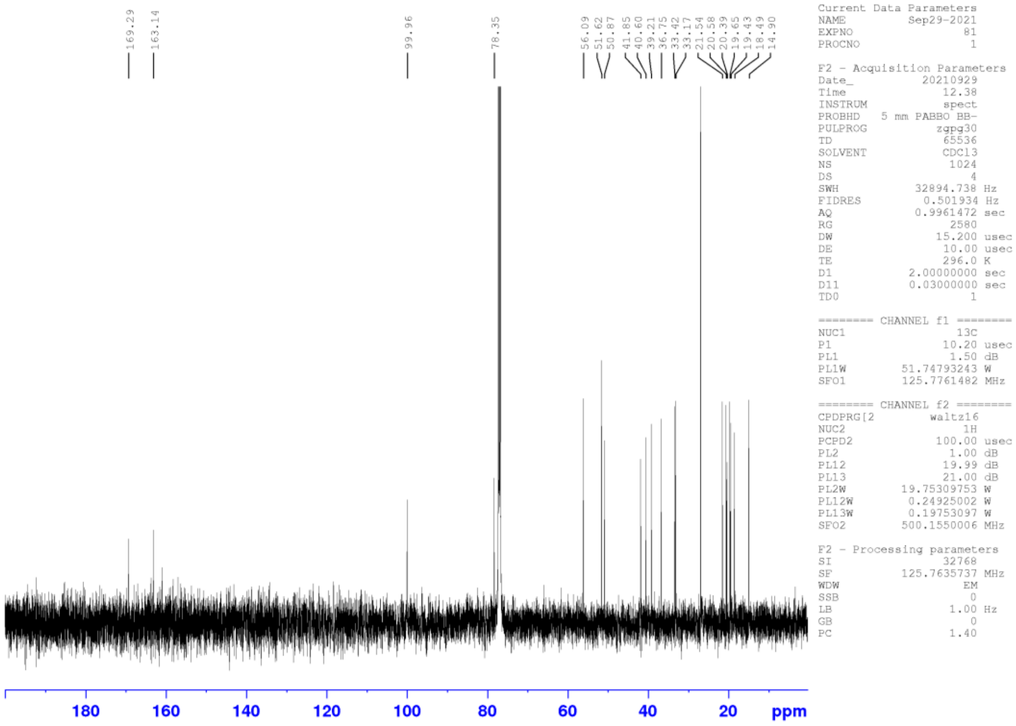

Supplementary Figure 53: ^{13}C of cyclic β-keto ester (16)

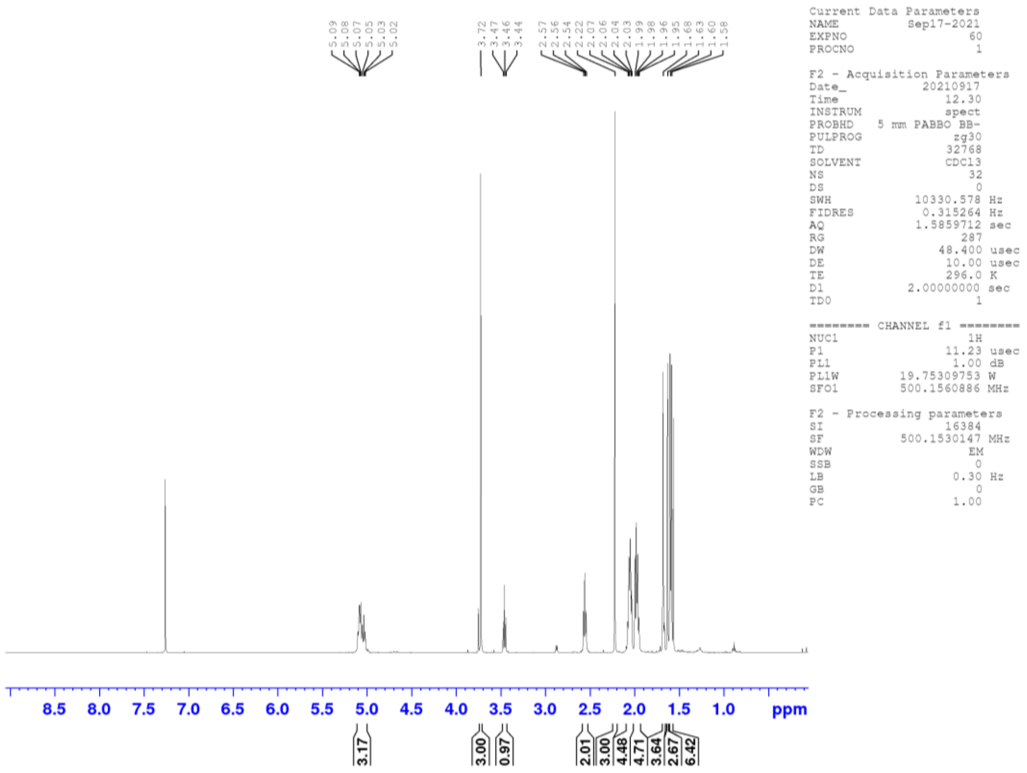

Supplementary Figure 54: ^{1}H of β-keto ester (15)

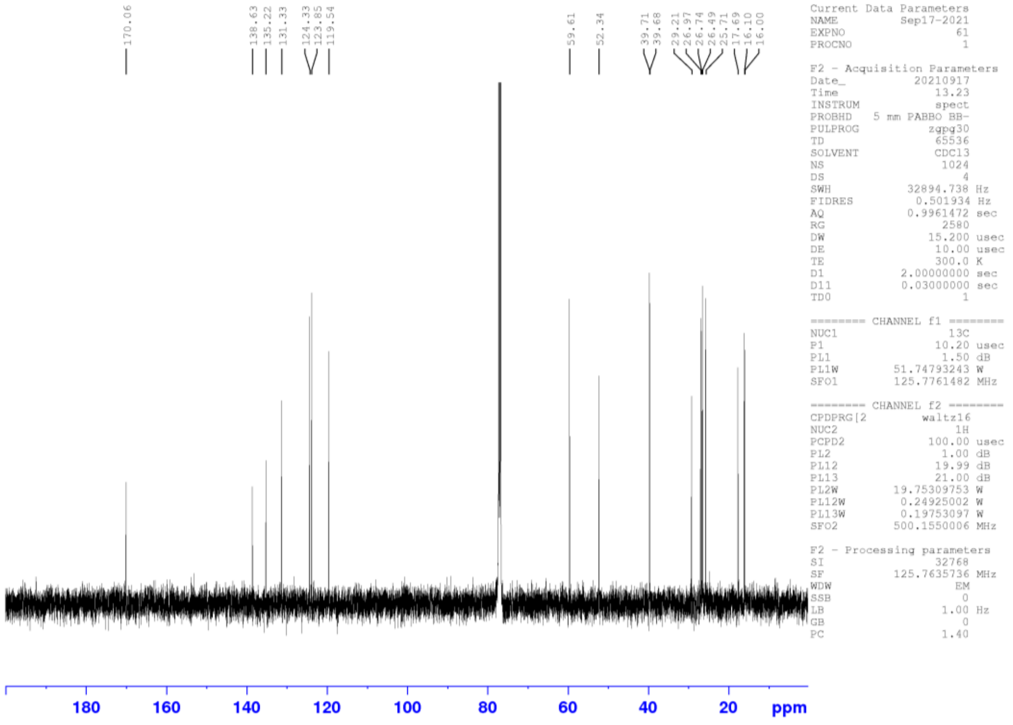

Supplementary Figure 55: ^{13}C of β-keto ester (15)

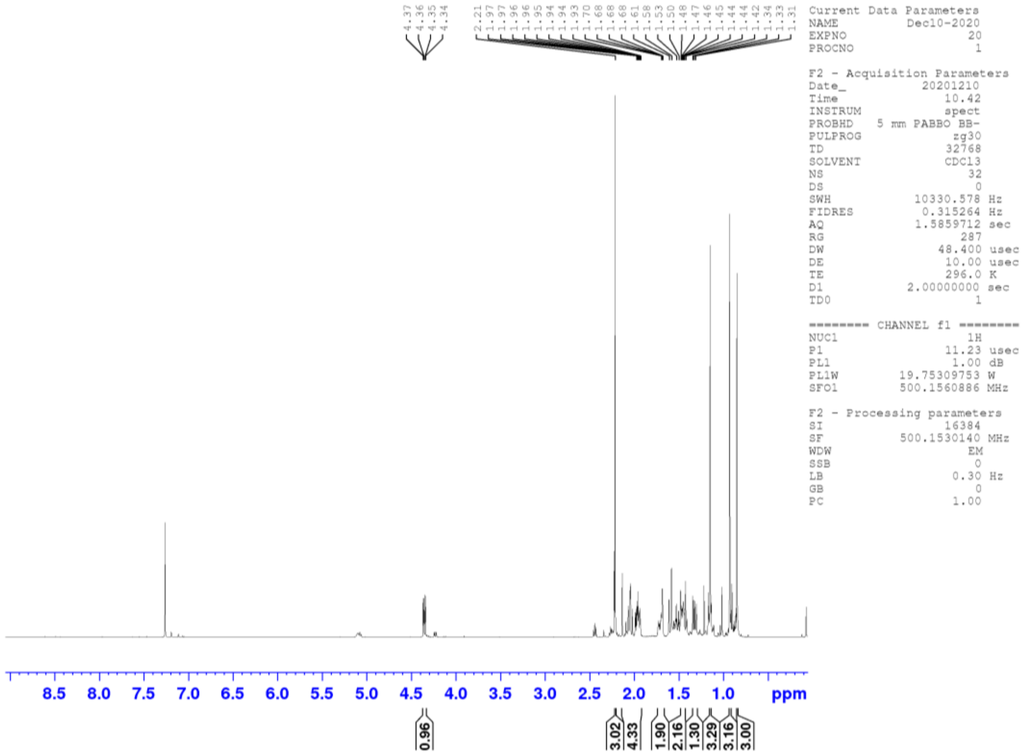

Supplementary Figure 56: ^{1}H of ketone intermediate (P-1)

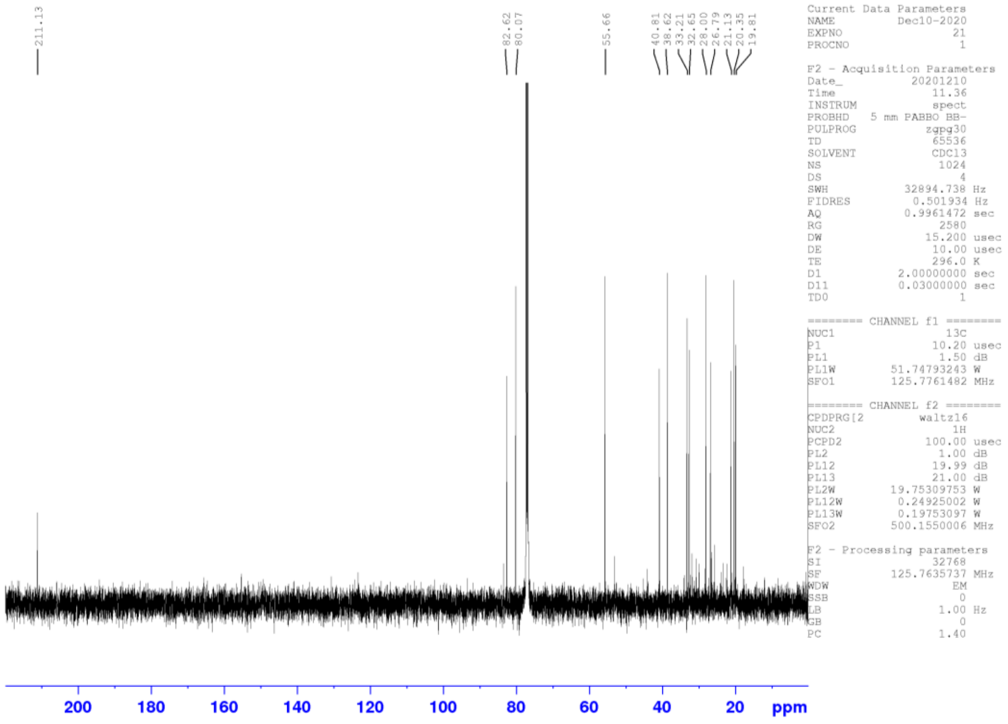

Supplementary Figure 57: ^{13}C of ketone intermediate (P-1)

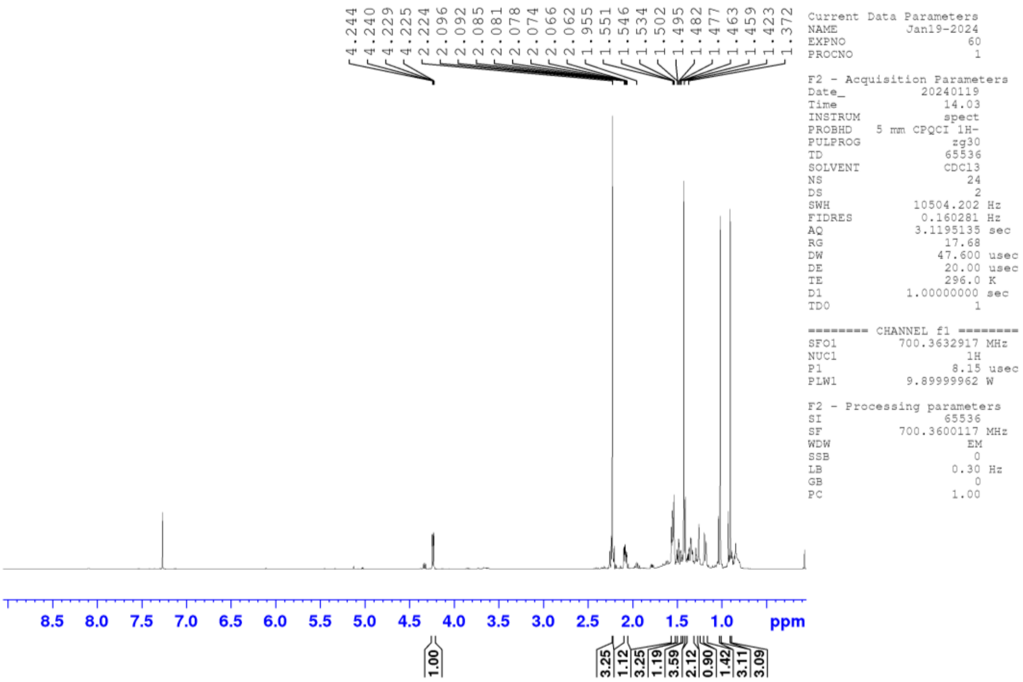

Supplementary Figure 58: ^{1}H of ketone intermediate (P-2)

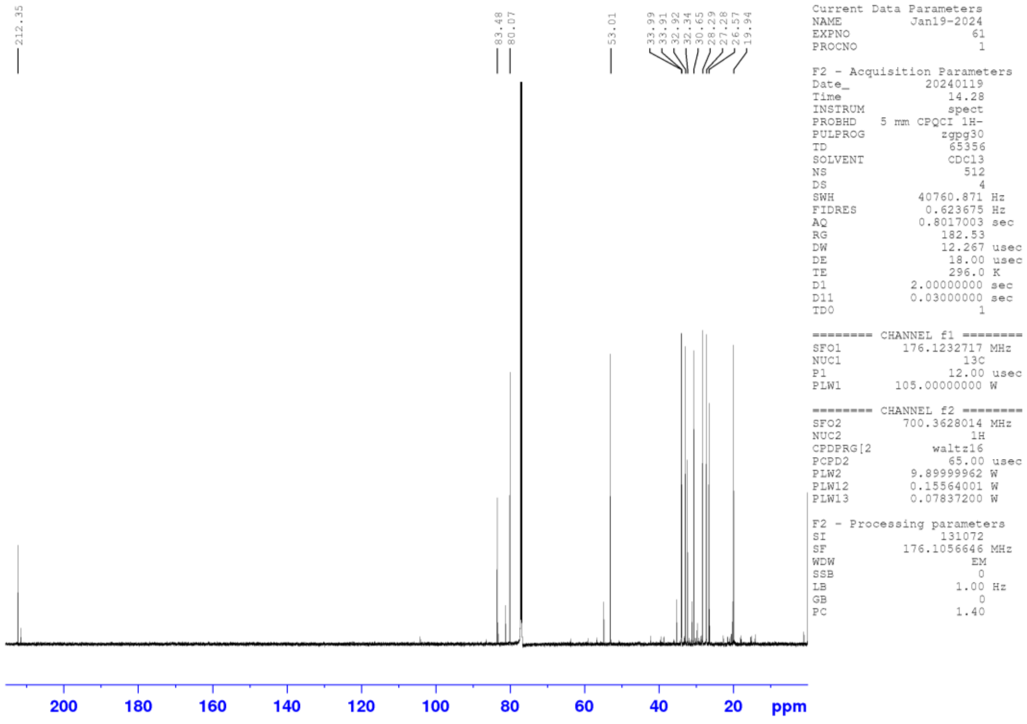

Supplementary Figure 59: ^{13}C of ketone intermediate (P-2)

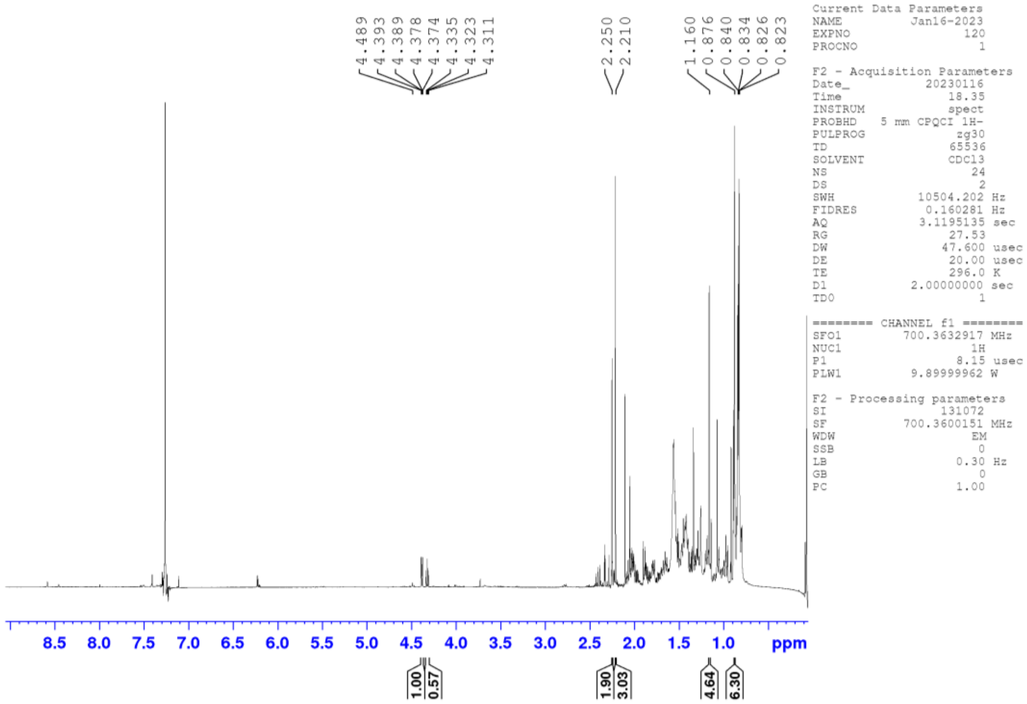

Supplementary Figure 60: ^{1}H of ketone intermediate (P-3)

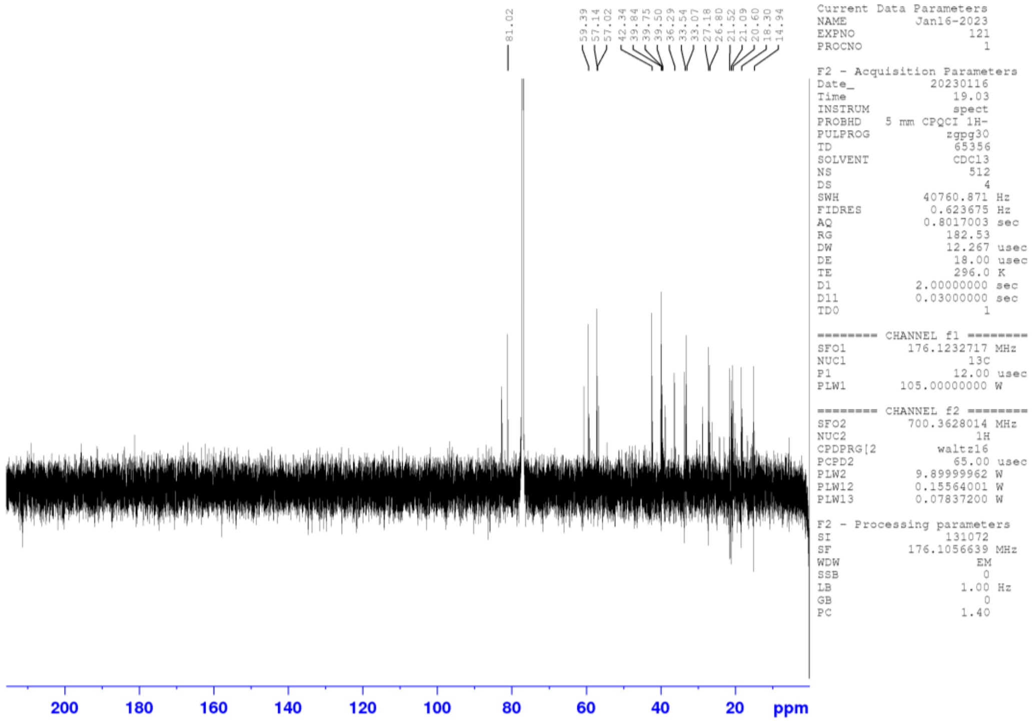

Supplementary Figure 61: ^{13}C of ketone intermediate (P-3)

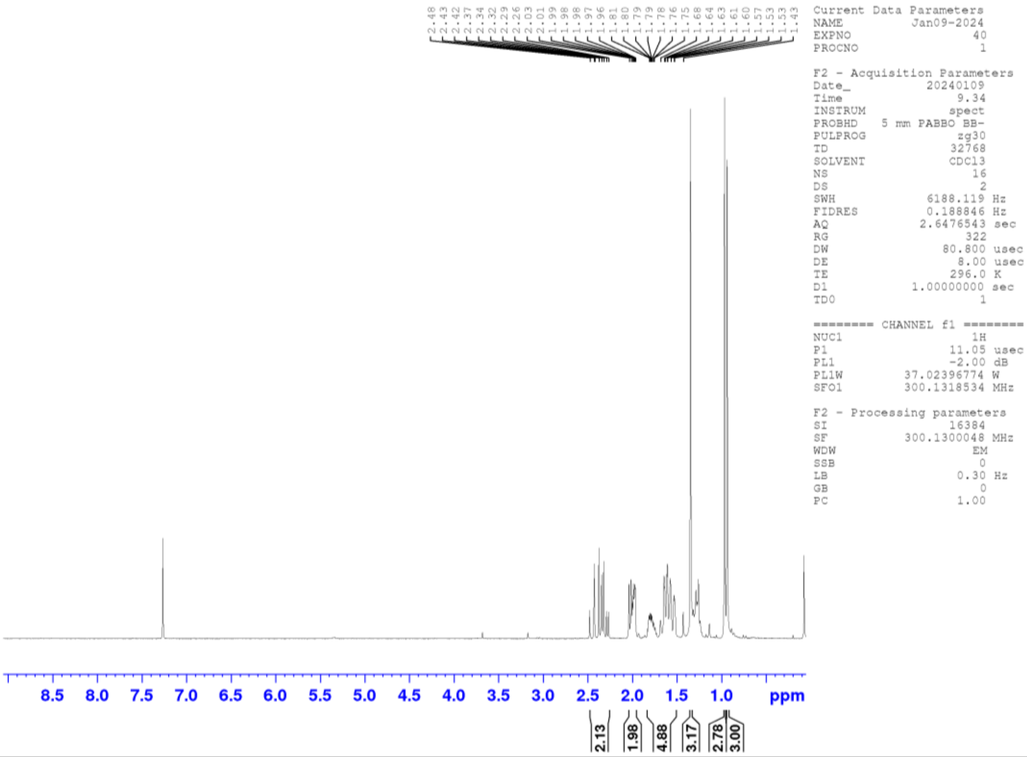

Supplementary Figure 62: ^{1}H of trans-tetrahydroactinidiolide (1)

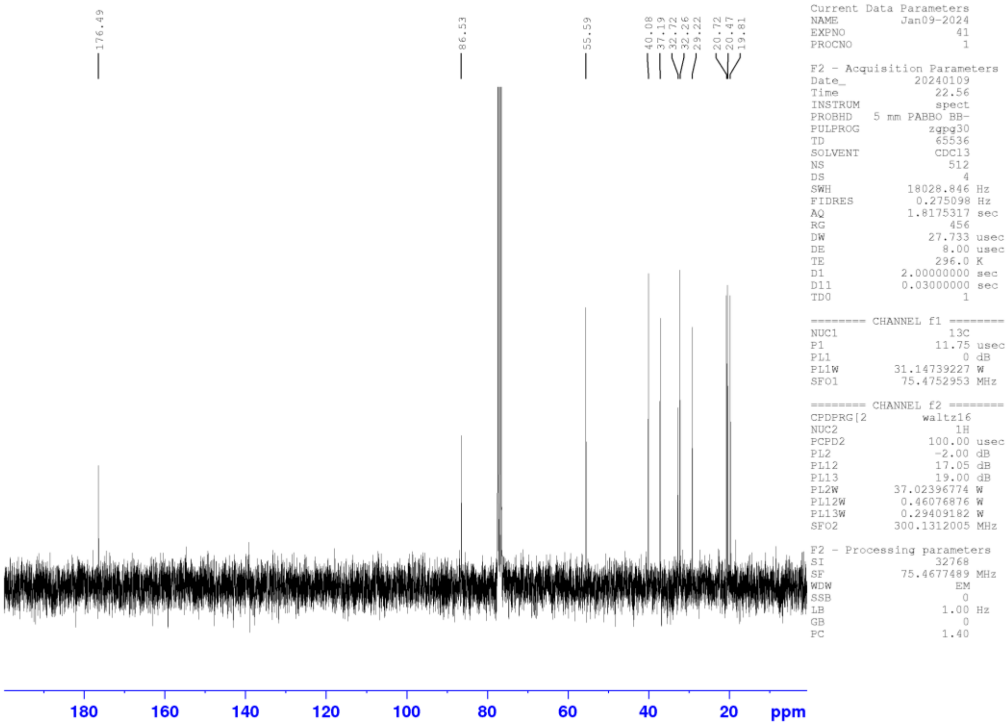

Supplementary Figure 63: ^{13}C of trans-tetrahydroactinidiolide (1)

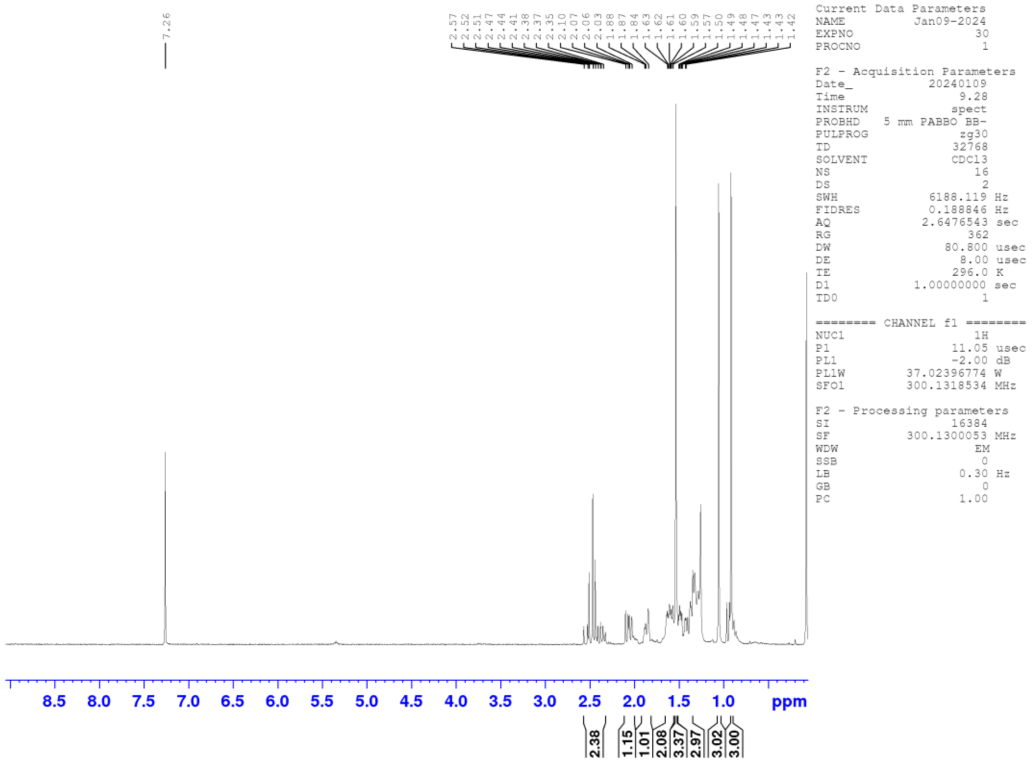

Supplementary Figure 64: ^{1}H of cis-tetrahydroactinidiolide (25)

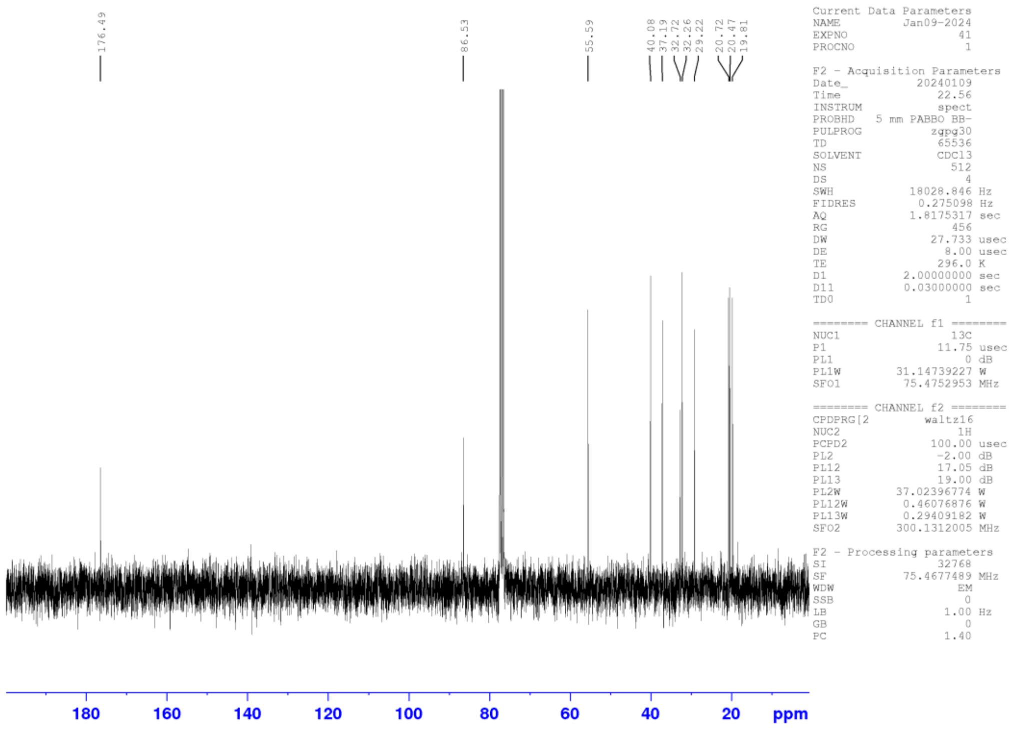

Supplementary Figure 65: ^{13}C of cis-tetrahydroactinidiolide (25)

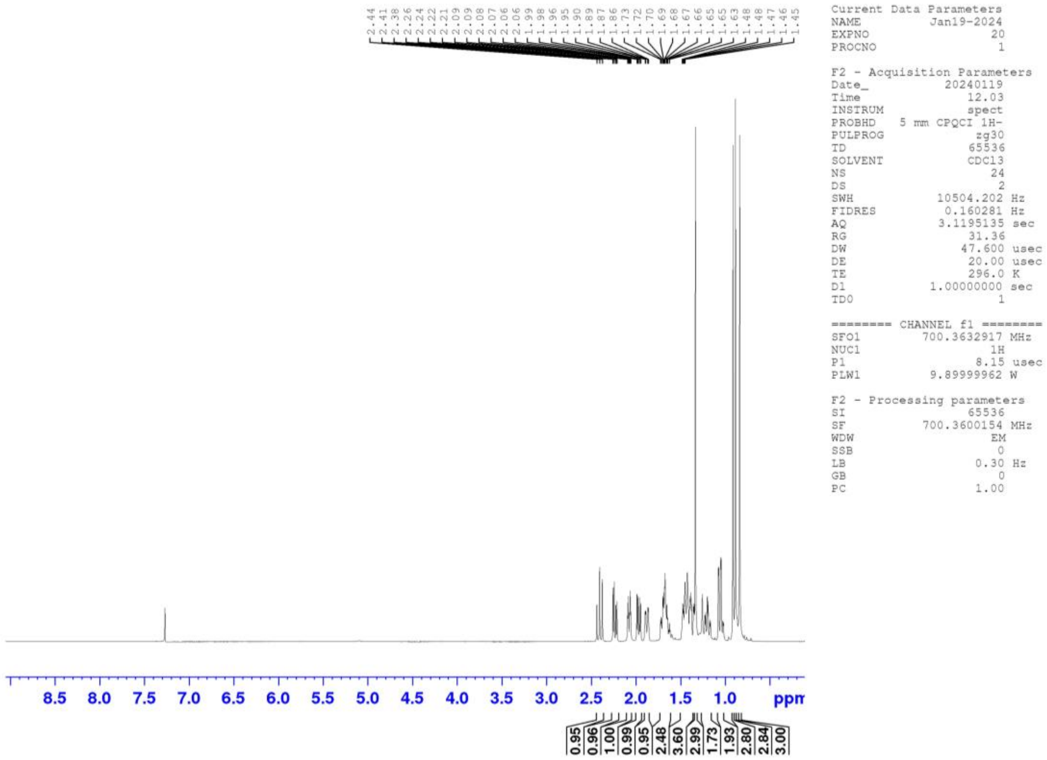

Supplementary Figure 66: ^{1}H of sclareolide (26)

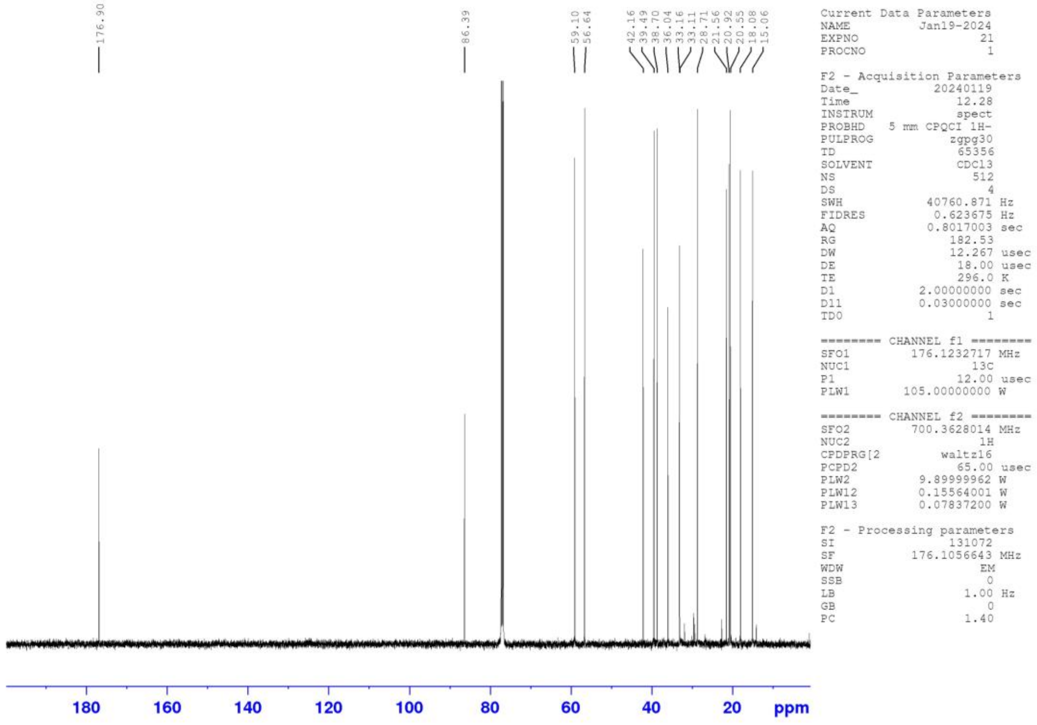

Supplementary Figure 67: ^{13}C of sclareolide (26)

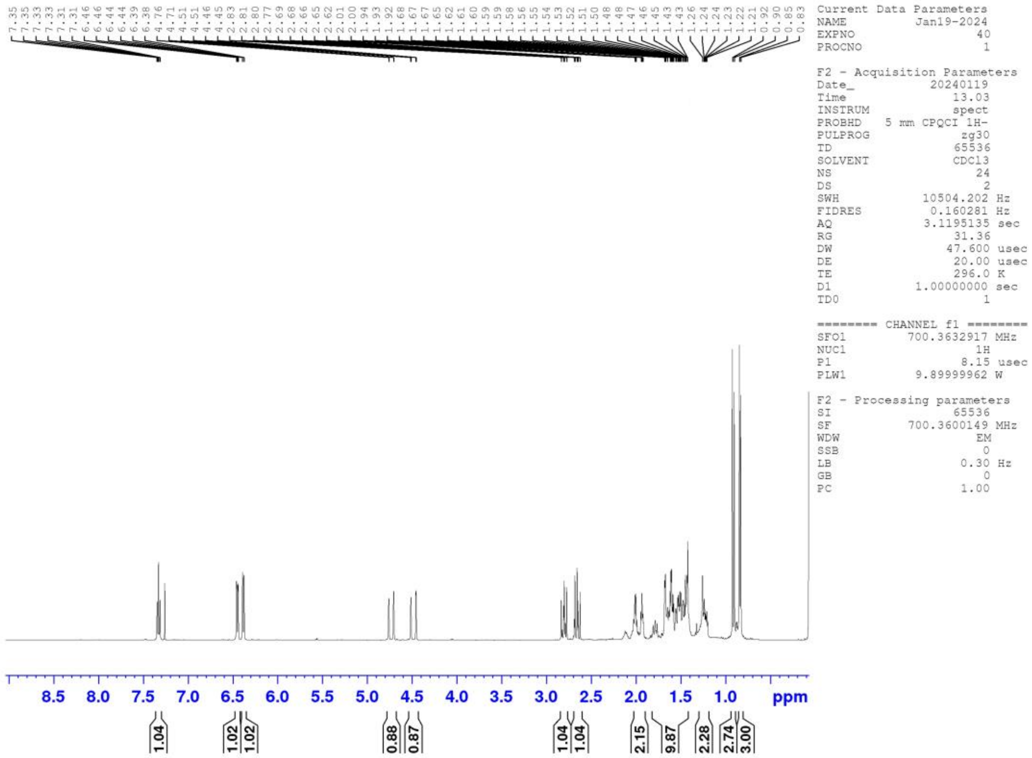

Supplementary Figure 68: ^{1}H of chromanone (3)

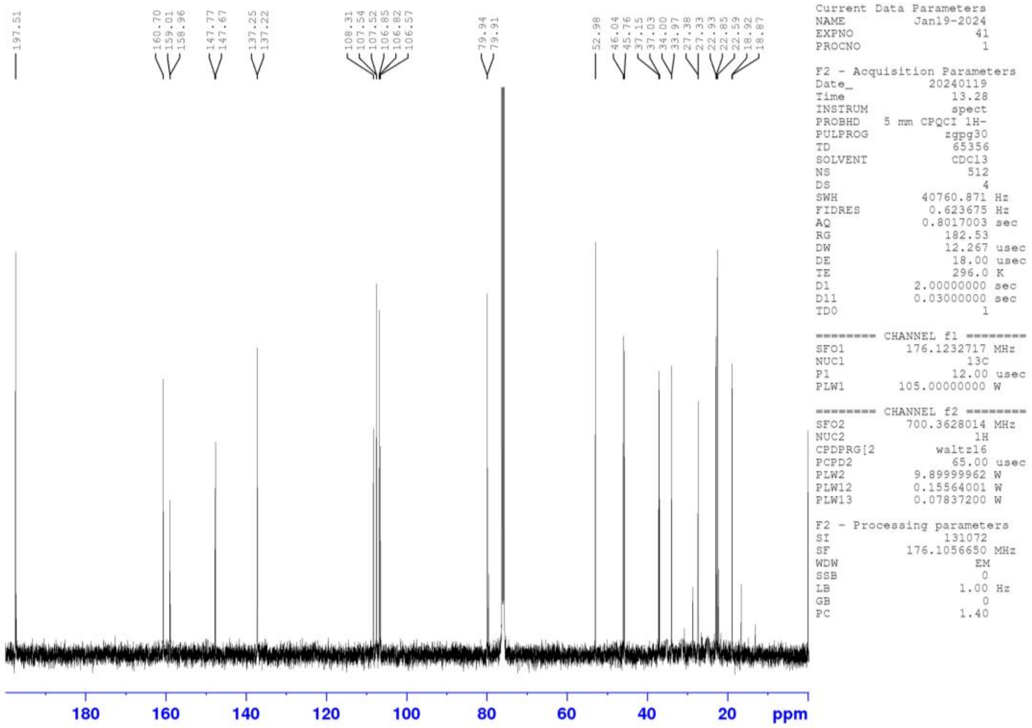

Supplementary Figure 69: ^{13}C of chromanone (3)

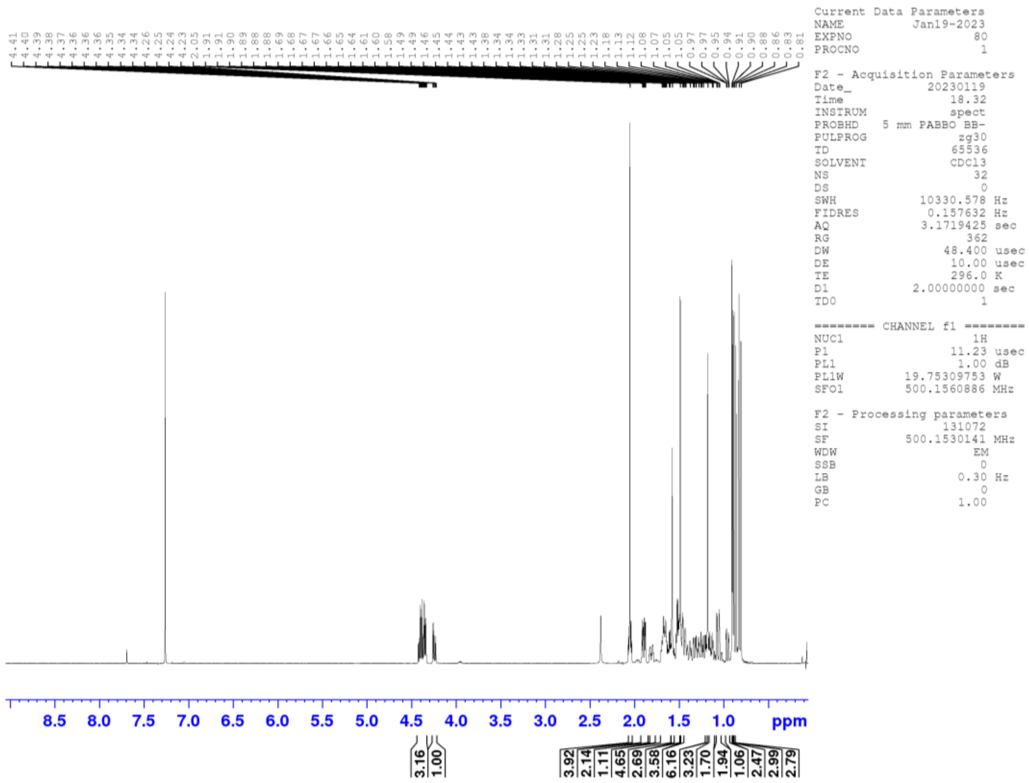

Supplementary Figure 70: ^{1}H of dioxanone (2) with drimenol acetate (S1-2) 1:1

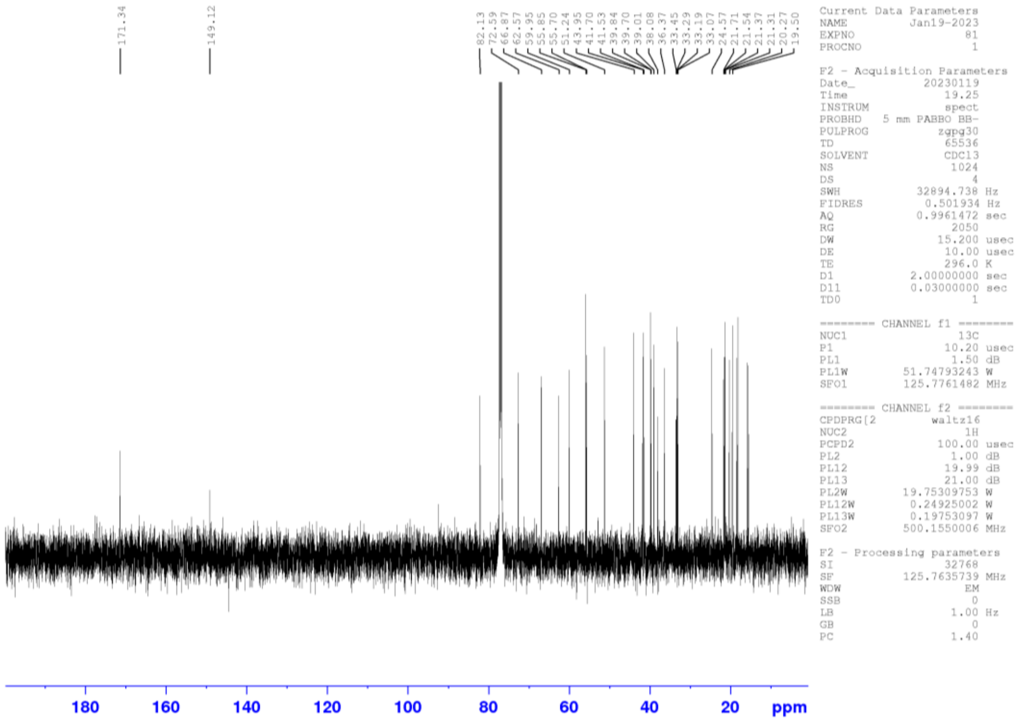

Supplementary Figure 71: ^{13}C of dioxanone (2) with drimenol acetate (S1-2) 1:1

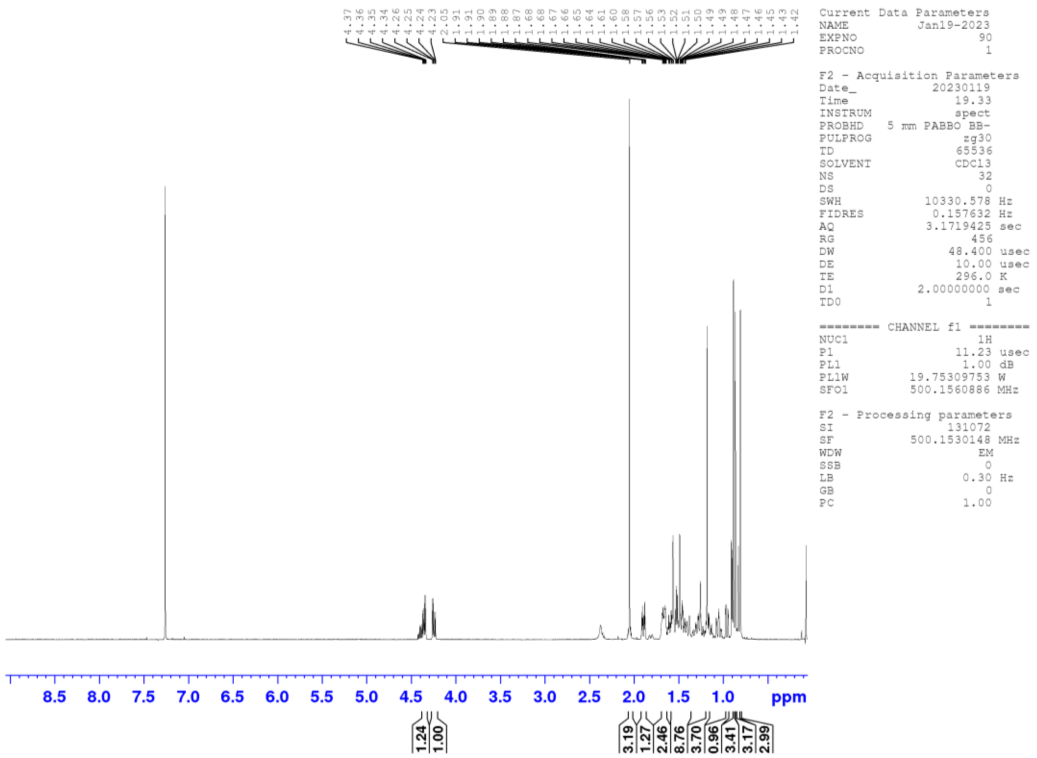

Supplementary Figure 72: ^{1}H of drimenol acetate (S1-2)

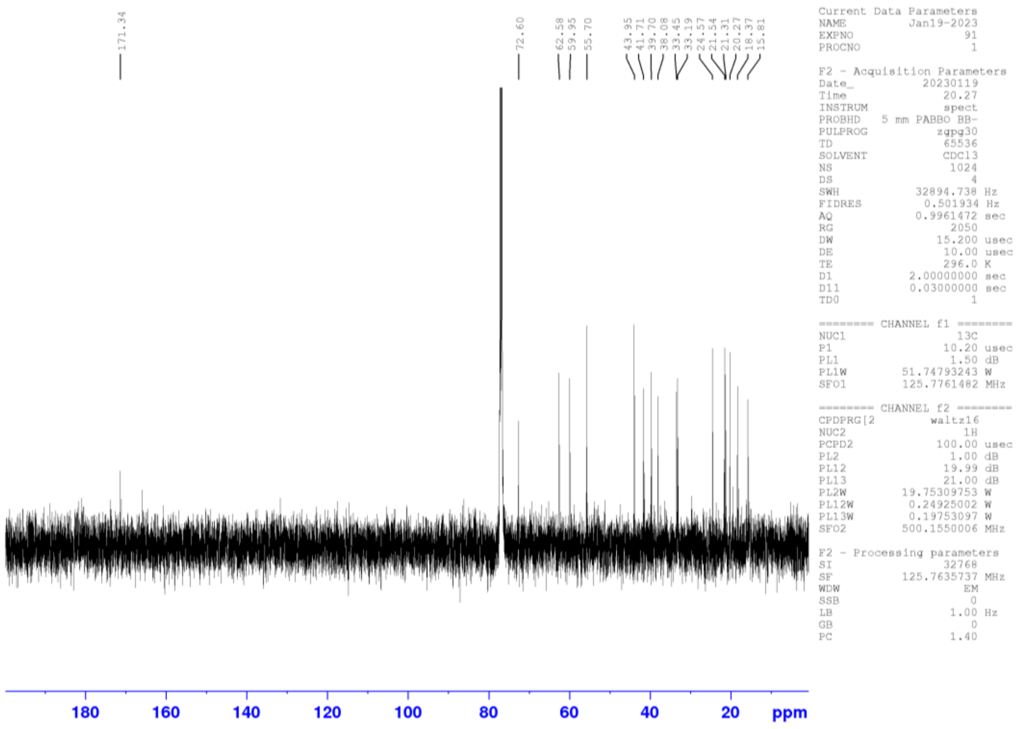

Supplementary Figure 73: ^{13}C of drimenol acetate (S1-2)

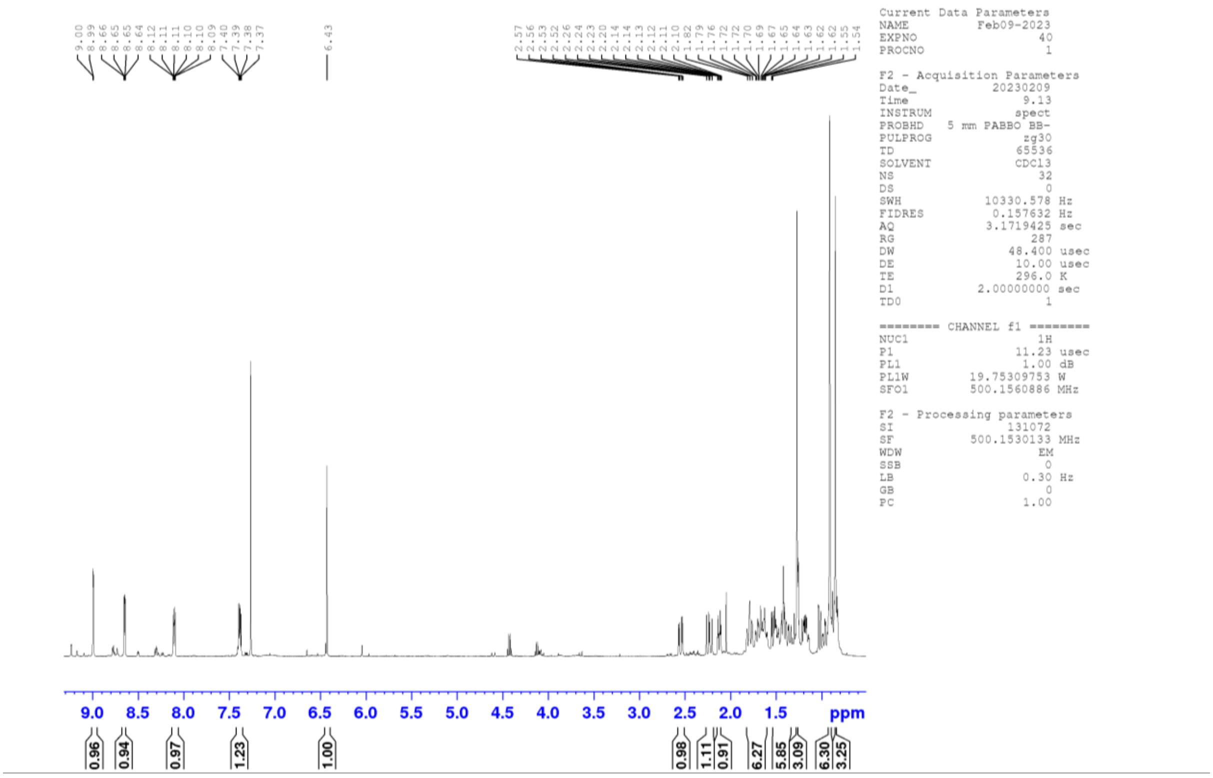

Supplementary Figure 74: ^{1}H of dehydroxy-pyripyropene (28)

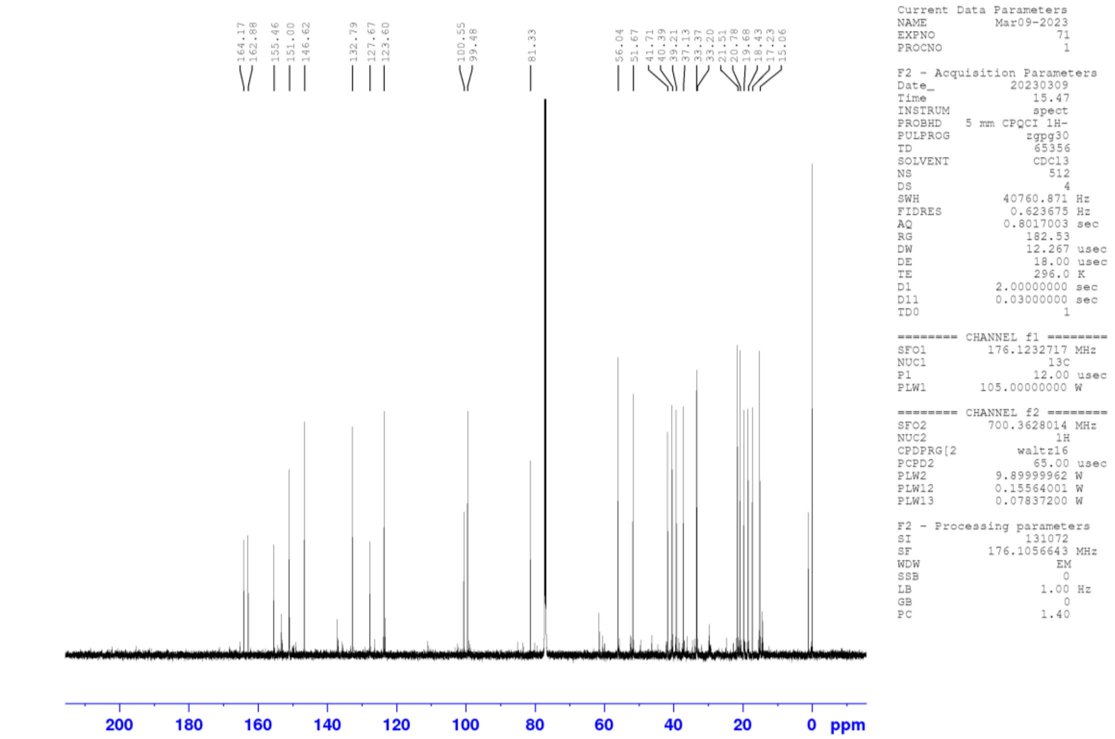

Supplementary Figure 75: ^{13}C of dehydroxy-pyripyropene (28)

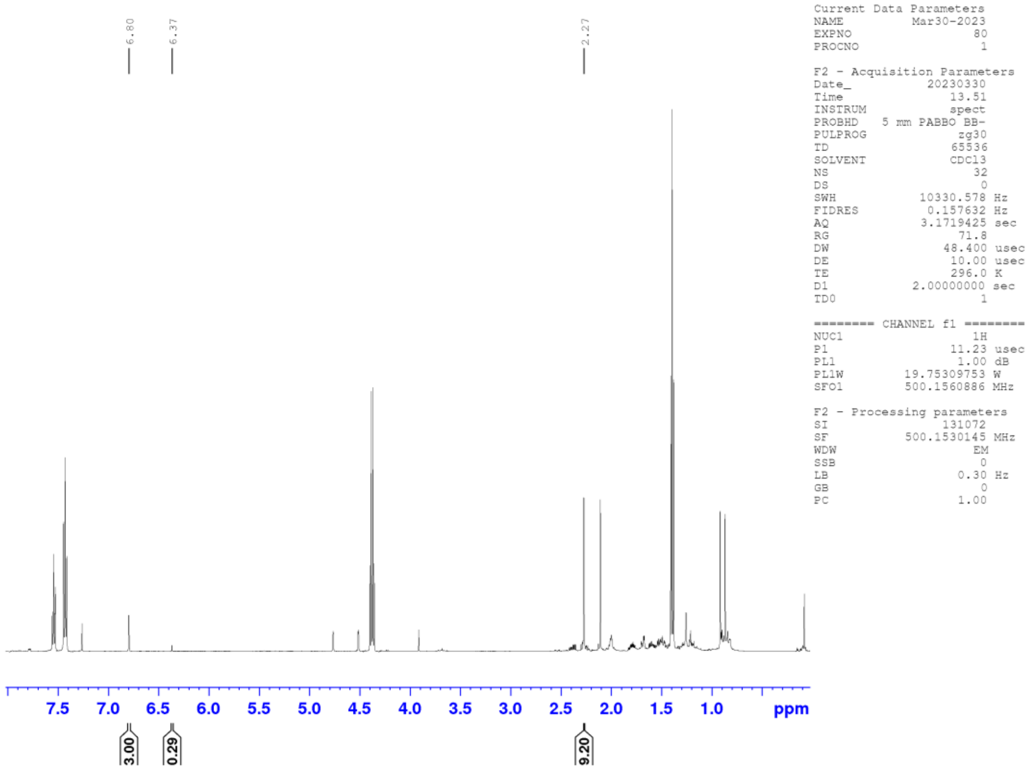

Supplementary Figure 76: ^{1}H of dihydroxy-phenylpyropene (29) with equimolar mesitylene standard

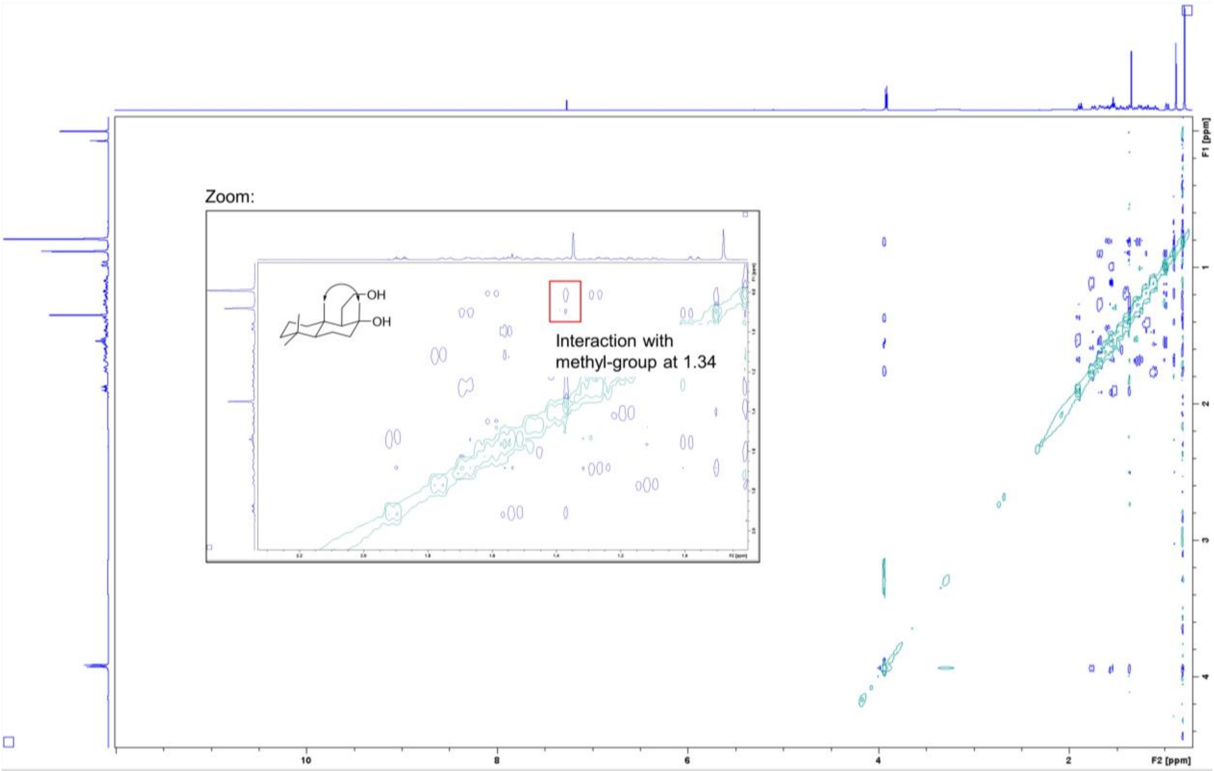

Supplementary Figure 77: NOESY of *S,R,S,S*-drimenol hydrate 9

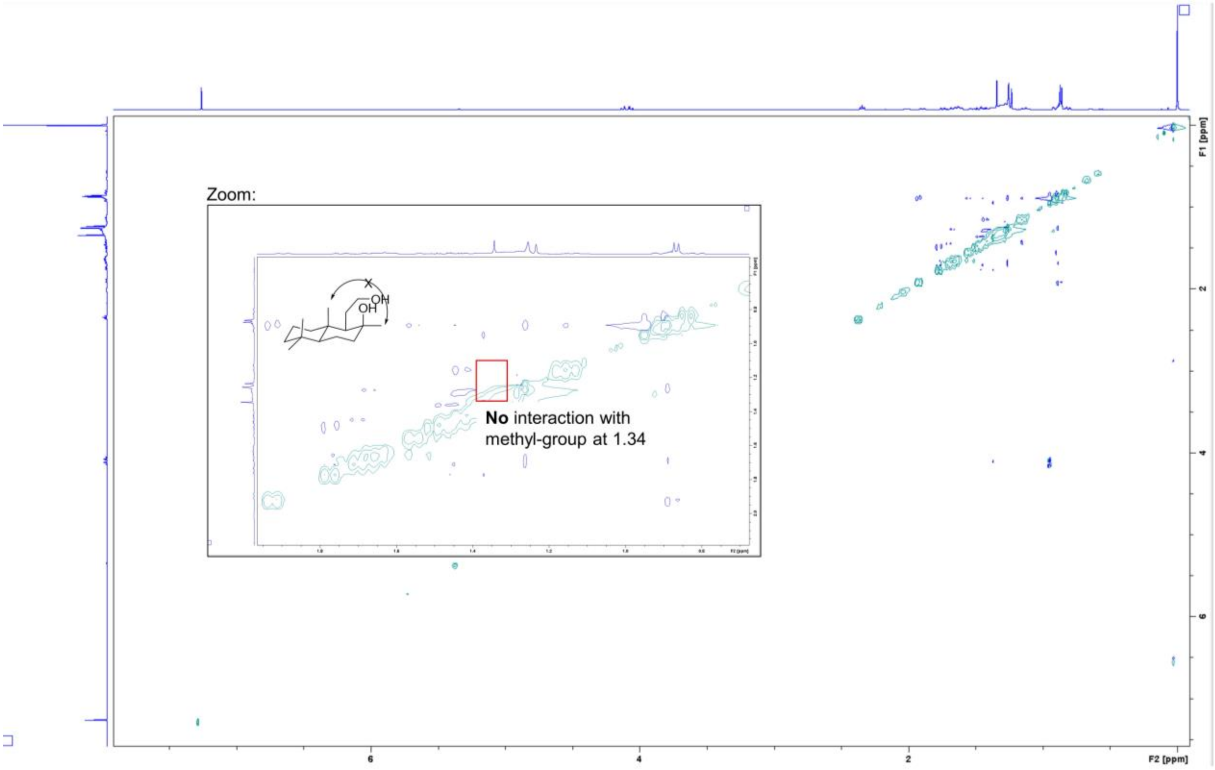

Supplementary Figure 78: NOESY of *S,S,S,S*-drimenol hydrate S1-9

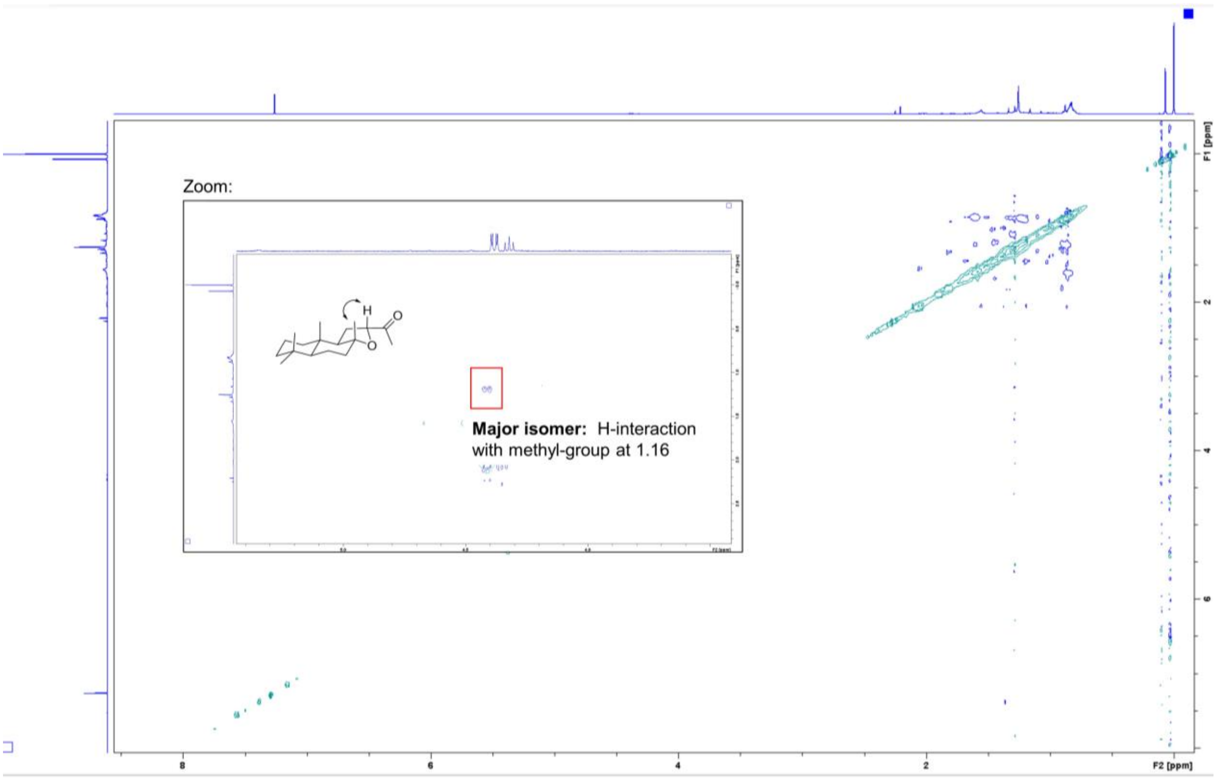

Supplementary Figure 79: NOESY of P-2

I. Amino acid sequences

*Aac*SHC

MAEQLVEAPAYARTLDRAVEYLLSCQKDEGYWWGPLLSNVTMEAEYVLLCHILDRVDRDR
MEKIRRYLLHEQREDGTWALYPGGPPDLDTTIEAYVALKYIGMSRDEEPMQKALRFIQSQG
GIESSRVFTRMWLALVGEYPWEKVPMVPPEIMFLGKRMPLNIYEFGSWARATVVAISIVMSR
QPVFPLPERARVPELYDTDVPPRRRGAKGGGGRIFDALDRALHGYQKLSVHPFRRAAEIRA
LDWLLERQAGDGSWGGIQPPWFYTLIALKILDMTQHPAFIKGWEGLELYGVDLDYGGWMF
QASISPVWDTGLAVLALRAAGLPADHDRLVKAGEWLLDRQITVPGDWAVKRPNLKPGGFAF
QFDNVYYPDVDDTAVVVWALNSLRLPDERRRRDVMTKGFRWIVGMQSSNGGWGAYDVD
NTSDLPNHIPFCDFGEVTDPPSEDVTAHVLECFGSFGYDDAWKVIRRAVEYLKREQRPDGS
WFGRWGVNYLYGTGAVVPALKAVGIDVREPFIQKALDWVEQHQNPDGGWGEDCRSYEDP
AYAGKGASTPSQTAWALMALIAGGRAESDSVRRGVQYLVETQRPDGGWDEPYYTGTGFP
GDFYLGYTMYRHVFPTLALGRYKQAIERR

*Tel*SHC

MPTSLATAIDPKQLQQAIRASQDFLFSQQYAEGYWWAELESNVTMTAEVILLHKIWGTEQRL
PLAKAEQYLRNHQRDHGGWELFYGDGGDLSTSVEAYMGLRLLGVPETDPALVKARQFILA
RGGISKTRIFTKLHLALIGCYDWRGIPSLPPWIMLLPEGSPFTIYEMSSWARSSTVPLLIVMDR
KPVYGMDPPITLDELYSEGRANVVWELPRQGDWRDVFIGLDRVFKLFETLNIHPLREQGLKA
AEEWVLERQEASGDWGGIIPAMLNSLLALRALDYAVDDPIVQRGMAAVDRFAIETETEYRVQ
PCVSPVWDTALVMRAMVDSGVAPDHPALVKAGEWLLSKQILDYGDWHIKNKKGRPGGWA
FEFENRFYPDVDDTAVVVMALHAVTLPNENLKRRAIERAVAWIASMQCRPGGWAAFDVDN
DQDWLNGIPYGDLKAMIDPNTADVTARVLEMVGRCQLAFDRVALDRALAYLRNEQEPEGC
WFGRWGVNYLYGTSGVLTALSLVAPRYDRWRIRRAAEWLMQCQNADGGWGETCWSYHD
PSLKGKGDSTASQTAWAIIGLLAAGDATGDYATEAIERGIAYLLETQRPDGTWHEDYFTGTG
FPCHFYLKYHYYQQHFPLTALGRYARWRNLLAT

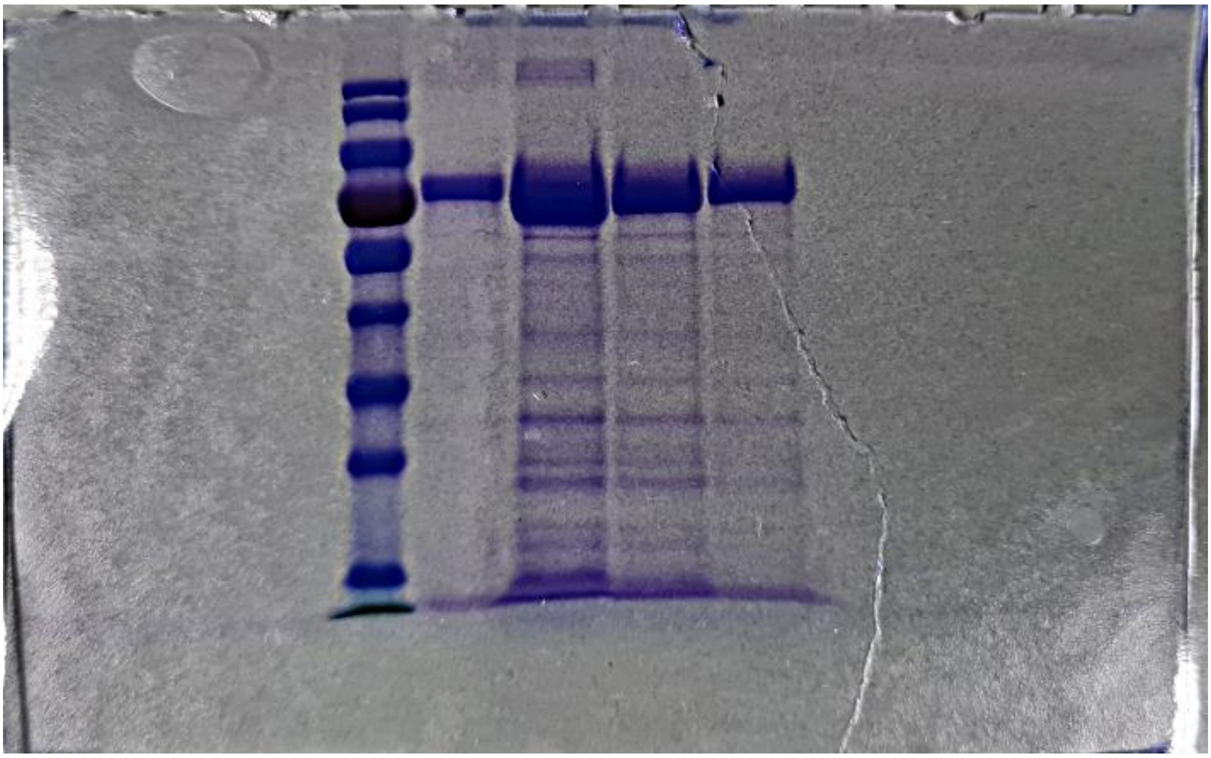

Supplementary Figure 80: Uncropped SDS-PAGE for Supplementary Figure 20.

J. Supplementary References

1. Li, J., Li, F., King-Smith, E. & Renata, H. Merging chemoenzymatic and radical-based retrosynthetic logic for rapid and modular synthesis of oxidized meroterpenoids. *Nat. Chem.* **12**, 173–179 (2020).

2. Basabe, P. *et al.* Synthesis of (+)-makassaric acid, a protein kinase MK2 inhibitor.

Tetrahedron **66**, 6008–6012 (2010).

3. Dethe, D. H., Murhade, G. M., Dherange, B. D. & Sau, S. K. Enantiospecific Syntheses of Hongoquercins A and B and Chromazonarol. *European J. Org. Chem.* **2017**, 1143–1150 (2017).

4. Yonemura, Y., Ohyama, T. & Hoshino, T. Chemo-enzymatic syntheses of drimane-type sesquiterpenes and the fundamental core of hongoquercin meroterpenoid by recombinant squalene-hopene cyclase. *Org. Biomol. Chem.* **10**, 440–446 (2012).

5. Ungarean, C. N., Southgate, E. H. & Sarlah, D. Enantioselective polyene cyclizations. *Org. Biomol. Chem.* **14**, 5454–5467 (2016).

6. Land, H. & Humble, M. S. YASARA: A tool to obtain structural guidance in biocatalytic investigations. in *Methods in Molecular Biology* vol. 1685 43–67 (Humana Press Inc., 2018).

7. Baek, M. & Baker, D. Deep learning and protein structure modeling. *Nat. Methods* **19**, 13–14 (2022).

8. Schneider, A., Jegl, P. & Hauer, B. Stereoselective Directed Cationic Cascades Enabled by Molecular Anchoring in Terpene Cyclases. *Angew. Chem. Int. Ed.* **60**, 13251–13256 (2021).

9. Schulte, S., Potter, K., Lemke, C. & Peters, R. J. Catalytic bases and stereo-control in Lamiaceae class II diterpene cyclases. *Biochemistry* **57**, 3473–3479 (2018).

10. Chovancova, E. *et al.* CAVER 3.0: A Tool for the Analysis of Transport Pathways in Dynamic Protein Structures. *PLOS Comput. Biol.* **8**, e1002708 (2012).

11. Schneider, A., Curado, C., Lystbaek, T. B., Osuna, S. & Hauer, B. Harnessing the Structure and Dynamics of the Squalene-Hopene Cyclase for (-)-Ambroxide Production. *Angew. Chem. Int. Ed.* e202301607 (2023) doi:10.1002/ANIE.202301607.

12. Reetz, M. T., Soni, P., Fernández, L., Gumulya, Y. & Carballeira, J. D. Increasing the stability of an enzyme toward hostile organic solvents by directed evolution based on iterative saturation mutagenesis using the B-FIT method. *Chem. Commun.* **46**, 8657–8658 (2010).

13. Lye, G. J. & Woodley, J. M. Application of in situ product-removal techniques to biocatalytic processes. *Trends Biotechnol.* **17**, 395–402 (1999).

14. Wachtmeister, J. & Rother, D. Recent advances in whole cell biocatalysis techniques bridging from investigative to industrial scale. *Curr. Opin. Biotechnol.* **42**, 169–177

(2016).

15. Los, D. A., Mironov, K. S. & Allakhverdiev, S. I. Regulatory role of membrane fluidity in gene expression and physiological functions. *Photosynth. Res.* **116**, 489–509 (2013).

16. Moulines, J., Bats, J. P., Lamidey, A. M. & Da Silva, N. About a practical synthesis of Ambrox® from sclareol: A new preparation of a ketone key intermediate and a close look at its Baeyer-Villiger oxidation. *Helv. Chim. Acta* **87**, 2695–2705 (2004).

17. Vlad, P. F., Ungur, N. D., Van Hung, N. & Perutsky, V. B. Superacidic low-temperature cyclization of terpenols and their acetates. *Russ. Chem. Bull.* **44**, 2390–2403 (1995).

18. Kulchitski, V. N., Ungur, N. D. & Vlad, P. F. Electrophilic cyclization of α- and β-geranyl acetates by mercury(II) trifluoroacetate. *Russ. Chem. Bull.* **46**, 1264–1268 (1997).

19. Tsangarakis, C. & Stratakis, M. Biomimetic cyclization of small terpenoids promoted by zeolite NaY: Tandem formation of α-ambrinol from geranyl acetone. *Adv. Synth. Catal.* **347**, 1280–1284 (2005).

20. Ungur, N. D., Popa, N. P., Van Tuen, N. & Vlad, P. F. Cyclization of α-terpenols and their acetates by fluorosulfonic acid. *Chem. Nat. Compd.* **29**, 473–478 (1993).

21. Grin'ko, M., Kul'chitskii, V., Ungur, N. & Vlad, P. F. Superacid cyclization of certain aliphatic sesquiterpene derivatives in ionic liquids. *Chem. Nat. Compd.* **42**, 439–441 (2006).

22. Parker, K. A. & Resnick, L. The First Total Synthesis of a Pyripyropene-Type ACAT Inhibitor, (±)-GERI-BP00P. *J. Org. Chem* **60**, 5726–5728 (1995).

23. Gibson, D. G. *et al.* Enzymatic assembly of DNA molecules up to several hundred kilobases. *Nat. Methods* **6**, 343–345 (2009).

24. Renzette, N. Generation of transformation competent E. coli. *Curr. Protoc. Microbiol.* **22**, A.3L.1-A.3L.5 (2011).

25. Ren, X., Yu, D., Han, S. & Feng, Y. Thermolysis of recombinant *Escherichia coli* for recovering a thermostable enzyme. *Biochem. Eng. J.* **33**, 94–98 (2007).

26. Koschorreck, K., Wahrendorff, F., Biemann, S., Jesse, A. & Urlacher, V. B. Cell thermolysis – A simple and fast approach for isolation of bacterial laccases with potential to decolorize industrial dyes. *Process Biochem.* **56**, 171–176 (2017).

27. Pol-Fachin, L., Rusu, V. H., Verli, H. & Lins, R. D. GROMOS 53A6 GLYC, an improved GROMOS force field for hexopyranose-based carbohydrates. *J. Chem. Theory Comput.* **8**, 4681–4690 (2012).

28. Oostenbrink, C., Villa, A., Mark, A. E. & Van Gunsteren, W. F. A biomolecular force field based on the free enthalpy of hydration and solvation: The GROMOS force-field parameter sets 53A5 and 53A6. *J. Comput. Chem.* **25**, 1656–1676 (2004).

29. Poger, D., Van Gunsteren, W. F. & Mark, A. E. A new force field for simulating phosphatidylcholine bilayers. *J. Comput. Chem.* **31**, 1117–1125 (2010).

30. Schmid, N. *et al.* Definition and testing of the GROMOS force-field versions 54A7 and 54B7. *Eur. Biophys. J.* **40**, 843–856 (2011).

31. Reif, M. M., Hünenberger, P. H. & Oostenbrink, C. New interaction parameters for charged amino acid side chains in the GROMOS force field. *J. Chem. Theory Comput.* **8**, 3705–3723 (2012).

32. Lins, R. D. & Hünenberger, P. H. A new GROMOS force field for hexopyranose-based carbohydrates. *J. Comput. Chem.* **26**, 1400–1412 (2005).

33. Hansen, H. S. & Hünenberger, P. H. A reoptimized GROMOS force field for hexopyranose-based carbohydrates accounting for the relative free energies of ring conformers, anomers, epimers, hydroxymethyl rotamers, and glycosidic linkage conformers. *J. Comput. Chem.* **32**, 998–1032 (2011).

34. Malde, A. K. *et al.* An Automated force field Topology Builder (ATB) and repository: Version 1.0. *J. Chem. Theory Comput.* **7**, 4026–4037 (2011).

35. Berendsen, H. J. C., Postma, J. P. M., van Gunsteren, W. F. & Hermans, J. *Intermolecular Forces*. (1981).

36. MacCallum, J. L., Drew Bennett, W. F. & Peter Tieleman, D. Distribution of Amino Acids in a Lipid Bilayer from Computer Simulations. *Biophys. J.* **94**, 3393–3404 (2008).

37. Gebhardt, J., Kleist, C., Jakobtorweihen, S. & Hansen, N. Validation and Comparison of Force Fields for Native Cyclodextrins in Aqueous Solution. *J. Phys. Chem. B* **122**, 1608–1626 (2018).

38. Gebhardt, J. & Hansen, N. Calculation of binding affinities for linear alcohols to α-cyclodextrin by twin-system enveloping distribution sampling simulations. *Fluid Phase Equilib.* **422**, 1–17 (2016).

39. Markthaler, D., Gebhardt, J., Jakobtorweihen, S. & Hansen, N. Molecular Simulations of Thermodynamic Properties for the System α-Cyclodextrin/Alcohol in Aqueous Solution. *Chemie Ing. Tech.* **89**, 1306–1314 (2017).

40. Baz, J., Gebhardt, J., Kraus, H., Markthaler, D. & Hansen, N. Insights into Noncovalent

Binding Obtained from Molecular Dynamics Simulations. *Chemie Ing. Tech.* **90**, 1864–1875 (2018).

41. Abraham, M. J. *et al.* GROMACS: High performance molecular simulations through multi-level parallelism from laptops to supercomputers. *SoftwareX* **1–2**, 19–25 (2015).

42. Tribello, G. A., Bonomi, M., Branduardi, D., Camilloni, C. & Bussi, G. PLUMED 2: New feathers for an old bird. *Comput. Phys. Commun.* **185**, 604–613 (2014).

43. Nosé, S. & Nosi~, S. A molecular dynamics method for simulations in the canonical ensemble. *http://dx.doi.org/10.1080/00268978400101201* **52**, 255–268 (2006).

44. Hoover, W. G. Canonical dynamics: Equilibrium phase-space distributions. *Phys. Rev. A* **31**, 1695 (1985).

45. Parrinello, M. & Rahman, A. Polymorphic transitions in single crystals: A new molecular dynamics method. *J. Appl. Phys.* **52**, 7182 (1998).

46. Páll, S. & Hess, B. A flexible algorithm for calculating pair interactions on SIMD architectures. *Comput. Phys. Commun.* **184**, 2641–2650 (2013).

47. Essmann, U. *et al.* A smooth particle mesh Ewald method. *J. Chem. Phys.* **103**, 8577 (1998).

48. Hess, B., Bekker, H., Berendsen, H. J. C. & Fraaije, J. G. E. M. LINCS: A Linear Constraint Solver for Molecular Simulations. *J Comput Chem* **18**, 14631472 (1997).

49. Deng, Y. & Roux, B. Computations of standard binding free energies with molecular dynamics simulations. *J. Phys. Chem. B* **113**, 2234–2246 (2009).

50. Hermans, J. & Wang, L. Inclusion of Loss of Translational and Rotational Freedom in Theoretical Estimates of Free Energies of Binding. Application to a Complex of Benzene and Mutant T4 Lysozyme. (1997) doi:10.1021/JA963568.

51. Sugita, Y., Kitao, A. & Okamoto, Y. Multidimensional replica-exchange method for free-energy calculations. *J. Chem. Phys.* **113**, 6042 (2000).

52. Bussi, G. Hamiltonian replica exchange in GROMACS: a flexible implementation. *https://doi.org/10.1080/00268976.2013.824126* **112**, 379–384 (2013).

53. Shirts, M. R. & Chodera, J. D. Statistically optimal analysis of samples from multiple equilibrium states. *J. Chem. Phys.* **129**, 124105 (2008).

54. Klimovich, P. V., Shirts, M. R. & Mobley, D. L. Guidelines for the analysis of free energy calculations. *J. Comput. Aided. Mol. Des.* **29**, 397–411 (2015).

55. Markthaler, D., Jakobtorweihen, S. & Hansen, N. Lessons Learned from the Calculation of One-Dimensional Potentials of Mean Force [Article v1.0]. *Living J. Comput. Mol. Sci.* **1**, 11073–11073 (2019).

56. Reinert, D. J., Balliano, G. & Schulz, G. E. Conversion of Squalene to the Pentacarbocyclic Hopene. *Chem. Biol.* **11**, 121–126 (2004).

57. Seitz, M. *et al.* Synthesis of Heterocyclic Terpenoids by Promiscuous Squalene-Hopene Cyclases. *ChemBioChem* **14**, 436–439 (2013).

58. Bolster, M. G., Jansen, B. J. M. & De Groot, A. The synthesis of (-)-Ambrox® starting from labdanolic acid. *Tetrahedron* **57**, 5657–5662 (2001).

59. Serra, S. An expedient preparation of enantio-enriched ambergris odorants starting from commercial ionone alpha. *Flavour Fragr. J.* **28**, 46–52 (2013).

60. Kulciţki, V., Ungur, N., Gavagnin, M., Carbone, M. & Cimino, G. Further Synthetic Studies Towards the Austrodorane Skeleton: Synthesis of Austrodoral. *European J. Org. Chem.* **2005**, 1816–1822 (2005).

61. Tanimoto, H. & Oritani, T. Practical synthesis of Ambrox® from farnesyl acetate involving lipase catalyzed resolution. *Tetrahedron: Asymmetry* **7**, 1695–1704 (1996).

62. Urban, S. & Capon, R. J. Absolute stereochemistry of puupehenone and related metabolites. *J. Nat. Prod.* **59**, 900–901 (1996).

63. Novaes, L. F. T., Gonçalves, K. D. A., Trivella, D. B. B. & Pastre, J. C. Formal Total Synthesis of Actinoranone: Synthesis Approaches and Cytotoxic Studies. *J. Org. Chem.* **83**, 5160–5176 (2018).

64. Hoshino, T., Kumai, Y., Kudo, I., Nakano, S. I. & Ohashi, S. Enzymatic cyclization reactions of geraniol, farnesol and geranylgeraniol, and those of truncated squalene analogs having C20 and C25 by recombinant squalene cyclase. *Org. Biomol. Chem.* **2**, 2650–2657 (2004).

65. Basabe, P. *et al.* Synthesis of three marine natural sesterterpenolides from methyl isoanticopalate. First enantioselective synthesis of luffolide. *J. Org. Chem.* **70**, 9480–9485 (2005).

66. Imamura, P. M. & Santiago, G. M. P. Chlorosulfonic acid mediated cyclization of homoterpenic acid. *Synth. Commun.* **27**, 2479–2485 (1997).

67. Shulcts, E. E., Mironov, M. E., Kharitonov, Y. V & Vorozhtsov, N. N. FURANODITERPENOIDS OF THE LABDANE SERIES: OCCURRENCE IN PLANTS,

TOTAL SYNTHESIS, SEVERAL TRANSFORMATIONS, AND BIOLOGICAL ACTIVITY. *Chem. Nat. Compd.* **50**, 5–22 (2014).

68. Serra, S. & Piccioni, O. A new chemo-enzymatic approach to the stereoselective synthesis of the flavors tetrahydroactinidiolide and dihydroactinidiolide. *Tetrahedron Asymmetry* **26**, 584–592 (2015).
